# Ku70 senses cytosolic DNA and assembles a tumor-suppressive signalosome

**DOI:** 10.1126/sciadv.adh3409

**Published:** 2024-01-26

**Authors:** Abhimanu Pandey, Cheng Shen, Shouya Feng, Daniel Enosi Tuipulotu, Chinh Ngo, Cheng Liu, Melan Kurera, Anukriti Mathur, Shweta Venkataraman, Jing Zhang, Dipti Talaulikar, Renhua Song, Justin J.-L. Wong, Narci Teoh, Nadeem O. Kaakoush, Si Ming Man

**Affiliations:** ^1^ Division of Immunology and Infectious Disease, The John Curtin School of Medical Research, The Australian National University, Canberra, Australia.; ^2^ Conjoint Gastroenterology Laboratory, QIMR Berghofer Medical Research Institute, Herston, Australia.; ^3^ School of Medicine, University of Queensland, Herston, Australia.; ^4^ Mater Pathology, Mater Hospital, South Brisbane, Australia.; ^5^ Haematology Translational Research Unit, ACT Pathology, Canberra Health Services, Canberra, Australian Capital Territory, Australia.; ^6^ Department of Human Genomics, ACT Pathology, Canberra, Australian Capital Territory, Australia.; ^7^ School of Medicine and Psychology, College of Health and Medicine, The Australian National University, Canberra, Australia.; ^8^ Epigenetics and RNA Biology Program Centenary Institute, The University of Sydney, Camperdown 2050, Australia.; ^9^ Faculty of Medicine and Health, The University of Sydney, Camperdown 2050, Australia.; ^10^ Gastroenterology and Hepatology Unit, The Australian National University Medical School at The Canberra Hospital, The Australian National University, Canberra, Australia.; ^11^ School of Biomedical Sciences, University of New South Wales, Sydney, NSW, Australia.

## Abstract

The innate immune response contributes to the development or attenuation of acute and chronic diseases, including cancer. Microbial DNA and mislocalized DNA from damaged host cells can activate different host responses that shape disease outcomes. Here, we show that mice and humans lacking a single allele of the DNA repair protein Ku70 had increased susceptibility to the development of intestinal cancer. Mechanistically, Ku70 translocates from the nucleus into the cytoplasm where it binds to cytosolic DNA and interacts with the GTPase Ras and the kinase Raf, forming a tripartite protein complex and docking at Rab5^+^Rab7^+^ early-late endosomes. This Ku70-Ras-Raf signalosome activates the MEK-ERK pathways, leading to impaired activation of cell cycle proteins Cdc25A and CDK1, reducing cell proliferation and tumorigenesis. We also identified the domains of Ku70, Ras, and Raf involved in activating the Ku70 signaling pathway. Therapeutics targeting components of the Ku70 signalosome could improve the treatment outcomes in cancer.

## Teaser

Ku70 undergoes cytoplasmic translocation in response to DNA and forms a signalosome to attenuate the development of cancer.

## INTRODUCTION

Pattern recognition receptors are critical in the host defense against infectious disease and in the development of cancer and other chronic inflammatory conditions (*1*, *2*). DNA is the genetic code of life but also represents a form of microbial or damage-associated signal that can induce an immune response (*3*, *4*). Central to immune surveillance are the cytoplasmic DNA sensors that respond to microbial or endogenous DNA liberated from microbes or damaged and/or dying host cells. Detection of DNA by cytoplasmic innate immune sensors AIM2, cGAS, and STING leads to a robust immune response that can affect the outcome of inflammatory diseases and cancer (*5*–*15*).

Ku70 is an evolutionarily conserved protein that binds to double-stranded DNA breaks in the nucleus to facilitate DNA repair via the nonhomologous end joining pathway (*16*, *17*). In addition to the nucleus, Ku70 is found within the cytoplasm (*18*, *19*). Cytoplasmic Ku70 can induce the production of type III interferons (IFNs) in human and mouse cells in response to bacterial and viral DNA (*20*–*22*). Further, Ku70 can bind to the human bacterial pathogen *Rickettsia conorii* at the plasma membrane and facilitate the invasion of the bacterium into nonphagocytic mammalian cells (*23*). In addition to innate immune sensing of pathogens, Ku70 recognizes accumulated cytoplasmic DNA in CD4^+^ T cells and drives CD4^+^ T cell activation, promoting aging-related autoimmunity (*24*), further implicating a role for Ku70 beyond DNA damage repair. Genetic deletion of the gene encoding Ku70 in mice leads to the development of spontaneous T cell lymphoma and hepatocellular carcinoma (*25*, *26*). Mice lacking Ku70 carrying a hypomorphic p53 mutation (the p53^R172P^ mice) lead to early death, with a single mouse surviving until day 139 and exhibiting histological features of invasive colorectal cancer (*27*). While these findings hinted toward a putative role of Ku70 in cancer, mice lacking Ku70 suffer from poor growth and have a smaller body size (*25*–*28*), factors that would have likely confounded the interpretation of a cancer-associated role of Ku70.

Using mice lacking a single allele of the gene encoding Ku70 (*Ku70*^+/−^ mice), which do not have growth defects, we found that they lost more body weight during the development of colitis and intestinal cancer and had a greater tumor burden compared with littermate wild-type (WT) mice. The effects of Ku70 on attenuating colitis and intestinal cancer were independent of the role of Ku70 in DNA repair and the production of inflammatory markers and IFNs. Instead, Ku70 interacts with the guanosine triphosphatase (GTPase) Ras and the kinase Raf, forming a tripartite protein complex. This signalosome docks at the endosomal membrane and induces activation of the mitogen-activated protein kinase (MAPK) kinase (MEK)–extracellular signal–regulated kinase (ERK) signaling pathway during tumorigenesis, attenuating the development of intestinal cancer. Findings from this study may facilitate the design of therapeutics targeting components of the Ku70 signaling pathway, ultimately improving the treatment outcomes for individuals with inflammatory diseases and cancer.

## RESULTS

### Ku70 attenuates the development of colitis and intestinal cancer

**Fig. 1.**
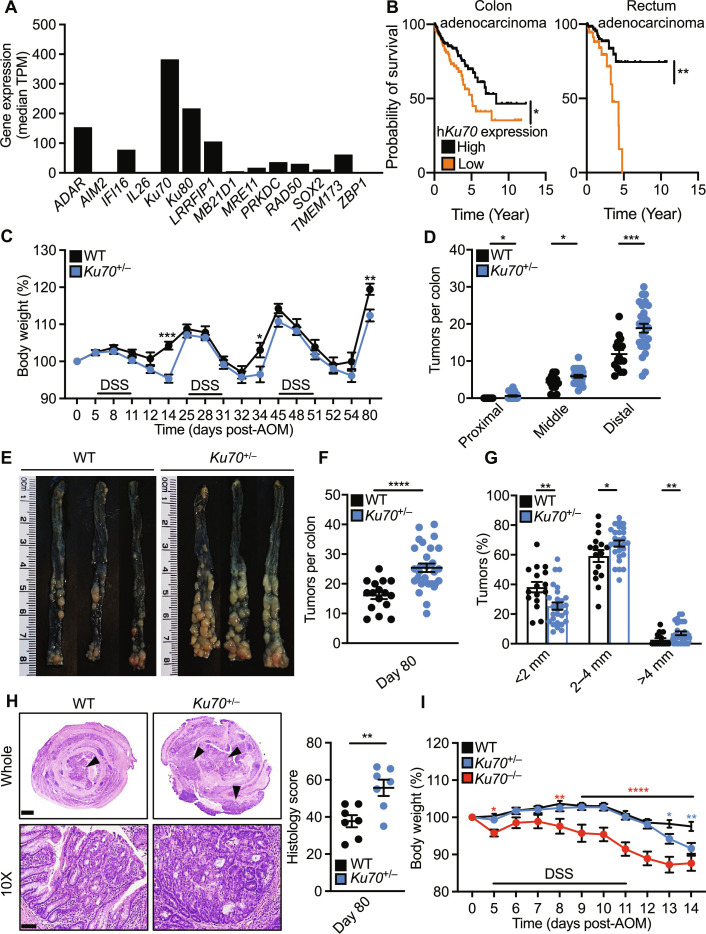
Ku70 attenuates the development of colitis and colitis-associated colorectal cancer. (**A**) The expression pattern of nucleic acid sensors in the colon tissue of healthy humans. Data are generated using the Genotype-Tissue Expression (GTEx) portal (*114*), and values are expressed as the median of transcripts per million (TPM). (**B**) Association between Ku70 expression and survival of patients with colon adenocarcinoma (left) and rectum adenocarcinoma (right). Data are generated using the Human Protein Atlas (*115*). (**C**) Percentage change in body weight of WT (*n* = 16) and *Ku70*^+/−^ (*n* = 28) mice on the 129 genetic background during AOM-DSS treatment. (**D**) Number of tumors in the indicated parts of the colon of mice 80 days after AOM injection. (**E**) Representative images of colon tumors in mice 80 days after AOM injection. (**F** and **G**) Total number (F) and size (G) of tumors as shown in (E). (**H**) Hematoxylin and eosin (H&E) staining of the colon tissue of mice 80 days after AOM injection (left), quantified by total histological scores (right). Scale bars, 1 mm (top) and 100 μm (bottom). (**I**) Percentage change in body weight of littermate WT (*n* = 25), *Ku70*^+/−^ (*n* = 20), and *Ku70*^−/−^ (*n* = 15) mice on the 129 genetic background during AOM-DSS treatment. Each symbol [(D) and (F) to (H)] or image (E) represents an individual mouse. Arrowheads indicate dysplasia or adenoma (H). *P < 0.05; **P < 0.01; ***P < 0.001; ****P < 0.0001 by log-rank (Mantel-Cox) test (B), two-way analysis of variance (ANOVA) with Holm-Šídák’s multiple comparisons test [(C) and (I)], or unpaired *t* test [(D) and (F) to (H)]. Data are pooled from three independent experiments in (C), (D), (F), (G), and (I), and two experiments in (H) and are presented as mean ± SEM in (C), (D), and (F) to (I).

Cytoplasmic DNA receptors contribute to intestinal homeostasis, and their expression profiles are differentially regulated during the development of colorectal cancer (*29*). A comparison of the expression of genes encoding cytosolic DNA sensors based on the Genotype-Tissue Expression database revealed that the gene encoding Ku70, *XRCC6*, is highly expressed in the human colon (Fig. 1A). Stratification of humans with colon or rectal adenocarcinoma revealed that those with a higher expression of Ku70 in the tumor tissue survived longer than those who had a lower expression of Ku70 (Fig. 1B). Furthermore, we observed that *XRCC6* is altered in multiple human cancers, with colon adenocarcinoma having the highest prevalence of alterations, defined by mutations, amplification, fusion, and loss of the gene (fig. S1, A and B). In addition, we examined the expression of the gene encoding Ku70 in samples from patients with colorectal cancer and inflammatory bowel disease called Crohn’s disease by analyzing the data from previous studies (figs. S2, A to F, and S3, A to C) (*30*, *31*). We found that the expression of the gene encoding Ku70 in the stromal and immune compartments of patients with colonic polyps and colorectal cancer decreased as compared to healthy individuals (fig. S2B). In the epithelial compartment, the expression of the gene encoding Ku70 in patients with colonic polyps decreased and in patients with colorectal cancer increased as compared to healthy individuals (fig. S2B). Further analysis of the stromal and epithelial compartments revealed that the expression of the gene encoding Ku70 tended to decrease in myofibroblasts, stem cells, enterocyte progenitors, and Best4^+^ enterocytes in patients with colonic polyps and colorectal cancer as compared to healthy individuals (fig. S2, C to F). In addition, we observed that the fraction of cells expressing the gene encoding Ku70 in patients with Crohn’s disease who have noninflamed or inflamed colons increased as compared to healthy individuals (fig. S3B). However, the average expression of the gene encoding Ku70 in cells from the stromal and epithelial compartments decreased (fig. S3B). Moreover, mice treated with the DNA-damaging agent azoxymethane (AOM) and inflammation-inducer dextran sulfate sodium (DSS) for 80 days had an increased trend of Ku70 expression in CD324^+^ (also called E-cadherin) epithelial cells and a decreased trend of the expression of Ku70 in CD45R^+^ nonepithelial (myeloid) cells in the tumor tissue compared to the nontumor tissue on day 80 (fig. S4, A and B). Together, these findings hint at the potential role of Ku70 during the development of intestinal inflammation and cancer.

To further elucidate whether Ku70 plays a role in intestinal cancer, we generated littermate WT, *Ku70*^+/−^, and *Ku70*^−/−^ mice (fig. S4, C to E). Similar to previous studies (*25*, *26*), *Ku70*^−/−^ mice on either the 129 or 129xC57BL/6N background were smaller in body size and weight compared with their corresponding WT littermate controls (fig. S4, C to E). However, *Ku70*^+/−^ mice, lacking a single allele of the gene encoding Ku70, were not growth retarded and had similar body weight and physical appearance compared with WT littermate mice (fig. S4, C to E), providing an opportunity to revisit the biological roles of Ku70 in cancer. Heterozygous deletion is the most common form of *XRCC6* alteration in tumors of patients with colon and rectal adenocarcinoma (fig. S5, A and B). We used a chemically induced colitis-associated colorectal cancer model and treated littermate WT, *Ku70*^+/−^, and *Ku70*^−/−^ mice with AOM and DSS (fig. S4A). We found that *Ku70*^+/−^ mice lost more body weight over three cycles of DSS and, on day 80 of the model, developed more tumors in the colon compared with WT mice (Fig. 1, C to F). *Ku70*^−/−^ mice did not survive the 80-day model. Tumors from *Ku70*^+/−^ mice were largely localized to the middle and distal colons and were larger in size compared to those of WT mice (Fig. 1, D, E, and G). *Ku70*^+/−^ mice also had increased dysplasia and extent of tissue damage than that of WT mice (Fig. 1H). The increased susceptibility of littermate *Ku70*^+/−^ mice to AOM and DSS was observed as early as day 14 of the model, with *Ku70*^+/−^ mice losing more body weight and having slightly shortened colon compared with WT controls (Fig. 1I and fig. S6A). On day 14, *Ku70*^−/−^ mice, which survived a single round of DSS, also lost substantially more body weight compared with WT controls (Fig. 1I). We also observed an increased susceptibility of *Ku70*^+/−^ mice and *Ku70*^−/−^ mice to DSS alone in the absence of AOM injection (fig. S6, B and C). In addition, we found that *Ku70*^+/−^ and *Ku70*^−/−^ mice on the 129 background backcrossed to the C57BL/6N background to rescue caspase-11 expression (fig. S6D) were also more susceptible to DSS-induced colitis compared with their WT littermate controls (fig. S6, E and F). The gut microbiome profile between littermate WT and *Ku70*^+/−^ mice was similar at both days 0 and 80 of the model (fig. S7, A to D), suggesting that the observed differences in tumor phenotype between WT and *Ku70*^+/−^ mice were not due to differences in their gut microbiota composition.

**Fig. 2.**
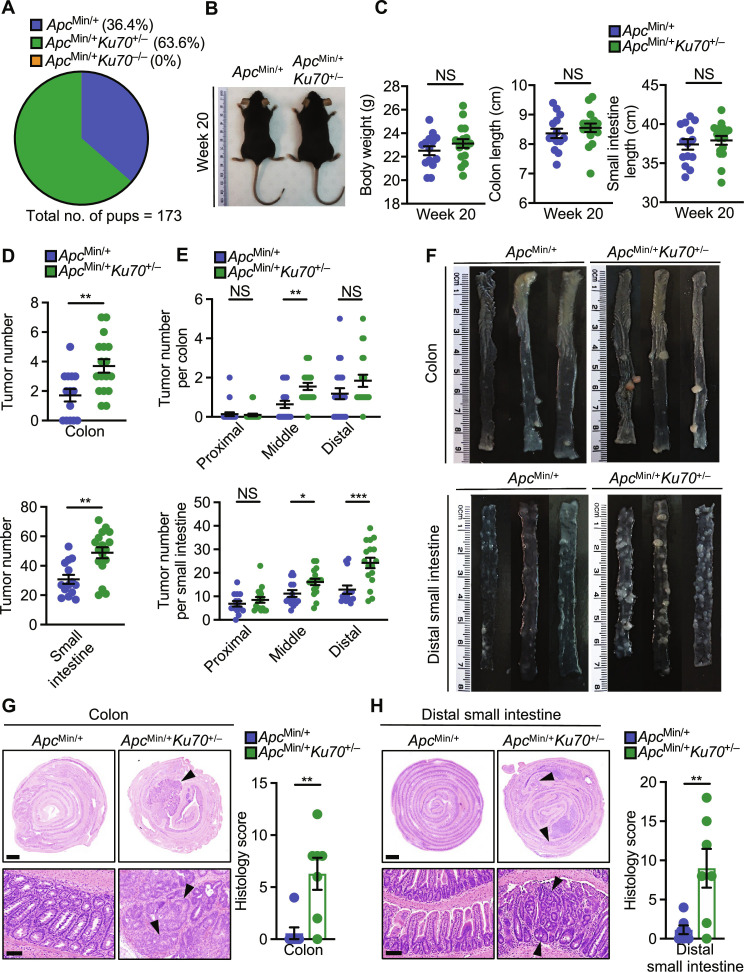
Ku70 prevents the development of spontaneous intestinal cancer. (**A**) Percentage of mice with the three indicated genotypes following the *Apc*^Min/+^ *Ku70*^+/−^ × *Apc*^+/+^ *Ku70*^+/−^ breeding scheme. (**B**) Representative image of littermate *Apc*^Min/+^ and *Apc*^Min/+^ *Ku70*^+/−^ mice on the 129xC57BL/6N genetic background at 20 weeks of age. (**C**) Body weight (left), colon length (middle), and small intestine length (right) of *Apc*^Min/+^ and *Apc*^Min/+^ *Ku70*^+/−^ mice at 20 weeks of age. (**D** to **F**) Tumor numbers in the whole colon (top) and whole small intestine (bottom) (D), tumor numbers in the indicated parts of the colon (top) and small intestine (bottom) (E), and representative images of the colon (top) and distal small intestinal (bottom) (F) of littermate *Apc*^Min/+^ and *Apc*^Min/+^*Ku70*^+/−^ mice at 20 weeks of age. (**G**) H&E staining (left) and histological scores (right) in the colon tissue of littermate *Apc*^Min/+^ and *Apc*^Min/+^*Ku70*^+/−^ mice at 20 weeks of age. Scale bars, 1 mm (top) and 100 μm (bottom). (**H**) H&E staining (left) and histological scores (right) in the distal small intestine tissue of littermate *Apc*^Min/+^ and *Apc*^Min/+^*Ku70*^+/−^ mice at 20 weeks of age. Scale bars, 1 mm (top) and 100 μm (bottom). Each symbol represents an individual mouse [(C) to (E) and (G) and (H)]. Arrowheads indicate dysplasia or adenoma (G and H). NS, not statistically significant, *P < 0.05; **P < 0.01; ***P < 0.001; by unpaired *t* test [(C) to (E), (G), and (H)]. Data are presented as means ± SEM in (C) to (E), (G), and (H).

In a second model of intestinal tumorigenesis, we crossed *Ku70*^+/−^ mice with mice carrying a heterozygous mutation in the gene encoding adenomatous polyposis coli (*Apc*^Min/+^ mice), which develop polyps spontaneously throughout the entire intestinal tract (*32*), similar to that in humans (*33*). Examination of the 173 pups born with the *Apc*^Min/+^ mutation revealed no *Apc*^Min/+^ *Ku70*^−/−^ mice (Fig. 2A), suggesting that the growth defects seen in *Ku70*^−/−^ mice compounded with the *Apc*^Min/+^ mutation might be lethal in utero. By contrast, *Apc*^Min/+^ *Ku70*^+/−^ mice were viable and had similar body weight and intestinal length compared with littermate *Apc*^Min/+^ *Ku70*^+/+^ mice (Fig. 2, B and C). Further, the gut microbiota composition was similar between *Apc*^Min/+^ *Ku70*^+/+^ mice and *Apc*^Min/+^ *Ku70*^+/−^ mice (fig. S7, E to H). *Apc*^Min/+^ *Ku70*^+/−^ mice developed, at 20 weeks of age, substantially more tumors in the colon and small intestine compared with *Apc*^Min/+^ *Ku70*^+/+^ mice (Fig. 2, D to F). Histopathological analyses revealed increased adenoma formation in the colon and distal small intestine of *Apc*^Min/+^ *Ku70*^+/−^ mice compared with *Apc*^Min/+^ *Ku70*^+/+^ mice (Fig. 2, G and H). The findings from *Apc*^Min/+^ *Ku70*^+/−^ mice faithfully recapitulated that of the *Ku70*^+/−^ mice used in the colitis-associated colorectal cancer model, with the two independent models of tumorigenesis indicating that the haploinsufficiency of Ku70 increases the susceptibility to intestinal cancer.

### Ku70 activates the ERK signaling pathway during the development of colitis

**Fig. 3.**
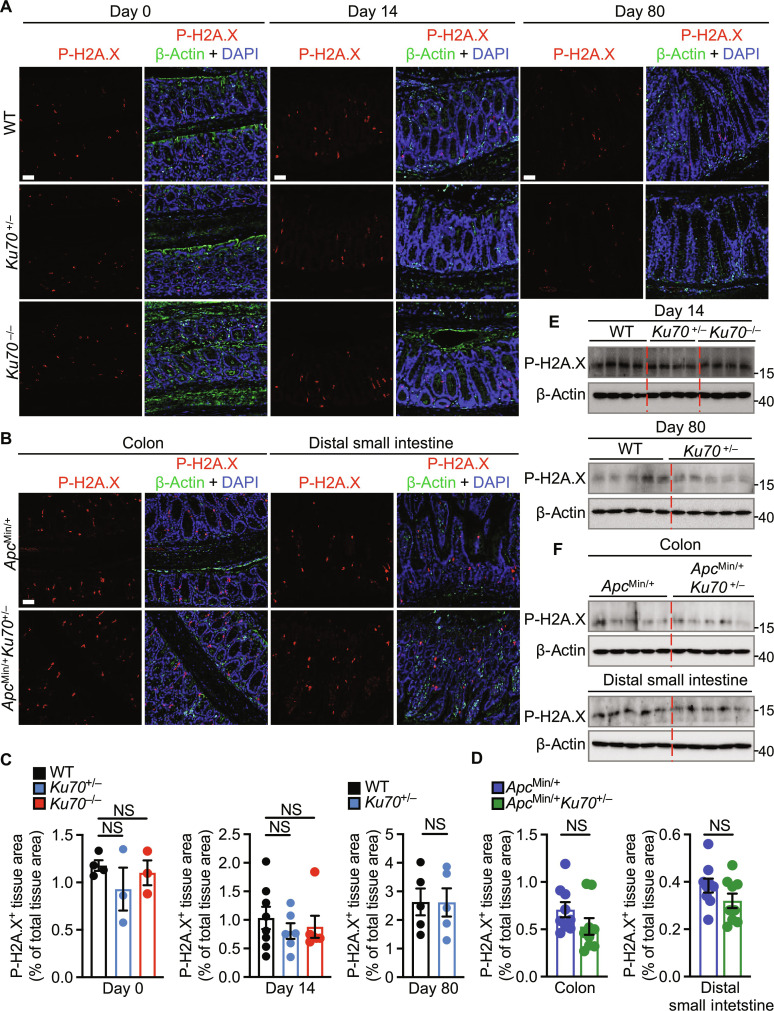
Ku70 does not affect DNA damage during the development of colitis. (**A** and **B**) Immunohistochemical staining of P-H2A.X, β-actin, and DAPI in the colon tissue of littermate WT, *Ku70*^+/−^, and *Ku70*^−/−^ mice on the 129 genetic background on day 0 (top left), 14 days (top middle), or 80 days after AOM-DSS treatment (right) (A) or in the colon tissue (bottom left) and distal small intestinal tissue (bottom right) of *Apc*^Min/+^ and *Apc*^Min/+^ *Ku70*^+/−^ mice (B). Scale bars, 50 μm. (**C** and **D**) Quantification of P-H2A.X–positive area over total colon tissue area as in (A) and (B). (**E** and **F**) Immunoblot of indicated protein in the colon tissue at day 14 (top) and day 80 (bottom) after AOM injection (E) or in the colon tissue (top) and distal small intestinal tissue (bottom) of *Apc*^Min/+^ and *Apc*^Min/+^ *Ku70*^+/−^ mice (F). Each symbol (C) or lane [(E) and (F)] represents an individual mouse. Each symbol represents one of the three (proximal, middle, or distal) parts of the colon (left) or one of three (outer, middle, or inner) parts of the distal small intestine (right) in (D). NS, not statistically significant by one-way ANOVA with Tukey’s multiple comparisons test [(C), left and middle] or unpaired *t* test [(C), right, and (D)]. Data are presented as means ± SEM in (C) and (D).

Given that Ku70 is a key player in the nonhomologous end joining pathway of DNA repair (*34*), we investigated whether the DNA repair function of Ku70 is required to attenuate tumorigenesis. Immunofluorescence staining of phosphorylated histone 2AX (P-H2A.X, also called γH2AX), a marker of double-stranded DNA breaks (*35*), revealed no difference in the colon of littermate WT, *Ku70*^+/−^, and *Ku70*^−/−^ mice at day 0 or day 14, or in the colon of WT and *Ku70*^+/−^ mice at day 80 of the AOM-DSS model, or in the colon and distal small intestine of *Apc*^Min/+^ *Ku70*^+/−^ and *Apc*^Min/+^ *Ku70*^+/+^ mice (Fig. 3, A to D). These observations were further confirmed by Western blot analysis (Fig. 3, E and F). Similarly, immunofluorescence staining of the marker of double-stranded DNA repair, 53BP1 (*36*), revealed no difference in the colon of littermate WT, *Ku70*^+/−^, and *Ku70*^−/−^ mice at day 0 or day 14, or in the colon of WT and *Ku70*^+/−^ mice at day 80, or in the colon and distal small intestine of *Apc*^Min/+^ *Ku70*^+/−^ and *Apc*^Min/+^ *Ku70*^+/+^ mice (fig. S8, A to D). Furthermore, we observed a similar expression of genes encoding DNA repair–associated kinases ataxia-telangiectasia mutated (ATM), checkpoint kinase 1 (CHK1), and DNA repair protein Rad51 among WT, *Ku70*^+/−^, and *Ku70*^−/−^ mice at days 0 and 14 (fig. S8E). These data suggest that Ku70 does not affect the nonhomologous end joining pathway of DNA repair during the development of intestinal inflammation and cancer.

**Fig. 4.**
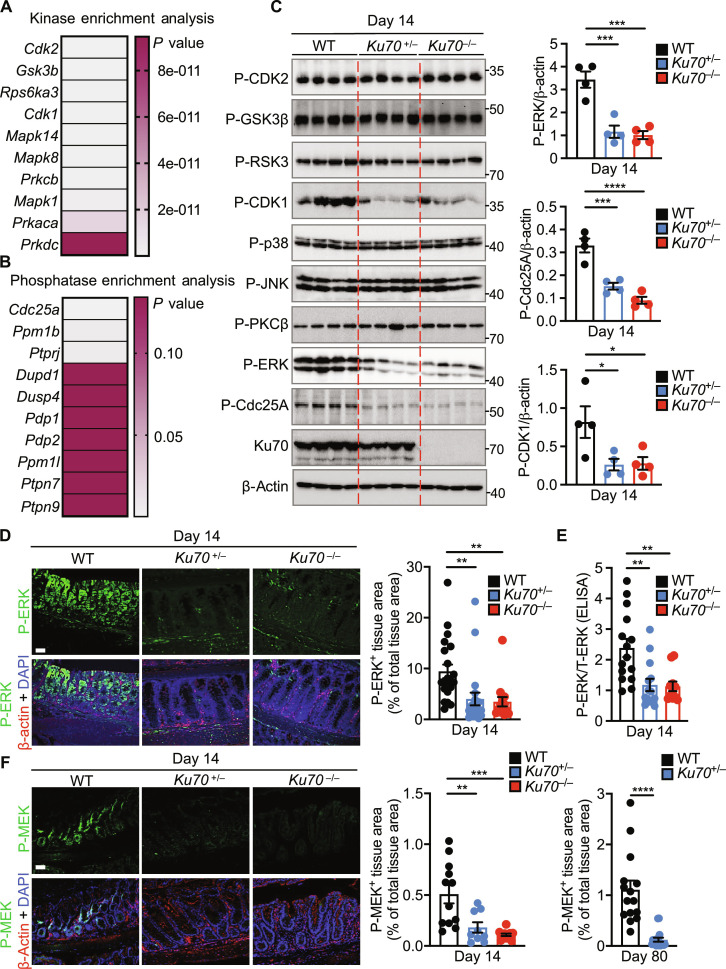
Ku70 activates the ERK signaling pathway. (**A**) Heatmap showing a list of genes encoding kinases filtered from a list of 509 differentially phosphorylated proteins obtained from the phospho-proteomic screen and analyzed using the Enrichr web server (*37*). (**B**) Heatmap showing a list of genes encoding phosphatases as analyzed in (A). (**C**) Immunoblot of the indicated proteins (left) and densitometric quantification (right) on the colon tissue lysate of littermate WT, *Ku70*^+/−^, and *Ku70*^−/−^ mice. (**D**) Immunohistochemical staining of P-ERK, β-actin, and DAPI (left) and quantification of P-ERK–positive area over total tissue area (right) in the colon tissue of mice. Scale bar, 50 μm. (**E**) Ratio of P-ERK over T-ERK by ELISA on the colon tissue lysate of mice. (**F**) Immunohistochemical staining of P-MEK, β-actin, and DAPI (left) and quantification of P-MEK–positive area over total tissue area in the colon tissue of littermate WT, *Ku70*^+/−^, and *Ku70*^−/−^ mice (middle) at day 14 and WT and *Ku70*^+/−^ mice (right) at day 80. Scale bars, 50 μm. Each lane (C) or symbol [(C) and (E)] represents an individual mouse. Each symbol represents one of the three (proximal, middle, or distal) parts of the mouse colon in (D) and (F). P- indicates phosphorylated protein in (C) and (D) to (F). T- indicates total protein in (E). *P < 0.05; **P < 0.01; ***P < 0.001; ****P < 0.0001; by one-way ANOVA with Tukey’s multiple comparisons test [(C) to (E) and (F), left] or unpaired *t* test [(F), right]. Data are representative of [(C), (D), and (F)] or pooled from (E) three independent experiments and are presented as means ± SEM in (C) to (F).

To determine how Ku70 differs from its classical role in DNA repair to prevent the development of tumors, we performed global phospho-proteomic mass spectrometry to identify differentially phosphorylated proteins, followed by kinase enrichment analysis and phosphatase enrichment analysis using the Enrichr web server (*37*). Analysis of the colonic tissue of littermate WT, *Ku70*^+/−^, and *Ku70*^−/−^ mice at day 14 of the AOM-DSS model identified top candidates that differentially phosphorylated or dephosphorylated proteins (Fig. 4, A and B; fig. S9; and table S1). The kinase enrichment analysis identified CDK2, GSK3β, RSK3, CDK1, p38, c-Jun N-terminal kinase (JNK), protein kinase Cβ (PKCβ), and ERK, which were further filtered by immunoblotting analysis (Fig. 4, A and C, and fig. S10A). Of these kinases, we validated a marked reduction in the phosphorylation of the kinase CDK1 (also known as Cdc2) and the MAPK ERK in the colon tissue of *Ku70*^+/−^ and *Ku70*^−/−^ mice compared to that of WT mice on day 14, but not on day 0 (Fig. 4, C to E, and fig. S10B). Phosphorylation of other MAPKs, JNK and p38, was not differentially altered between WT, *Ku70*^+/−^, and *Ku70*^−/−^ mice (Fig. 4C and fig. S10A). Reduced phosphorylation of ERK was also observed in the colon of WT and *Ku70*^+/−^ mice at day 80 and in the colon and distal small intestine of *Apc*^Min/+^ *Ku70*^+/−^ mice compared with *Apc*^Min/+^ *Ku70*^+/+^ mice (fig. S11, A to G).

The phosphorylation of ERK is driven by the kinase MEK (*38*). We found that phosphorylation and activation of MEK were indeed markedly reduced in the colon of *Ku70*^+/−^ and *Ku70*^−/−^ mice compared with WT mice (Fig. 4F and fig. S12, A and B). ERK inhibits the progression of the cell cycle by suppressing the phosphatase Cdc25A, which can associate with the kinase inhibitory protein 14-3-3 and dephosphorylates and activates CDK1 (*39*, *40*). In addition, ERK can control the activation of CHK2, which negatively controls cell growth during the development of tumorigenesis (*41*–*43*). Cdc25A was the top candidate identified from the phosphatase enrichment analysis, and immunoblotting analysis identified a reduction in the phosphorylation of Cdc25A in the colon tissue of *Ku70*^+/−^ and *Ku70*^−/−^ mice compared to that of WT mice on day 14, but not on day 0 (Fig. 4, B and C, and fig. S10B). In addition, we observed decreased phosphorylation of CHK2 but similar expression of phosphorylated ATM, an activator of CHK2 (*44*), in the colon of both *Ku70*^+/−^ and *Ku70*^−/−^ mice at day 14, and between WT and *Ku70*^+/−^ mice at day 80 (fig. S12, E to H).

We show that overexpression of Ku70 in human embryonic kidney (HEK) 293T cells induced the phosphorylation of MEK, ERK, Cdc25A, and CDK1, with these cells having an impaired ability to undergo cell proliferation (fig. S13, A to D). Further, we observed increased amounts of cellular proliferation markers, Ki67 and proliferating cell nuclear antigen (PCNA), in the colon of *Ku70*^+/−^ and *Ku70*^−/−^ mice as compared to WT mice (fig. S14, A and B). Increased levels of Ki67 and PCNA were also observed in the colon and distal small intestine of *Apc*^Min/+^ *Ku70*^+/−^ mice compared with *Apc*^Min/+^ *Ku70*^+/+^ mice (fig. S14, C and D). In addition, we found that the average size of organoids from *Apc*^Min/+^ *Ku70*^+/−^ mice increased markedly with enhanced expression of Ki67 compared to that of organoids from *Apc*^Min/+^ mice (fig. S15, A to G). Immunoblotting analysis revealed a reduction in the phosphorylation of ERK in organoids from *Apc*^Min/+^ *Ku70*^+/−^ mice compared with that in littermate *Apc*^Min/+^ mice (fig. S15H). These results suggest that Ku70-mediated ERK signaling may contribute to controlling intestinal stem cell proliferation.

Given that the ERK pathway is linked to inflammation (*45*), we used multiplex enzyme-linked immunosorbent assay (ELISA) and quantitative reverse transcription polymerase chain reaction (qRT-PCR) to check the expression of cytokines, chemokines, and inflammatory markers. These analyses revealed no major differences for 28 inflammatory mediators in the colon tissue of WT, *Ku70*^+/−^, and *Ku70*^−/−^ mice at days 0 and 14 (figs. S16 and S17, A to C). Ku70 contributes to antiviral immunity by potentiating signaling through cytosolic DNA sensors cGAS and STING (*22*, *46*). Given that cGAS and STING contribute to the development of intestinal inflammation and cancer by restricting the phosphorylation of signal transducer and activator of transcription 3 (STAT3) and activation of nuclear factor κB (NF-κB), respectively (*8*, *9*), we investigated the role of Ku70 in STAT3 and NF-κB signaling. Immunoblotting revealed similar levels of phosphorylation of STAT3 and Iκβα in the colon tissue of WT, *Ku70*^+/−^, and *Ku70*^−/−^ mice at day 14 (fig. S17D). In addition, STING can induce the production of type I IFNs and the cytokines interleukin-6 (IL-6) and keratinocyte chemoattractant (KC, also known as CXCL1) (*9*). However, we did not observe differences among WT, *Ku70*^+/−^, and *Ku70*^−/−^ mice in the production of type I IFNs and the cytokines IL-6 and KC following the treatment of AOM and DSS (figs. S16 and S17C), suggesting that Ku70 may not require cGAS-STING–mediated signaling during the development of colitis.

### Ku70 does not affect immune cell infiltration and gut permeability during the development of colitis and intestinal cancer

Studies in mice have shown that homozygous deletion of the gene encoding Ku70 results in an increased incidence of spontaneous T cell lymphoma and decreased survival as compared to WT mice (*25*, *26*) and that Ku70 detects cytoplasmic DNA in human Jurkat T lymphocytes and aged primary mouse CD4^+^ T cells, leading to the activation and proliferation of these cells driving age-related autoimmunity (*24*). Therefore, we investigated whether the lack of Ku70 affects CD4^+^ and CD8^+^ T cells during the development of intestinal inflammation and cancer. Immunohistochemical staining revealed similar numbers of CD4^+^ and CD8^+^ cells in the colon tissue of littermate WT, *Ku70*^+/−^, and *Ku70*^−/−^ mice at days 0 and 14 and in the colon tissue of WT and *Ku70*^+/−^ mice at day 80, and in the colon and distal small intestine of *Apc*^Min/+^ *Ku70*^+/−^ and *Apc*^Min/+^ *Ku70*^+/+^ mice (fig. S18, A to E). These results may suggest that Ku70 does not affect the number of CD4^+^ and CD8^+^ cells during the development of colitis and intestinal cancer. We also observed a similar number of Ly6G^+^ neutrophils, FoxP3^+^ T regulatory cells, and F4/80^+^ macrophages in the colon tissue of littermate WT, *Ku70*^+/−^, and *Ku70*^−/−^ mice at day 0 or day 14. Furthermore, we tested whether Ku70 affects gut permeability during the development of colitis. We observed a similar expression of the tight junction marker claudin 2 and occludin in the colon tissue of WT, *Ku70*^+/−^, and *Ku70*^−/−^ mice at day 0 or day 14 (fig. S19, A to C). These data suggest that Ku70 may not contribute to DSS-induced barrier defects.

### Ku70 translocates to the cytoplasm and interacts with Ras and Raf to form a tripartite signalosome during the development of colitis

**Fig. 5.**
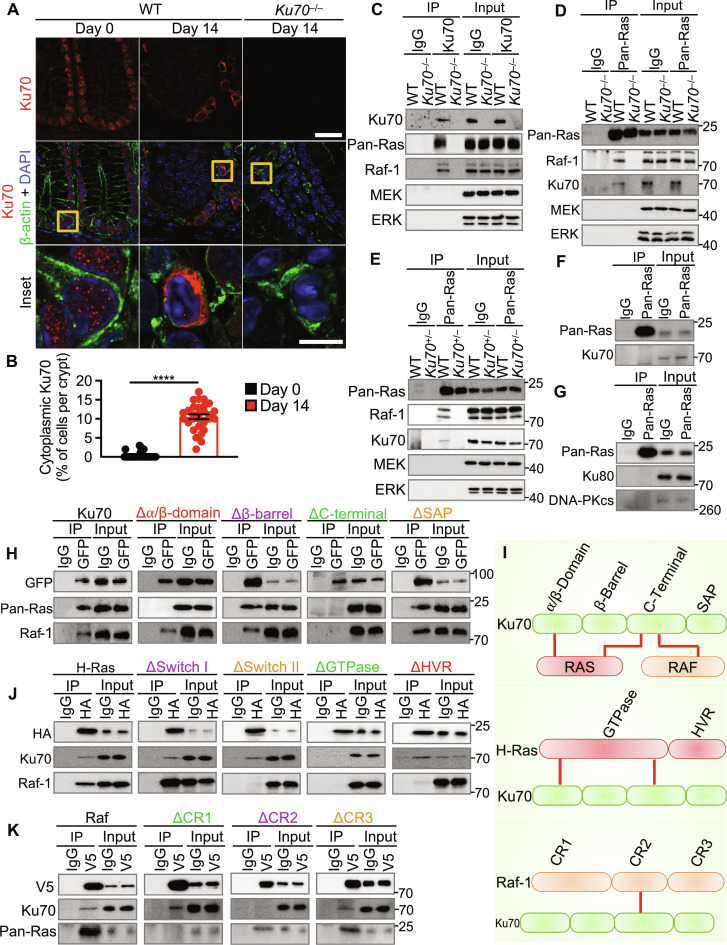
Ku70 translocates from the nucleus to cytoplasm and interacts with the GTPase Ras and the kinase Raf. (**A** and **B**) Immunohistochemical staining of Ku70, β-actin, and DAPI (A) and percentage of cells with cytoplasmic Ku70 (B) in the colon tissue of WT and *Ku70*^−/−^ mice untreated (day 0) or treated with AOM-DSS (day 14). Scale bars, 20 μm (top) and 5 μm (inset). (**C**) Immunoprecipitation (IP) of control (IgG) or Ku70 from the colon tissue lysate of WT and *Ku70*^−/−^ mice at day 14 and immunoblot of the indicated proteins. (**D** and **E**) IP of control (IgG) or pan-Ras from the colon tissue lysate of WT and *Ku70*^−/−^ mice (D) or WT and *Ku70*^+/−^ (E) mice at day 14 and immunoblot of indicated proteins. (**F**) IP of control (IgG) or Ras from the colon tissue lysate of untreated WT mice and immunoblot of the indicated proteins. (**G**) IP of control (IgG) or pan-Ras from the colon tissue lysate of WT mice at day 14 and immunoblot of the indicated proteins. (**H**) IP for control (IgG) or GFP-tagged Ku70 and immunoblot for the indicated proteins in HEK293T cells. (**I**) Model of Ku70-Ras-Raf interaction (top), Ku70-Ras interaction (middle), and Ku70–Raf-1 interaction (bottom). Red lines indicate interaction in (I). (**J**) IP for control (IgG) or HA-tagged H-Ras and immunoblot for the indicated proteins in HEK293T cells. (**K**) IP for control (IgG) or V5-tagged Raf and immunoblot for the indicated proteins in HEK293T cells. ****P < 0.0001; by unpaired *t* test (B). Data representative of three experiments in (A) to (K) and are presented as means ± SEM in (B).

Given that Ku70 is found in both the nucleus and the cytoplasm (*20*, *22*, *24*), we tested whether Ku70 migrates from the nucleus to the cytoplasm following DSS treatment to activate the ERK signaling pathway. We observed that Ku70 is localized to the nucleus of cells lining the intestinal epithelium within the colon tissue of untreated mice (Fig. 5, A and B, and fig. S20A). However, during the onset of colitis, we observed a marked redistribution of Ku70 to the cytoplasm (Fig. 5, A and B, and fig. S20A). The activation of MEK and ERK requires two upstream molecules, the small guanine-nucleotide binding protein Ras and the kinase Raf (*38*). We found a substantial tendency of co-occurrence in mutations between the gene encoding Ku70 and genes encoding ARAF, BRAF, and RAF1 (table S2). In addition, mutations between the gene encoding Ku70 and genes encoding HRAS and NRAS show co-occurrence, although not statistically significant, whereas mutations between the gene encoding KRAS are mutually exclusive (table S2).

To identify how Ku70 activates the MEK-ERK signaling pathway, we pulled down endogenous Ku70 from the colonic lysate in search of potential binding partner/s. Ku70 did not interact with MEK and ERK but, instead, interacted with two upstream proteins of the MEK-ERK pathway, Ras and Raf (Fig. 5, C to E). On the basis of this observation, Ku70 might form a complex with Ras or Raf individually or as a tripartite Ku70-Ras-Raf complex. To investigate these possibilities, we immunoprecipitated endogenous Ras in WT, *Ku70*^+/−^, and *Ku70*^−/−^ colon tissue lysates and blotted for Raf. We observed an interaction between Ras and Raf in WT colonic lysates, but not in *Ku70*^+/−^ and *Ku70*^−/−^ colonic lysates (Fig. 5, C to E). Together, these findings suggest that Ku70 is essential in mediating Ras-Raf interaction. These results also suggest that endogenous Ku70 interacts with Ras and Raf to form a tripartite complex and that a slight reduction in the bioavailability of Ku70 in *Ku70*^+/−^ mice is sufficient to compromise the interaction between Ku70 and Ras, perhaps due to the transient nature of this interaction. In addition, immunohistochemical staining revealed a substantial colocalization between Ras and Raf-1 in the colon tissue of WT mice as compared to *Ku70*^−/−^ mice (fig. S20B). The interaction between Ku70 and Ras was detected in colonic lysates from mice treated with AOM and DSS, but not in untreated mice (Fig. 5F), indicating that the Ku70-Ras-Raf complex does not form during homeostasis.

Ku70 initiates the repair of double-stranded DNA breaks by forming a complex with closely related protein Ku80 and DNA-dependent protein kinase catalytic subunit (DNA-PKcs) (*47*). In addition, previous studies have shown that the lack of Ku80 suppresses intestinal tumorigenesis, whereas the lack of DNA-PKcs leads to intestinal dysplasia in mice (*48*, *49*). Therefore, we tested whether Ku80 and DNA-PKcs are part of the Ku70-Ras-Raf complex. We immunoprecipitated endogenous Ras and immunoblotted for Ku80 and DNA-PKcs. We found that Ras did not interact with Ku80 or DNA-PKcs (Fig. 5G), suggesting that these molecules are not part of the Ku70-Ras-Raf complex.

To identify the specific domain/s of Ku70 that are required for mediating interaction with Ras and Raf, we generated and expressed plasmids encoding full-length Ku70 or Ku70 lacking one of the four domains: the α/β domain (Δα/β domain), β-barrel domain (Δβ-barrel domain), C-terminal domain (ΔC-terminal domain), or SAP domain (ΔSAP domain; named after proteins SAF-A/B, Acinus, and PIAS, containing the same motif) (fig. S20C). We found that the Δα/β domain Ku70 mutant and the ΔC-terminal domain Ku70 mutant had an impaired ability to interact with Ras (Fig. 5H). Further, the ΔC-terminal domain Ku70 mutant did not interact with Raf (Fig. 5H). These results suggest that the α/β domain and C-terminal domain of Ku70 are essential for interaction with Ras, whereas the C-terminal domain of Ku70 is required for interaction with Raf (Fig. 5I).

We performed reciprocal immunoprecipitation assays to identify the domain/s of Ras and Raf that are required for interaction with Ku70. To this end, we immunoprecipitated either full-length H-Ras or H-Ras lacking one of its two domains: the GTPase domain (ΔGTPase) or the hypervariable region/domain (ΔHVR) (Fig. 5J and fig. S20C). We found that the ΔGTPase domain of H-Ras had an impaired ability to interact with Ku70 (Fig. 5J). Within the GTPase domain of H-Ras, the switch I and switch II regions facilitate protein-protein interaction between Ras and Raf (*50*). ΔSwitch I and Δswitch II interacted with endogenous Ku70 (Fig. 5J), suggesting that Ku70 binds to H-Ras via region/s within the GTPase domain other than the switch I or switch II region. Immunoprecipitation of either full-length Raf or Raf lacking the conserved domain (CR) 1 (ΔCR1), CR2 domain (ΔCR2), or CR3 domain (ΔCR3) revealed that the ΔCR2 mutant of Raf did not interact with Ku70 (Fig. 5K and fig. S20C). These results collectively suggest that the GTPase domain of Ras and the CR2 domain of Raf mediate interaction with Ku70 (Fig. 5I).

Given that Ku70 binds to the GTPase domain of H-Ras, which is required for the conversion between the active and inactive state of Ras through guanosine diphosphate (GDP) and guanosine triphosphate (GTP) exchange (*50*), we tested whether Ku70 directly activates Ras. We first validated our assay by treating the cell lysate of untransfected HEK293T cells with GTPγS, which binds to the active form of Ras (positive control), or GDP, which binds to the inactive form of Ras (negative control). Our data showed increased levels of active Ras in the presence of GTPγS compared to that of GDP (fig. S20D). The cell lysates of HEK293T cells expressing the Ku70 plasmid had similar levels of active Ras compared with the empty vector (fig. S20D), indicating that Ku70 does not directly contribute to the activation of Ras. We next tested whether treatment with dabrafenib, which inhibits activation of Raf (fig. S20E), could affect the stability of the Ku70-Ras-Raf complex. To this end, we overexpressed green fluorescent protein (GFP)–tagged Ku70 in HEK293T cells and left these cells untreated or treated with dabrafenib. We found that dabrafenib treatment slightly impaired the interaction between Ku70 and Ras (fig. S20F). Further, we pulled down endogenous Raf-1 in HEK293T cells overexpressing GFP-tagged Ku70, which had been left untreated or treated with dabrafenib. We observed that dabrafenib treatment slightly impaired the interaction between Raf-1 and Ras, but not with Ku70 (fig. S20G). Together, these results indicate that dabrafenib-mediated inhibition of Raf might reduce the binding strength between Ras with Raf, and between Ku70 and Ras, which in turn might affect the signaling of the Ku70-Ras-Raf complex. Previous studies have shown that MEK inhibitors reduce cell proliferation but increase the expression of genes such as *Ctnnb1*, *Axin2*, and *Lgr5*, which are highly associated with cancer development (*51*, *52*). Organoids cultured from *Apc*^Min/+^ mice had increased expression of *Ctnnb1*, *Axin2*, and *Lgr5* following treatment with the MEK inhibitor U0126 as compared to the vehicle control (fig. S20H), consistent with previous studies (*51*, *52*).

### Ku70 senses cytosolic DNA and forms a signalosome at the endosome

**Fig. 6.**
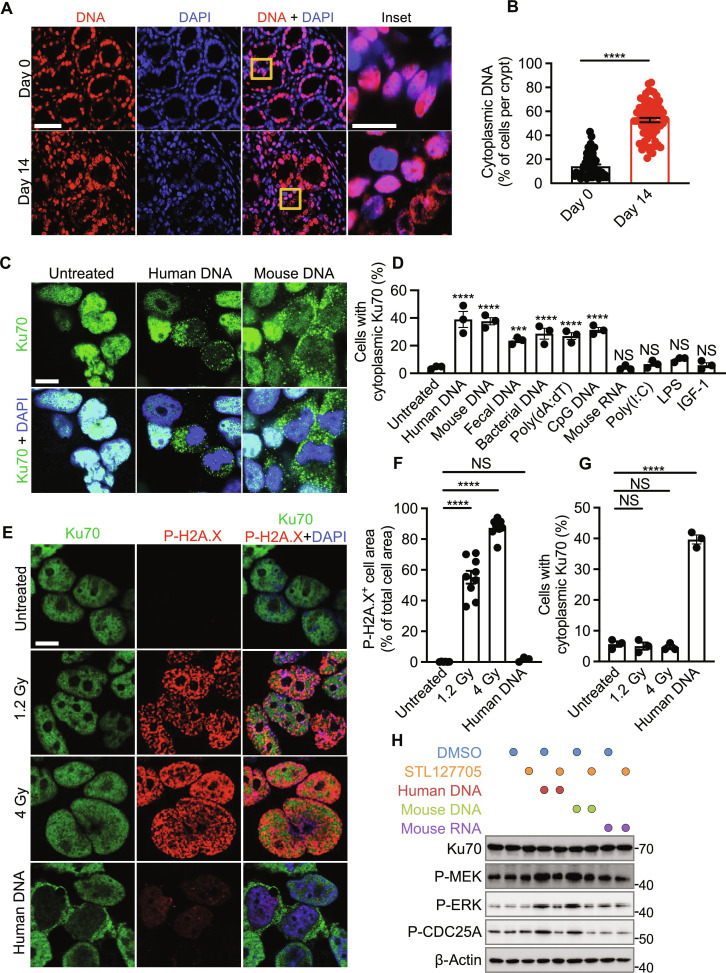
Ku70 translocates from the nucleus and senses cytosolic DNA. (**A**) Immunohistochemical staining of DNA and DAPI in the colon tissue of WT mice untreated (day 0) or treated with AOM-DSS (day 14). Scale bars, 20 μm (left) and 5 μm (inset). (**B**) Percentage of cells with cytoplasmic DNA in the colon tissue of WT mice as shown in (A). (**C**) Immunofluorescence staining of Ku70 and DAPI in HEK293T cells left nontransfected or transfected with human DNA (1 μg) and mouse colonic DNA (1 μg). Scale bar, 10 μm. (**D**) Percentage of cytoplasmic Ku70 in HEK293T cells left nontransfected or transfected for 6 hours with human DNA (1 μg), mouse colonic DNA (1 μg), mouse fecal DNA (1 μg), bacterial DNA (1 μg), poly(dA:dT) (1 μg), CpG DNA (1 μg), mouse colonic RNA (1 μg), and poly(I:C) (1 μg) or stimulated with LPS (1 μg) or IGF-1 (50 ng). (**E** to **G**) Immunofluorescence staining of Ku70, P-H2A.X, and DAPI (E), quantification of P-H2A.X–positive area over total cell area (F), and percentage of cytoplasmic Ku70 (G) in HEK293T cells left untreated or radiated with indicated doses or transfected with human DNA (1 μg). Scale bar, 10 μm. (**H**) Immunoblot of the indicated proteins on the cell lysate of HEK293T left untreated or pretreated with STL127705 (25 μM) for 1 hour. Cells were then left untreated or treated as indicated followed by incubation for 6 hours. DMSO was used to dissolve STL127705. NS, not statistically significant; ***P < 0.001; ****P < 0.0001; by unpaired *t* test (B) or one-way ANOVA with Tukey’s multiple comparisons test [(D), (F), and (G)]. Data representative of three mice in (A) and three experiments in (C), (D), and (H) or two experiments in (E) to (G). Data are presented as means ± SEM in (B), (D), (F), and (G).

Previous studies have shown that patients with ulcerative colitis have more nuclear- and mitochondria-free (cell-free) DNA in the plasma than healthy controls (*53*, *54*). In addition, mice treated with DSS have more cell-free DNA in the plasma than untreated control mice (*53*, *55*), suggesting the presence of mislocalized DNA during the development of colitis. We observed a marked increase in the staining of DNA in the cytoplasm of intestinal cells of mice with colitis than in the colon tissue of untreated mice (Fig. 6, A and B). Given that Ku70 translocated from the nucleus to the cytoplasm within the colon tissue of mice with colitis (Fig. 5, A and B, and fig. S20A), we hypothesized that cytoplasmic DNA could serve as an activator of Ku70-mediated ERK signaling.

To identify the ligand/s that induce the translocation of Ku70 from the nucleus to the cytoplasm, we transfected human DNA, mouse DNA, mouse fecal DNA, bacterial DNA, poly(deoxyadenylic-deoxythymidylic) acid sodium salt [poly(dA:dT)], CpG DNA, mouse RNA, and polyinosinic:polycytidylic acid [poly(I:C)] into HEK293T cells, or stimulated these cells with insulin growth factor 1 (IGF-1) or lipopolysaccharide (LPS). We observed that only DNA induced the translocation of Ku70 from the nucleus into the cytoplasm (Fig. 6, C and D, and fig. S21A). Irradiation did not induce the translocation of Ku70 from the nucleus to the cytoplasm within HEK293T cells but increased the amount of P-H2A.X, indicating double-stranded DNA breaks (Fig. 6, E to G). These data suggest that Ku70 remains localized to the nucleus following irradiation-induced double-stranded DNA damage, but specifically undergoes cytoplasmic translocation in response to cytoplasmic DNA. We also observed that transfection of DNA into primary fibroblasts from WT mice indeed triggered the production of IFN-λ, the production of which was reduced in both *Ku70*^+/−^ and *Ku70*^−/−^ fibroblasts (fig. S21B), consistent with previous studies (*20*–*22*). We next asked whether binding between cytosolic DNA and Ku70 induces the activation of ERK signaling. The small-molecule compound STL127705, which abolishes the interaction between DNA and Ku70 (*24*), reduced the phosphorylation of MEK, ERK, and CDC25A in HEK293T cells treated with human or mouse DNA (Fig. 6H). These results suggest that cytosolic DNA triggers the translocation of Ku70 into the cytoplasm and activates the MEK-ERK signaling pathway.

Mutations in the gene encoding KRAS and BRAF are prevalent in tumors of patients with colorectal cancer (*50*, *56*). The oncogenic mutations in KRAS most commonly occur at residues G12 and G13, whereas the most common mutations in BRAF occur because of valine–to–glutamic acid substitution (V600E) (*57*). To test whether these clinically important mutations affect Ku70-mediated MEK-ERK signaling, we used the HEK293T cell line that is nonmalignant (*58*), and WT for KRAS and BRAF, the HCT116 cell line that harbors a mutation in the gene encoding KRAS at codon 13 (G>A mutation corresponding to p.G13D) but carries WT BRAF (*51*, *59*), and the HT29 cell line that harbors mutations in the gene encoding BRAF at codons 119 (C>G mutation corresponding to p.T119S) and 600 (T>A mutations corresponding to p.V600E) but carries WT KRAS (*51*, *59*). We confirmed mutations in these cells using Sanger sequencing (fig. S22A). We observed that the overexpression of Ku70 in HEK293T cells induced the phosphorylation of MEK and ERK as expected (figs. S13B and S22B), whereas overexpression of Ku70 in HCT116 and HT29 cells did not affect the phosphorylation of MEK or ERK (fig. S22B). These results suggest that cells carrying mutations in genes encoding KRAS or BRAF might bypass the Ku70-mediated ERK signaling pathway.

**Fig. 7.**
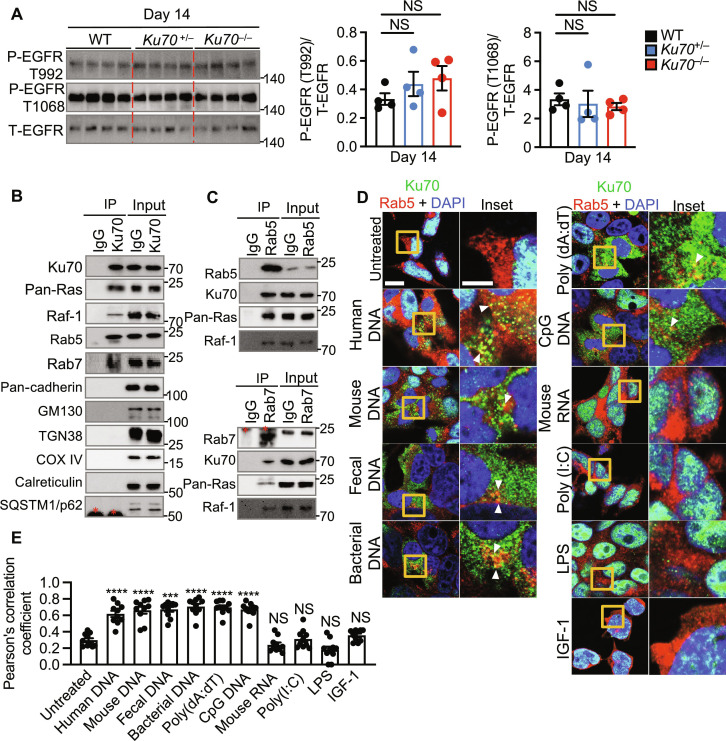
Cytoplasmic translocation of Ku70 activates the Ras-ERK signaling at the endosome. (**A**) Immunoblot of the indicated proteins (left) from the colon tissue lysate of littermate WT, *Ku70*^+/−^, and *Ku70*^−/−^ mice and the corresponding densitometric quantification (right). (**B**) IP of control (IgG) or Ku70 from the colon tissue lysate of WT mice at day 14 after AOM injection and immunoblot of the indicated proteins. (**C**) IP of control (IgG) or Rab5 (top) and control (IgG) or Rab7 (bottom) from the colon tissue lysate of WT mice at day 14 after AOM injection and immunoblot of the indicated proteins. (**D** and **E**) Immunofluorescence staining of Ku70, Rab5, and DAPI (D) and frequency of colocalization between Ku70 and Rab5 (E) in HEK293T cells left nontransfected or transfected for 6 hours with human DNA (1 μg), mouse colonic DNA (1 μg), mouse fecal DNA (1 μg), bacterial DNA (1 μg), poly(dA:dT) (1 μg), CpG DNA (1 μg), mouse colonic RNA (1 μg), and poly(I:C) (1 μg) or stimulated with LPS (1 μg) or IGF-1 (50 ng). Scale bars, 10 μm (left) and 2.5 μm (inset). Each symbol represents an individual mouse (A) or cell in (D). P- indicates phosphorylated protein, and T- indicates total protein (A). *Indicates IgG bands in [(B) and (C)]. NS, not statistically significant; ***P < 0.001; ****P < 0.0001; by one-way ANOVA with Tukey’s multiple comparisons test [(A) and (D)]. Data representative of three experiments in (A), (D), and (E) or three mice in (B) and (C). Data are presented as mean ± SEM in (A) and (E).

The Ras-ERK signaling pathway activated by growth factors, such as epidermal growth factor (EGF) and IGF, occurs largely at the plasma membrane through the EGF receptor (EGFR) (*38*). Therefore, we tested the activation of EGFR in the colon tissue of WT, *Ku70*^+/−^, and *Ku70*^−/−^ at day 14. We found that the lack of Ku70 does not affect the phosphorylation of EGFR (Fig. 7A). In addition to the plasma membrane, Ras-ERK signaling has been observed at endosomes, the endoplasmic reticulum, the Golgi apparatus, and the mitochondria (*60*, *61*). Coimmunoprecipitation revealed that endogenous Ku70 from the colon tissue lysate of WT mice interacted with the early endosome marker Rab5 and the late endosome marker Rab7 (Fig. 7B). However, we did not observe an interaction between Ku70 and markers of the plasma membrane (pan-cadherin), Golgi (GM-130), trans-Golgi network (TGN38), mitochondria (COX IV), endoplasmic reticulum (calreticulin), and macroautophagy vesicles (SQSTM1/p62) (Fig. 7B). Reciprocal immunoprecipitation of endogenous Rab5 and Rab7 confirmed their interaction with Ku70 (Fig. 7C) and that they also interacted with endogenous Ras and Raf (Fig. 7, B and C). Further, cytoplasmic Ku70 colocalized with Rab5 in the cytoplasm of HEK293T cells stimulated with human DNA, mouse DNA, mouse fecal DNA, bacterial DNA, poly(dA:dT), and CpG DNA, but not with mouse RNA, poly(I:C), IGF-1, or LPS (Fig. 7, D and E). These results suggest that following the sensing of cytosolic DNA, Ku70 assembles into a Ku70-Ras-Raf signalosome at the endosome to trigger activation of the MEK-ERK signaling pathway, which may attenuate the development of intestinal cancer.

## DISCUSSION

Activation of cytosolic DNA sensors can instigate signaling cascades that modulate the outcome of multiple diseases, including cancer (*3*). We observed that the decreased expression and mutations of the gene encoding the cytosolic DNA sensor Ku70 are associated with the development of colorectal cancer in humans and that mice lacking one copy of the gene encoding Ku70 are more susceptible to colitis, colitis-associated colorectal cancer, and spontaneous intestinal cancer. Previous studies have shown that the expression of Ku70 is down-regulated in the tumor tissue of patients with colorectal cancer compared to adjacent tissues (*62*–*68*). In addition, the activation of ERK and MEK is decreased in tumor tissues compared to paired normal tissues from patients with colorectal cancer (*69*–*73*). Mutations in genes encoding three isoforms of Ras and Raf have been found in most tumor samples from patients with colorectal cancer (*74*–*76*). Further, the activation of ERK has been shown to drive tumor formation in *Apc*^Min/+^ mice by the Toll-like receptor adaptor protein myeloid differentiation factor 88 (MyD88) (*77*). We observed a substantial tendency of co-occurrence in mutations between the gene encoding Ku70 and those in genes encoding ARAF, BRAF, and RAF1. In addition, mutations in the gene encoding Ku70 and genes encoding HRAS and NRAS show co-occurrence. Furthermore, overt activation of Ras-ERK signaling plays a critical role in the progression of colorectal cancer (*78*). Our study suggests that the Ku70-ERK signaling pathway is tumor suppressive, which is in contrast to the observation that Ras/Raf mutations, which are common in colorectal cancer, drive aberrant activation of downstream ERK-MAPK signaling. However, in colon cancer, activation of ERK can occur in a cell type–specific manner (*79*). We observed that the expression of the gene encoding Ku70 is decreased in certain cell types of the epithelial and stromal compartments from the patients with Crohn’s disease and colorectal cancer, suggesting that Ku70 might function in a cell type–specific manner during the development of intestinal inflammation and cancer. Moreover, the activation of ERK tends to decrease as the stages of colorectal cancer progress (*71*). Further studies are required to elucidate which cell types undergo Ras-ERK signaling for the progression of colorectal cancer and which cell types undergo Ku70 signaling for the attenuating of colorectal cancer. Moreover, Cdc25A is frequently found in many cancers, including in up to 53% of tumor samples from patients with colorectal cancer (*80*), suggesting that overexpression of Cdc25A might be associated with the development of colorectal cancer. Similarly, the expression of the gene encoding CDK1 is increased in tumor samples from patients with colorectal cancer compared to healthy controls (*81*), and the overexpression of CDK1 is associated with decreased overall survival of patients with colorectal cancer (*82*). Together, the expression of the components of the Ku70-mediated Ras-ERK-Cdc25A-CDK1 pathway could serve as a potential biomarker in predicting the survival and prognosis of patients with intestinal cancer.

Our findings show that Ku70 forms a cytosolic signalosome composed of Ku70, Ras, and Raf that docks at the endosomal membrane. This complex mediates the activation of the MEK-ERK-Cdc25A-CDK1 signaling axis leading to an antitumorigenic effect. Furthermore, activation of the Ras-ERK pathway protects mice against colitis (*83*) and inhibits mammalian cell proliferation (*81*, *84*, *85*). Conditional deletion of MEK1 in intestinal epithelial cells and genetic deficiency of MEK2 result in severe intestinal defects, including distorted crypts, blunted and shortened villi, and hyperplasia of goblet cells (*86*), suggesting that both isoforms of MEK are involved in maintaining intestinal homeostasis. Genetic deficiency in the isoform ERK2 in mice results in embryonic lethality, whereas genetic deficiency in ERK1 does not (*87*). However, it is unclear which ERK isoform is important for intestinal homeostasis (*86*). Our data also suggest that Ku70-mediated MEK-ERK signaling might restrict the proliferation of cells that are not malignant, such as HEK293T cells (*58*). Expression of plasmids encoding constitutively active MEK1 or MEK2 drives sustained phosphorylation of ERK1 or ERK2, which, in turn, restricts the proliferation of human hepatocellular carcinoma cell line Huh-7 (*81*) and human normal intestinal epithelial crypt cell cultures (*85*). However, genetic silencing of the gene encoding MEK2 using short hairpin RNAs inhibits the proliferation of human colon carcinoma cell lines HCT116 and HT29, which harbor mutations in KRAS and BRAF (*88*). Similarly, we show that cells carrying mutations in genes encoding KRAS and BRAF could bypass the Ku70-mediated ERK signaling pathway, suggesting that Ku70-mediated MEK-ERK signaling might be beneficial in cells that are not malignant and do not harbor mutations in genes encoding Ras and Raf. Together, these findings demonstrate that MEK-ERK signaling could restrict cell growth or promote aberrant cell proliferation, perhaps depending on the mutational profile of cells. It is also possible that inhibition of cell growth by Ras-ERK signaling is dependent on the triggers activating this pathway and/or the subcellular location of the signaling complex formed (*89*).

The Ras-ERK pathway is dysregulated across multiple cancers (*57*, *90*). Small-molecule inhibitors targeting components of the Ras-ERK signaling have been clinically tested for the treatment of KRAS and BRAF mutated colorectal cancer; however, these inhibitors tend to acquire resistance and lead to bypass activation of Ras-ERK signaling, resulting in ineffective clinical outcomes (*91*). For instance, Raf inhibitors such as vemurafenib are effective in the treatment of BRAF mutant melanoma; however, BRAF mutant colorectal cancer is resistant to vemurafenib due to feedback up-regulation of ERK signaling (*92*, *93*). We found that dabrafenib-mediated inhibition of Raf might reduce the binding strength between Ras with Raf, and between Ku70 and Ras, which in turn might affect the signaling of the Ku70-Ras-Raf complex. Inhibitors of MEK have also been tested as potential therapeutics for the treatment of colorectal cancer; however, their efficacy is limited because of the paradoxical activation of ERK (*57*, *91*). Moreover, previous studies have shown that MEK inhibition using trametinib promotes the expression of genes associated with cancer development (*51*, *52*). Furthermore, small-molecule inhibitors of ERK either show no efficacy or produce a partial clinical response in patients with KRAS- and BRAF-mutant colorectal cancer (*91*). Although targeting Ras-ERK has been successful in the treatment of several human cancers including melanoma, more innovative and effective therapeutic approaches are warranted to target the Ras-ERK signaling pathway in colorectal cancer. We identified that the α/β domain and the C-terminal domain of Ku70 facilitate the formation of the Ku70-Ras-Raf complex. Notably, the α/β domain and the C-terminal domain Ku70 are also required for the recognition of double-stranded DNA breaks and binding to Ku80, respectively (*94*, *95*). Identifying the precise amino acid residues within the α/β domain and the C-terminal domains of Ku70 might aid in designing small-molecule activators or inhibitors of Ku70 with more precision while minimizing interference with other biological functions of Ku70 including DNA repair.

Previous studies have shown that the switch II region within the GTPase domain of Ras is required for interaction with Raf (*50*), whereas the CR1 domain of Raf facilitates its interaction with Ras (*56*, *96*). We observed that the switch II region of Ras or CR1 domain of Raf does not interact with Ku70. Instead, we found that the GTPase domain of Ras (in the absence of switch I and switch II regions) and the CR2 domain of Raf-1 interact with Ku70. While these findings could aid in the design of therapies benefiting patients with intestinal cancer who have a reduced expression of Ku70, our findings could also provide insights into the treatment of cancers with hyperactivated Ras-ERK signaling. We speculate that small-molecule inhibitors and/or antagonists targeting Ras and Raf-1 at domains and/or regions that are not required to form the Ku70-Ras-Raf signalosome might be beneficial in controlling the development and/or progression of cancers with hyperactivated Ras-ERK signaling. Future studies using transgenic mice or small-molecule targeting the Ku70-Ras-ERK pathway could highlight the divergent roles of this signaling pathway in colorectal cancer.

Double-strand DNA breaks severely compromise genomic stability, leading to cancer development if not appropriately repaired (*97*). We found that the lack of Ku70 does not affect the level of DNA damage marked by phosphorylated H2AX or expression of 53BP1 in the mouse intestine. Similarly, a previous study has shown that DNA damage and cell cycle checkpoints, including G_1_-S and G_2_-M DNA damage checkpoints, are not affected in Ku70-deficient mouse embryonic fibroblasts (*25*). Furthermore, Ku70 has been shown to induce the expression of the antiviral cytokine IFN-λ (*20*–*22*) and potentially limits the production of proinflammatory cytokines IL-6 and tumor necrosis factor (TNF) (*27*). However, we did not observe a difference in the expression of IFNs and other inflammatory mediators among WT, *Ku70*^+/−^, and *Ku70*^−/−^ mice during intestinal tumorigenesis. These findings suggest that Ku70 might mediate its biological functions in a cell type–specific and/or disease-specific manner. In this study, we did not determine the cell type–specific functions of Ku70-mediated Ras-ERK signaling in mice. We did not identify whether the expression of Ku70 in nontumor cells and tumor cells is protective or detrimental. In some cases, elevated expression of Ku70 can render tumor cells, such as cervical or rectal tumor cells, radioresistant, which might then hinder the efficacy of radiotherapy (*63*, *98*). Therefore, determining the cell type–specific functions of Ku70-mediated Ras-ERK signaling could help in improving the precision of targeted therapy against cancer. We speculate that the activation of the Ku70-mediated Ras-ERK signaling might be initiated by the cytoplasmic DNA arising from the gut microbiome introduced into the host cells following a rupture of the intestinal barrier. However, it is also possible that damaged nucleus and/or mitochondria may be a source of cytoplasmic DNA that triggers Ku70-mediated Ras-ERK signaling. Furthermore, investigations into the precise source and feature of DNA involved in activating the Ku70-mediated Ras-ERK signaling pathway could provide further insights into the molecular mechanisms of Ku70 activation and could guide the development of nucleic acid–based therapeutics (*99*).

## MATERIALS AND METHODS

### Mice

*Ku70*^−/−^ mice on the 129 background have been described previously (*25*, *28*). Littermate WT, *Ku70*^+/−^, and *Ku70*^−/−^ mice on 129 background were bred and maintained at The Australian National University (ANU). C57BL/6NcrlAnu mice were obtained from the Australian Phenomics Facility (APF) at the ANU. Littermate *Ku70*^+/−^ and *Ku70*^−/−^ mice on 129 background were also backcrossed to the C57BL/6NcrlAnu background for three generations. *Apc*^Min/+^ mice on the C57BL/6NcrlAnu background were obtained from the APF at the ANU. Mice were housed and maintained in specific pathogen–free conditions by Animal Staff Technicians of the APF at the ANU. All experiments were conducted under the oversight of ANU Animal Ethics Committee according to protocol number A2020/18.

### Experimental colitis and colitis-associated colorectal tumorigenesis

Male and female littermate WT, *Ku70*^+/−^, and *Ku70*^−/−^ mice of 8 to 10 weeks were injected intraperitoneally with 10 mg of AOM (A5486, Sigma-Aldrich) per kilogram body weight. Five days later, 1.5% DSS (160110, MP Biomedicals) was given in the drinking water for 6 days, followed by regular drinking water for 2 weeks. This cycle was repeated twice with 1.5% DSS. Mice were ethically culled on day 80, colon tissues were harvested, and the colon length was measured. Tumor numbers were enumerated, and the colon tissue was stored at −80°C or fixed in 10% neutral-buffered formalin (NBF) (HT501320, Sigma-Aldrich) for further analysis.

For colitis induction, mice were injected with AOM and, after 5 days, fed with 1.5% DSS for 6 days. Mice were then given regular water for 3 days and were ethically culled on day 14. On day 14, colon tissues were harvested, colon length was measured, and the tissue was stored at −80°C or fixed in 10% NBF for further analysis.

### *Apc*^Min/+^ model of spontaneous intestinal cancer

*Apc*^Min/+^ mice from the C57BL/6N were crossed with *Ku70*^+/−^ mice from the 129 background to generate littermate *Apc*^Min/+^ *Ku70*^+/+^, *Apc*^Min/+^ *Ku70*^+/−^, and *Apc*^Min/+^ *Ku70*^−/−^ mice. The colon and small intestine were collected from mice at 20 weeks of age. The intestinal length was measured, and tumor numbers were enumerated. Investigators were blinded to the genotypes of the mice during tumor counting. Tissues were stored at −80°C or fixed in 10% NBF for further analysis.

### Histology and microscopy analysis

The colon and distal small intestine of mice were rolled into a “Swiss roll” and fixed in 10% NBF. Tissues were processed and embedded in paraffin and sectioned at 5 μm in thickness onto positively charged microscopy slides. The tissue sections were stained with hematoxylin and eosin. Histology scores were assigned on the basis of inflammation, hyperplasia, and/or adenoma as described previously (*100*). Hematoxylin and eosin–stained colon tissue sections were obtained and analyzed by anatomical region as proximal (human equivalent; ascending colon), middle (transverse) and distal (descending) colon, and rectum. The percentage of tissue areas affected by inflammation, hyperplasia, and/or adenoma was calculated separately by anatomical region. Similarly, hematoxylin and eosin–stained distal small intestinal tissue sections were divided arbitrarily into four segments: segment 1, closest to the jejunum (40% of total tissue area), segment 2 (30% of total tissue area), segment 3 (20% of total tissue area), and segment 4 (10% of total tissue area) consecutively thereafter. The percentage of tissue areas affected by inflammation, hyperplasia, and/or adenoma was calculated separately in each segment. The extent of damage was then calculated as a combination of inflammation, hyperplasia, and/or adenoma in the total tissue area and presented as a histology score.

### Immunohistochemistry and microscopy analysis

For immunohistochemistry, slides containing tissue sections were deparaffinized in xylene (534056, Sigma-Aldrich) and rehydrated in decreasing concentrations of ethanol (107017, Merck) before being washed in phosphate-buffered saline (PBS; pH 7.1 to 7.5) (D8537, Sigma-Aldrich) and tris-buffered saline containing 1% Tween 20 (TBST) [200 mM tris-HCl (pH 7.4) and 1.37 M NaCl, 0.1% (v/v) Tween 20]. The slides were then subjected to antigen retrieval by heating in sodium citrate buffer [10 mM sodium citrate (pH 6.0) and 0.05% Tween 20] at 95°C in a water bath for 10 min followed by cooling at room temperature for 30 min. The slides were then washed once each for 5 min in TBST and distilled water, followed by an hour of incubation in blocking buffer [10% normal goat serum (005000121 Jackson ImmunoResearch) in PBS supplemented with 0.3% Triton X-100]. The slides were then incubated overnight at 4°C with primary antibodies diluted in the antibody dilution buffer [1% bovine serum albumin (BSA; A7030, Sigma-Aldrich) in PBS supplemented with 0.1% Triton X-100]. Primary antibodies used for immunohistochemistry were AF488-tagged CD324 (also E-cadherin; 1:200; Invitrogen, Thermo Fisher Scientific), AF647-tagged CD45R (1:100; BD Biosciences), claudin 2 (1:200, Abcam, ab53032), DNA (1:100; EMD Millipore, MAB1293), Ki67 (1:200; Abcam, ab15580), Ku70 (1:100; Santa Cruz Biotechnology, sc-17789), PCNA (1:200; Novus Biologics, NB500-106), phospho-ERK1/2 Thr^202^/Tyr^204^ (1:200; Cell Signaling Technology, 9101), phospho–histone H2A.X (1:100; Santa Cruz Biotechnology, sc517348), phospho-MEK Ser_221_ (1:1000; Cell Signaling Technology, 2338), Raf-1 (1:100; Santa Cruz Biotechnology, sc-7267), occludin (1:200; Thermo Fisher Scientific, 710192), Ras (1:200; Cell Signaling Technology, 67648), β-actin (1:300; Abcam, ab8226), and β-actin (1:200; Cell Signaling Technology, 4970). For the DNA staining, tissue sections were incubated in the blocking buffer containing ribonuclease A (1:100, QIAGEN, 1007885). After overnight incubation in primary antibodies, slides were washed three times with TBST for 5 min each. The slides were then incubated at room temperature for 1 hour in antibody dilution buffer containing 4′,6-diamidino-2-phenylindole (DAPI) (1 μg/ml; D9542, Sigma-Aldrich) and secondary antibodies. Secondary antibodies used for immunohistochemistry were Alexa Fluor 488 AffiniPure goat anti-rabbit (1:300; Jackson ImmunoResearch, 111-545-144), Alexa Fluor 488 AffiniPure goat anti-mouse (1:300; Jackson ImmunoResearch, 115-545-003), Rhodamine Red-X-AffiniPure goat anti-rabbit (1:300; Jackson ImmunoResearch, 111-295-144), and Rhodamine Red-X-AffiniPure goat anti-mouse (1:300; Jackson ImmunoResearch, 115-295-146). After 1 hour, the slides were washed three times for 5 min with TBST and once for 5 min with distilled water. Tissue sections were then protected with a 22 mm–by–22 mm coverslip (0101050, Superior Marienfeld) using ProLong Gold Antifade mounting media (P36930, Thermo Fisher Scientific) and left to dry overnight in the dark at room temperature. Samples were visualized using the Zeiss Axio Observer microscope. Image analysis and quantification were performed using ImageJ (National Institutes of Health, USA). The percentage of tissue areas containing the fluorescence signal of the target protein was calculated and divided by the whole tissue area containing the fluorescence signal of β-actin (control protein) and the nuclear marker DAPI. Investigators were blinded from the genotype of the mice and treatment groups during image acquisition and quantification. For the 3,3′-diaminobenzidine (DAB)–based immunohistochemistry, tissue sections were processed according to Leica BOND Protocol using the automated Bond RX platform (Leica Biosystems). Primary antibodies used for DAB-based immunohistochemistry were CD4 (1:200; Cell Signaling Technology, 25229), CD8 (1:400; Cell Signaling Technology, 98941), F4/80 (1:200; Cell Signaling Technology, 70076), FoxP3 (1:500; Cell Signaling Technology, 12653), and Ly-6G (1:600; Cell Signaling Technology, 87048). Stained slides were then scanned using ZEISS Axio Scan.Z1 Slide Scanner, and the images were analyzed using QuPath v0.4.3.

### Immunoblotting analysis of the mouse colon tissue

The colon tissue from mice was lysed in 1 ml of radioimmunoprecipitation assay (RIPA) [20 mM tris-HCl (pH 7.4), 150 mM NaCl, 1% Triton X-100, 0.5% sodium deoxycholate, 0.1% SDS, and protease inhibitor cocktail tablet (11836145001, Roche)] buffer using the Omni tissue homogenizer (TH-2, Omni international). Lysates were cleared of insoluble material by centrifugation at 12,000*g* for 10 min at 4°C. The protein concentration of each sample was normalized using Pierce bicinchoninic acid (BCA) protein assay kit (23227; Thermo Fisher Scientific) according to the manufacturer’s instructions. Lysates were then mixed with 4× sample loading dye [200 mM tris-HCl (pH 6.4), 400 mM dithiothreitol, 8.0% (w/v) SDS, 0.4% bromophenol, and 40% glycerol] and boiled at 100°C for 5 min. Samples were separated on 8 to 15% polyacrylamide gels using the Trans-Blot Turbo system (Bio-Rad). Proteins were transferred onto the polyvinyldifluoride membrane (IPVH00010, Millipore), which were preactivated using methanol (A452, Fisher Chemical) and equilibrated using transfer buffer [250 mM tris-HCl (pH 8.3) and 1.92 M glycine]. Membranes were then blocked in 5% skim milk in TBST for 1 hour before incubation overnight at 4°C in primary antibodies. Primary antibodies used for immunoblotting were c-Raf (also called Raf-1, 1:1000; Cell Signaling Technology, 12552), calreticulin (1:1000; Abcam, ab92516), caspase-11 (1:1000; Novus Biologicals, NB120-10454), COXIV (1:500; Thermo Fisher Scientific, PA5-19471), DNA-PKcs (1:500; Santa Cruz Biotechnology, sc-390698), phospho-EGFR Tyr^992^ (1:1000; Cell Signaling Technology, 2235), phospho-EGFR Tyr^1068^ (1:1000; Cell Signaling Technology, 3777), total EGFR (1:1000; Cell Signaling Technology, 4267), GFP (1:2000; Cell Signaling Technology, 2956), GM130 (1:1000; BD Biosciences, 610822), hemagglutinin (HA) tag (1:1000; Cell Signaling Technology, 3724), phospho-IκBα Ser^32^ (1:1000; Cell Signaling Technology, 2859), total IκBα (1:1000; Cell Signaling Technology, 9242), Ku70 (1:1000; Cell Signaling Technology, 4588), Ku70 (1:500; Santa Cruz Biotechnology, sc-17789), Ku86 (1:500; Santa Cruz Biotechnology, sc-515736), pan-cadherin (1:1000; Abcam, ab51034), phospho-Cdc25A Ser^124^ (1:1000; Abcam, ab156574), phospho-CDK1 (also called phospho-Cdc2) Tyr^15^ (1:1000; Cell Signaling Technology, 4539), phospho-CDK2 Thr^160^ (1:1000; Cell Signaling Technology, 2561), phospho-Chk2 Thr^68^ (1:1000; Cell Signaling Technology, 2197), phospho-ERK1/2 Thr^202^/Tyr^204^ (1:3000; Cell Signaling Technology, 9101), phospho-GSK3β Ser^9^ (1:1000; Cell Signaling Technology, 5558), phospho–histone H2A.X (1:500; Santa Cruz Biotechnology, sc-517348), phospho-JNK Thr^183^/Tyr^185^ (1:1000; Cell Signaling Technology, 4668), phospho-MEK1/2 Ser^221^ (1:1000; Cell Signaling Technology, 2338), phospho-p38 Thr^180^/Tyr^182^ (1:1000; Cell Signaling Technology, 4511), phospho-PKCβ Ser^660^ (1:1000; Cell Signaling Technology, 9371), phospho-RSK3 Thr^356^/Ser^360^ (1:1000; Cell Signaling Technology, 9348), Rab5 (1:3000; Abcam, ab218624), Rab7 (1:2000; Abcam, ab126712), Ras (1:2000; Abcam, ab108602), phospho-Stat3 Ser^727^ (1:1000; Cell Signaling Technology, 9134), SQSTM1/p62 (1:1000; Abcam, ab109012), TGN38 (1:500; Santa Cruz Biotechnology, sc-166594), total Cdc25A (1:1000; Abcam, ab92892), total CDK1 (also called total Cdc2) (1:1000; Cell Signaling Technology, 28439), total Chk2 (1:1000; Abcam, ab47433), total ERK1/2 (1:3000; Cell Signaling Technology, 9102), total MEK1/2 (1:1000; Cell Signaling Technology, 4694), total Stat3 (1:3000; Cell Signaling Technology, 9139), V5 tag (1:1000; Cell Signaling Technology, 13202), β-actin (1:3000; Abcam, ab8226), and β-actin (1:5000; Cell Signaling Technology, 4970).

Following overnight incubation in the primary antibody, membranes were washed four times for 5 min with TBST. Membranes were then incubated in horseradish peroxidase–conjugated secondary antibodies at 1:5000 dilution in 5% skim milk in TBST for 1 hour at room temperature. Secondary antibodies used for immunoblotting were Peroxidase AffiniPure (1:5000; Jackson ImmunoResearch, 111-035-045) and Peroxidase AffiniPure (1:5000, Jackson ImmunoResearch, 115-035-146). The membranes were then washed four times for 5 min with TBST. The membranes were then incubated with Clarity Western ECL Substrate (1705061, Bio-Rad) for 2 min or SuperSignal West Atto Ultimate Sensitivity Substrate (A38554, Thermo Fisher Scientific) for 1 min followed by imaging using the ChemiDoc Imaging System (12003153, Bio-Rad). Image processing and densitometric quantification of protein bands were performed using the Image Lab software (Bio-Rad).

### Enzyme-linked immunosorbent assay

Colon tissue lysates were prepared as described above. Cytokines from the colon tissue lysates were analyzed using plate-based ELISA kits according to the manufacturer’s instructions. IL-18 was quantified using a simplex ELISA kit (BMS618-3TEN, Thermo Fisher Scientific; or EK-0048, ELISAkit.com), and all other cytokines were quantified by a multiplex ELISA kit (MCYTMAG-70K-PX32, EMD Millipore). Phospho-ERK/12 and total ERK/12 were quantified in the colon and small intestinal tissue lysates using the ERK1/ERK2 (Total/Phospho) Multispecies InstantOne ELISA Kit (858601311, Invitrogen, Thermo Fisher Scientific).

### Quantitative RT-PCR

The colon tissue from untreated or AOM-DSS–treated mice was homogenized in 1 ml of TRIzol (15596018, Thermo Fisher Scientific). Chloroform (C2432, Sigma-Aldrich) was added (200 μl) to each sample, and the mixture was vortexed vigorously for 15 s, followed by incubation for 15 min at room temperature. Samples were then centrifuged at 12,000*g* for 20 min at 4°C, and the aqueous phase was transferred to a fresh tube containing 500 μl of 2-propanol (818766, Merck), followed by incubation for an hour at 80°C. Samples were thawed at room temperature followed by centrifugation at 12,000*g* for 20 min at 4°C. The supernatant was discarded, and the pellet was dissolved in a solution containing 500 μl of water (10977015, UltraPure DNase/RNase-Free Distilled Water, Invitrogen, Thermo Fisher Scientific), 50 μl of 3 M sodium acetate (pH 5.5) (AM9740, Invitrogen), and 500 μl of 2-propanol. Samples were incubated for 20 min at room temperature, followed by centrifugation at 12,000*g* for 10 min at 4°C. The supernatant was discarded, and the pellet was washed twice with 500 μl of 75% ethanol (E7023, Sigma-Aldrich) by centrifugation at 12,000*g* for 10 min at 4°C. The supernatant was discarded, and the pellet was air-dried before being dissolved in 50 μl of ultrapure water.

RNA was further purified via the lithium chloride (LiCl) precipitation method as described previously (*101*). Briefly, 5 μl of 8 M LiCl (L7026, Sigma-Aldrich) was added to 50 μl of RNA, and the mixture was incubated for 2 hours on ice. After incubation, samples were centrifuged at 14,000*g* for 30 min at 4°C. The supernatant was discarded, and the pellet was dissolved in 200 μl of water. Twenty microliters of 8 M LiCl was added to each sample, and the mixture was incubated for 2 hours on ice. After incubation, samples were centrifuged at 14,000*g* for 30 min at 4°C. The supernatant was discarded, and pellets were dissolved in 200 μl of water. Twenty microliters of 3 M sodium acetate and 400 μl of −20°C prechilled 100% ethanol were added to each sample followed by incubation for 30 min at −20°C. After incubation, samples were centrifuged at 14,000*g* for 30 min at 4°C. The supernatant was discarded, and the pellet was washed with 100 μl of −20°C prechilled 70% ethanol by centrifugation at 14,000*g* for 10 min at 4°C. The supernatant was discarded, and the pellet was air-dried before being dissolved in 50 μl of water. The concentration of RNA was measured using the NanoDrop One spectrophotometer (Thermo Fisher Scientific).

A total of 2 μg of RNA was converted to cDNA using the High-Capacity cDNA reverse transcription kit (4368813, Thermo Fisher Scientific). The expression of genes was assessed using the PowerUp SYBR Green Master Mix (A25742, Thermo Fisher Scientific) according to the manufacturer’s instructions. The sequences of the target genes encoding glyceraldehyde-3-phosphate dehydrogenase (GAPDH) (*5*), Ku70 (Harvard PrimerBank), IFN-β (*100*), IFN-γ (*100*), IFN-λ (*102*), ATM (*103*), CHK1 (*104*), Rad51 (*105*), β-catenin (*100*), Axin 2 (*100*), and Lgr5 (*100*) are provided in table S3.

### 16*S* rRNA sequencing of the gut microbiota

Genomic DNA was extracted from mouse feces using the QIAamp PowerFecal Pro DNA Kit (51804, QIAGEN). 16*S* ribosomal RNA (rRNA) gene sequencing was performed as described previously (*106*). The V4 region of the 16*S* rRNA gene was amplified using the Kapa HiFi HotStart ReadyMix and the Earth microbiome primers (515F and 806R) as described previously (*107*, *108*). The following parameters were used for amplification: 95°C for 3 min; 25 cycles of 95°C for 30 s, 55°C for 30 s, and 72°C for 30 s, followed by a final step of 72°C for 5 min (*107*). Indices and Illumina sequencing adapters were attached using the Nextera XT index kit, and sequencing was performed with Illumina MiSeq 2 × 250–base pair chemistry at the Ramaciotti Centre for Genomics. Three negative control samples (extraction kit reagents) were included as part of the sequencing run. Raw reads were analyzed using mothur v1.42.3 with Silva SEED 16*S* rRNA reference (v123) alignment, clustering with opticlust method, and classification using RDP v16 (3%). The resulting data matrix was subsampled (read depth, 13,125 clean reads per sample) and used for statistical analysis. Microbiota data were deposited to Zenodo and are available from DOI: 10.5281/zenodo.8174039.

### Phospho-mass spectrometry

Proteins were extracted from the colon tissue of mice as described above. The protein concentration for each sample was normalized using the BCA Protein Assay Kit according to the manufacturer’s instructions. A total of 500 μg of protein was digested with trypsin and purified with Oasis HLB cartridges (Waters Corp.). Serine-, threonine-, and tyrosine-phosphorylated peptides were enriched using the Titansphere Phos-TiO Spin Tip kit (GL Sciences) according to the manufacturer’s instructions. Each sample was then resuspended in 10 μl of 1% formic acid, 0.05% heptafluorobutyric acid, and 2% acetonitrile. Samples were then analyzed by liquid chromatography tandem mass spectrometry (LC-MS/MS) by loading 5 μl of each sample onto a Fusion Lumos (Thermo Fisher Scientific) connected to an UltiMate nanoRSLC UPLC and autosampler system (Dionex) as previously described (*109*). Raw mass spectrometric files were analyzed using MaxQuant v2.0.3.0 (*110*) using the *Mus musculus* UniProt proteome (UP000000589_10090) as a reference. A heatmap of differentially phosphorylated proteins was created using the Heatmapper web server, assisted by the average linkage method for clustering and the Pearson method for distance measurement (*111*). Genes encoding the differentially phosphorylated proteins were subjected to kinase and phosphatase enrichment analysis using the Enrichr web server (*37*). Phosphoproteomic data have been deposited to Zenodo and are available from DOI: 10.5281/zenodo.8174072.

### Plasmid constructs and transformation

The DNA sequence encoding full-length human Ku70 or its domains (CCDS ID: 14021.1) was fused to the DNA sequence encoding a FLAG-tag at the N terminus. The DNA sequence encoding full-length human H-Ras or its domains (CCDS ID: 7698.1) was fused to the DNA sequence encoding a HA tag at the N terminus. The DNA sequence encoding full-length human Raf-1 (also called c-Raf) or its domains (CCDS ID: 2612.1) was fused to the DNA sequence encoding a V5 tag at the N terminus. Constructs were synthesized by GenScript and cloned into a pcDNA3.1(+)-N-eGFP vector backbone between the restriction sites Kpn l and Bam HI for Ku70 plasmids and between restriction sites Kpn l and Xho l for H-Ras and Raf-1 plasmids.

For transformation, plasmid constructs were dissolved in 50 μl of ultrapure distilled water. Each plasmid (200 ng) was transformed into 50 μl of NEB 5-alpha competent *Escherichia coli* (C2987H, New England Biolabs) as per the manufacturer’s instructions. Briefly, the bacteria-plasmid mixture was placed on ice for 45 min, heat-shocked in a 42°C water bath for 30 s, and returned to the ice for 5 min. The bacteria were then supplemented with 900 μl of Super optimal broth with catabolite repression (SOC) media (15544-034, Thermo Fisher Scientific) and incubated at 37°C in aerobic conditions for an hour with shaking (250 rpm). The transformed bacteria were diluted 1:1000 in SOC media, and 100 μl of the diluted bacteria was spread onto LB agar (LBAmp) [LB broth + 1.5% (w/v) Bacto Agar Solidifying Agent (9000-760, BD Biosciences)] containing ampicillin (100 μg/ml; A9393, Sigma-Aldrich). Single colonies were used to inoculate 5 ml of LBAmp broth and grown aerobically overnight at 37°C, with constant agitation (200 rpm). The overnight culture was used to inoculate 200 ml of LB that was grown as described above for 16 hours, and the biomass was harvested by centrifugation. Plasmids were extracted from bacterial pellets using the Plasmid Plus Maxi Kit (12963, QIAGEN) as per the manufacturer’s instructions. Plasmid DNA quantification was performed using the NanoDrop One spectrophotometer (Thermo Fisher Scientific).

### Isolation of genomic DNA

DNA from mouse colon tissue and HEK293T cells was isolated using the QIAamp DNA mini kit (51304, QIAGEN) as per the manufacturer’s instructions. Mouse fecal DNA and *E. coli* (bacterial) DNA were isolated using the QIAamp PowerFecal Pro DNA Kit (51804, QIAGEN) as per the manufacturer’s instructions. The concentration of the DNA was measured using the NanoDrop One spectrophotometer (Thermo Fisher Scientific).

### Cell culture, transfection, and stimulation

HEK293T cells were cultured in Dulbecco’s modified Eagle’s medium (D6546, Sigma-Aldrich) supplemented with 10% fetal bovine serum (FBS) and 1% penicillin-streptomycin-glutamine (10378016, Gibco, Thermo Fisher Scientific). Cells were seeded at a concentration of 1 × 10^6^ cells per well into a six-well plate (CLS3516, Sigma-Aldrich) and incubated overnight at 37°C. Cells were transfected using Xfect Polymer (631318, Takara Bio USA Inc.) as per the manufacturer’s instructions. Briefly, 2 μg of plasmid DNA was mixed with the Xfect polymer (0.3 μl of Xfect polymer per 1 μg of DNA). The mixture was then made up to 100 μl with Xfect reaction buffer, followed by vortexing for 10 s. After 10 min of incubation at room temperature, the mixture was slowly added to the cells. Four hours after the transfection, the cell culture medium was replaced with fresh medium, and plates were incubated for 48 hours at 37°C. After 48 hours, cells were processed for coimmunoprecipitation or immunoblotting as described above.

HEK293T cells were seeded at a concentration of 0.5 × 10^6^ per well into a 12-well cell culture plate (CLS3513, Sigma-Aldrich) and incubated overnight at 37°C. Cells were transfected with human DNA from HEK293T cells (1 μg), mouse colonic DNA (1 μg), mouse fecal DNA (1 μg), bacterial DNA from *E. coli* DNA (1 μg), poly(dA:dT) [tlrl-patn, InvivoGen] (1 μg), CpG DNA [ODN2006, InvivoGen] (1 μg), mouse colonic RNA (1 μg), and poly(I:C) [tlrl-pic, InvivoGen] (1 μg) or stimulated with LPS (1 μg) or IGF-1 (50 ng). All the nucleic acid transfection was done using Xfect Polymer as described above. After 6 hours, cells were processed for immunoblotting or immunofluorescence staining as described below. For the inhibitor study, HEK293T cells were serum-starved for 48 hours and left untreated or pretreated with STL127705 (25 μM) for 1 hour. Cells were then left nontransfected or transfected with human DNA from HEK293T cells (1 μg), mouse colonic DNA (1 μg), or mouse colonic RNA (1 μg) followed by incubation for 6 hours at 37°C. Cells were then processed for immunoblotting as described below. In a separate set of experiments, HEK293T cells were either left nontransfected or transfected with an empty vector or vector containing Ku70 plasmid. Forty-eight hours after transfection, the cells were treated with dabrafenib (2 μM) for 2 hours followed by coimmunoprecipitation and immunoblotting as described above. For irradiation studies, HEK293T cells were seeded at a concentration of 0.5 × 10^6^ per well into a 12-well cell culture plate (CLS3513, Sigma-Aldrich) and incubated overnight at 37°C. Cells were left untreated or radiated for 6 min (1.2 Gy) or 20 min (4 Gy) using X-Ray Irradiator RS2000 (Rad Source Technologies). Cells were then processed for immunofluorescence staining as described below.

Pinnae of WT, *Ku70*^+/−^, and *Ku70*^−/−^ mice were incubated in ethyl alcohol for 5 min. After drying, ears were minced and digested with collagenase D (5 mg/ml; 11088866001, Roche) and pronase (3 mg/ml; 53702-25KU, EMD Millipore Corp.) for 90 min. Cell suspension was filtered through 70μm strainers and cultured in complete media [RPMI 1640 (21870-076, Gibco) containing 20% FBS (F8192, Sigma-Aldrich), 10 mM Hepes (15630-080, Gibco), 1% penicillin-streptomycin-glutamine (10378016, Gibco), 1% sodium pyruvate (11360-070, Gibco), and 50 μM 2mercaptoethanol (21985-023, Gibco)] overnight. After overnight culture, cells were washed once with PBS and incubated in complete media for 3 to 4 days. Cells were seeded at a density of 1.5 × 10^5^ cells per well into a 12-well plate (CLS3513, Sigma-Aldrich). Cells were transfected with mouse colonic DNA (1 μg) using Xfect Polymer as described above. After 6 hours, cells were processed for qRT-PCR as described above.

### Immunoblotting and immunofluorescence staining of HEK293T cells

Transfected or nontransfected HEK293T cells were washed once with PBS, and 0.25% trypsin-EDTA was added to the wells. After incubation at 37°C for 5 min, cells were collected by centrifugation at 500*g* for 3 min at 4°C. The supernatant was discarded, and the pellet was washed once with PBS. Cells were then lysed using RIPA buffer. Lysates were cleared of insoluble material by centrifugation at 12,000*g* for 10 min at 4°C. Lysates were then mixed with 4× sample loading dye and boiled at 100°C for 10 min. Immunoblotting was performed as described above.

For immunofluorescence staining, HEK293T cells were seeded onto coverslips of 18 mm thickness (0111580, Superior Marienfeld) inside a 12-well cell culture plate (CLS3513, Sigma-Aldrich) at a density of 0.5 × 10^6^ cells per coverslip. After the indicated treatment, cells were washed once with PBS and fixed with 4% paraformaldehyde for 15 min at room temperature. The samples were blocked in 10% normal goat serum supplemented with 0.1% saponin (47036, Sigma-Aldrich) for an hour at room temperature. Cells were incubated with primary antibodies overnight at 4°C. Primary antibodies used for immunofluorescence assays were phospho–histone H2A.X (1:200; Santa Cruz Biotechnology, sc-517348), Ku70 (1:200; Santa Cruz Biotechnology, sc-17789), and Rab5 (1:500; Abcam, ab218624). The next day, cells were washed three times using PBS for 5 min each. Cells were incubated with secondary antibodies at room temperature. Secondary antibodies used for immunofluorescence assay were Alexa Fluor 488 AffiniPure goat anti-mouse (1:300; Jackson ImmunoResearch, 115-545-003) and Rhodamine Red-X-AffiniPure goat anti-rabbit (1:300; Jackson ImmunoResearch, 111-295-144). After an hour of incubation with secondary antibodies, cells were washed three times using PBS for 5 min each. Coverslips were then mounted on microscopy slides using ProLong Gold Antifade mounting media and left to dry overnight in the dark at room temperature. Samples were visualized using the Zeiss LSM800 microscope. The Pearson’s correlation coefficient and quantification of P-H2A.X were performed using ImageJ (National Institutes of Health, USA). Investigators were blinded of the treatment groups during image acquisition and quantification.

### Coimmunoprecipitation

Colon tissues or HEK293T cells were lysed in lysis buffer [50 mM tris-HCl (pH 7.5), 150 mM NaCl, 1% NP-40, 1% glycerol, and protease inhibitor cocktail tablet (11836145001, Roche)]. Lysates were cleared of insoluble material by centrifugation at 12,000*g* for 10 min at 4°C. For immunoprecipitation, the tissue or cell lysates were incubated in primary antibodies at room temperature or overnight at 4°C. Species-specific immunoglobulin G (IgG) antibodies were used as a control. Antibodies used for coimmunoprecipitation were GFP (1:200; Cell Signaling Technology, 2955), HA tag (1:200; Cell Signaling Technology, 2367), IgG (1 μl/ml, Cell Signaling Technology, 5415), IgG (1 μl/ml, Cell Signaling Technology, 3900), Ku70 (1:50; Santa Cruz Biotechnology, sc-17789), Rab5 (1:200; Abcam, ab218624), Rab7 (1:200; Abcam, ab126712), Ras (1:300; Abcam, ab108602), and V5 tag (1:200; Cell Signaling Technology, 80076). Following incubation with primary antibody, 20 μl of Protein A/G PLUS-Agarose beads (sc-2003, Santa Cruz Biotechnology) was added to the lysates and the mixture was further incubated for 2 hours at 4°C. The immunoprecipitated products were washed three times with lysis buffer and released from the agarose beads using 4× sample loading dye and boiled at 100°C for 5 min. Immunoblotting was performed as described above.

### Proliferation assay

HEK293T cells were seeded at a density of 5000 cells per well in 96-well tissue culture plates (3595, Corning) and incubated overnight at 37°C. Cells were then transfected with an empty vector or vector containing Ku70 plasmid. Cell proliferation was measured using the WST-1 reagent (05015944001, Roche). The cell culture medium was changed every day, and WST-1 reagent was added to the cells for 2 hours before reading at 450 nm. Cell proliferation was expressed as the absorbance or optical density. For assessment of cell proliferation in real time, HEK293T cells were seeded at a density of 200,000 cells per well in six-well tissue culture plates (CLS3516, Sigma-Aldrich) and incubated overnight at 37°C. Cells were then transfected with an empty vector or vector containing Ku70 plasmid. Cell proliferation was monitored over time using IncuCyteS3, and data were collected using IncuCyte v2021A.

### Colon organoid culture, stimulation, and analysis

Mouse colonic organoids were cultured using IntestiCult organoid growth medium (06000, STEMCELL Technologies) according to the manufacturer’s instructions. Briefly, the whole colon was removed and flushed twice with cold PBS. The colon was scrapped using a microscopy slide to remove mucus and cut into 2- to 5-mm segments. Colon segments were washed 15 times with cold PBS followed by incubation in Gentle Cell Dissociation Reagent (07174, STEMCELL Technologies) with gentle shaking for 15 min at room temperature. Colon segments were then resuspended in PBS supplemented with 0.1% BSA, and dissociated crypts were filtered through a 70-μm cell strainer (CLS431751, Sigma-Aldrich). The dissociated crypts were then resuspended in PBS containing 0.1% BSA and 10% FBS (F8192, Sigma-Aldrich) and centrifuged at 300*g* for 5 min at 4°C. The crypts were resuspended in IntestiCult organoid growth medium and Matrigel (356230, Corning) in a 1:1 ratio and were plated in 24-well culture plates (CLS3524, Sigma-Aldrich). The IntestiCult organoid growth medium was added to the plate to immerse the matrix composed of the IntestiCult organoid growth medium and Matrigel. The culture medium was changed every 2 days.

For growth analysis, images of organoids were captured using the Leica DMi1 microscope, and the perimeter of organoids was measured using ImageJ. Investigators performing analysis on organoids were blinded from the genotype and treatment. On day 6, organoids were left untreated or treated with recombinant murine IGF-1 (50 ng/ml) (250-19, PeproTech). After removing the culture media, organoids were washed with PBS, and 0.25% trypsin-EDTA (25200-056, Gibco, Thermo Fisher Scientific) was added to the wells. After incubation for 5 min at 37°C, organoids were dissociated into smaller fragments by pipetting. Cells were pelleted by centrifugation at 500*g* for 3 min at 4°C. Cell pellets were washed once with PBS, and cells were lysed using RIPA buffer. Lysates were cleared of insoluble material by centrifugation at 12,000*g* for 10 min at 4°C. Lysates were then mixed with 4× sample loading dye and boiled at 100°C for 10 min. Immunoblotting was performed as described above.

For immunofluorescence staining, organoids were cultured on a 13-mm coverslip (0111530, Superior Marienfeld) in the wells of 24-well plates. On days 2, 4, and 6, culture medium was removed, and organoids were washed twice with PBS. Organoids were fixed in 4% paraformaldehyde (28908, Thermo Fisher Scientific) for 30 min at room temperature, followed by incubation in permeabilization buffer (PBS containing 0.5% Triton X-100). After 30 min of incubation in permeabilization buffer, organoids were washed once with wash buffer (PBS containing 0.2% Triton X-100, 0.05% Tween 20). Organoids were then blocked for an hour using a blocking buffer (wash buffer supplemented with 1% BSA) followed by overnight incubation in Ki67 antibody (1:200; Abcam, ab15580) dissolved in wash buffer at 4°C. The next day, organoids were washed three times for 5 min using wash buffer. Organoids were incubated in the wash buffer containing AF488-tagged CD324 (also E-cadherin; 1:200; Thermo Fisher Scientific), Rhodamine Red-X conjugate (1:300; Jackson ImmunoResearch, 111-295-144), and DAPI. After 1 hour of incubation, organoids were washed three times for 5 min using wash buffer. Coverslips were then mounted on microscopy slides (22034486, Thermo Fisher Scientific) using ProLong Gold Antifade mounting media and left to dry overnight in the dark at room temperature. Samples were visualized using the Zeiss Axio Observer microscope and analyzed using ImageJ. For qRT-PCR, organoids were treated with vehicle control (dimethyl sulfoxide) or MEK inhibitor U0126 (9903, Cell Signaling Technology) as indicated in the respective figure legend and cells were processed for qRT-PCR as described above.

### Detection of active Ras

HEK293T cells were seeded at a density of 1.5 × 10^6^ cells in a 100-mm dish (430167, Corning) and incubated at 37°C. After reaching 80% confluency, cells were either left nontransfected or transfected with an empty vector or vector containing Ku70 plasmid. Forty-eight hours after transfection, activation of Ras was detected using Active Ras Detection Kit (Cell Signaling Technology, 8821) according to the manufacturer’s instructions.

### Processing of snRNA-seq and scRNA-seq data

The raw small nuclear RNA sequencing (snRNA-seq) data for normal tissues, polyps, and colorectal cancer samples (GSE201348) were read into Seurat v4.9 (*112*, *113*) and filtered to remove low-quality cells. Data were normalized using Seurat with variance stabilization for feature selection. Principal components analysis and Uniform Manifold Approximation and Projection (UMAP) methods were used for dimensional reduction and clustering visualization. The FindAllMarkers function was used to find differentially expressed genes across clusters. The stromal and epithelial cell subtypes were labeled on the basis of the expression of well-known marker genes as described previously (*30*). The single-cell RNA sequencing (scRNA-seq) data for healthy, noninflamed, and inflamed samples for Crohn’s disease available at the Broad Single Cell Portal (SCP1884) were processed with Seurat’s standard pipeline (*112*, *113*). Data were log-normalized and scaled in the Seurat. Cells were clustered and plotted by the UMAP method. The cells were classified into three major cell types (epithelial cells, stromal cells, and immune cells), and detailed cell types were labeled on the basis of the original paper (*31*). Data are available at the Broad Single Cell Portal (SCP1884) and via the Gene Expression Omnibus (GSE201348).

### Retrieval and analysis of data from online databases

The expression pattern of nucleic acid sensors in the colon tissue of healthy humans was retrieved from the Genotype-Tissue Expression (GTEx) portal (*114*). Data on the association between the expression of gene encoding Ku70 (*XRCC6*) and survival of patients with colon and rectal adenocarcinoma were accessed from the Human Protein Atlas (*115*). The number of cancer cases with respect to alterations of *XRCC6* and the type of alteration of *XRCC6* was evaluated using the My Cancer Genome portal (*116*). Percentage copy number variation (amplification and deletion) in *XRCC6* from patients with colon and rectal adenocarcinoma was evaluated using the Gene Set Cancer Analysis web server (*117*). The accessed data were redrawn using GraphPad Prism. Co-occurrence and mutual exclusivity of mutations in genes encoding Ku70, Ras, and Raf in patients with colorectal cancer were assessed using cBioPortal (*118*).

### Statistical analyses

The GraphPad Prism v9.0 or PRIMER-e v6 software was used for data analyses. Data are presented as means ± SEM. Statistical significance was determined by the unpaired *t* test when comparing two groups at a single time point. One-way analysis of variance (ANOVA) with Tukey’s post hoc test was used when comparing three groups at a single time point. Two-way ANOVA with Holm-Šídák post hoc test was used when comparing two or more groups at multiple time points. Statistical significance for 16*S* rRNA data was determined by two-factor analysis of similarities (ANOSIM) when comparing two groups at two time points, whereas one-factor ANOSIM was used when comparing two groups at a single time point. Statistical significance was defined as NS, not statistically significant, and *P* > 0.05; *P < 0.05; **P < 0.01; ***P < 0.001; and ****P < 0.0001. Details of the statistical tests are provided in the figure legends where applicable. No statistical methods were used to predetermine the sample size, and experiments were not randomized. Investigators were blinded to the genotype of the mice and/or treatment in certain experiments. The information on blinding is included in the relevant method sections.
