# Supplementary material for: Ku70 senses cytosolic DNA and assembles a tumor-suppressive signalosome
Source: Sci Adv. 2024 Jan 26;10(4):eadh3409. doi: 10.1126/sciadv.adh3409 (PMC10816715; doi:10.1126/sciadv.adh3409)
Supplement: Supplementary file 1 — Figs. S1 to S22 Tables S1 to S3 [file sciadv.adh3409_sm.pdf]

Science Advances
AAAS

# Supplementary Materials for

## Ku70 senses cytosolic DNA and assembles a tumor-suppressive signalosome

Abhimanu Pandey *et al.*

Corresponding author: Si Ming Man, siming.man@anu.edu.au

*Sci. Adv.* **10**, eadh3409 (2024)
DOI: 10.1126/sciadv.adh3409

**This PDF file includes:**

Figs. S1 to S22
Tables S1 to S3

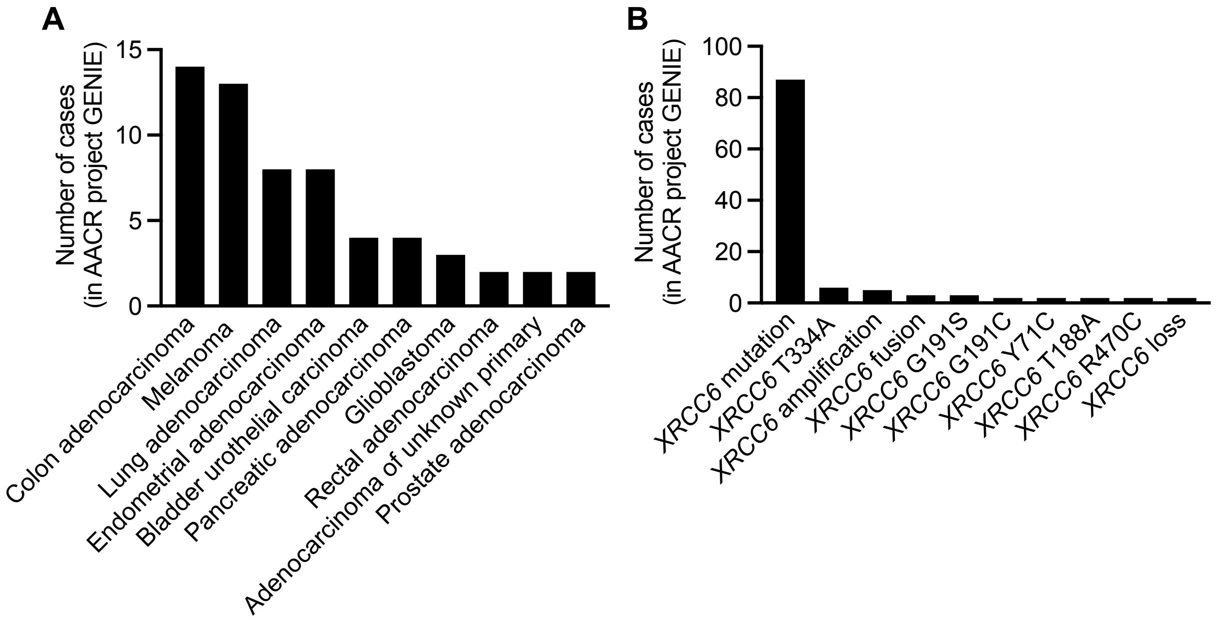


**Fig. S1. Changes in the gene encoding Ku70 are associated with colorectal cancer in humans.** (**A**) Number of cancer cases with respect to alterations in gene encoding Ku70 (*XRCC6*), determined using The American Association for Cancer Research (AACR) project Genomics Evidence Neoplasia Information Exchange (GENIE) from My Cancer Genome portal. (**B**) Type of alteration in *XRCC6* determined using AACR project GENIE from My Cancer Genome portal.

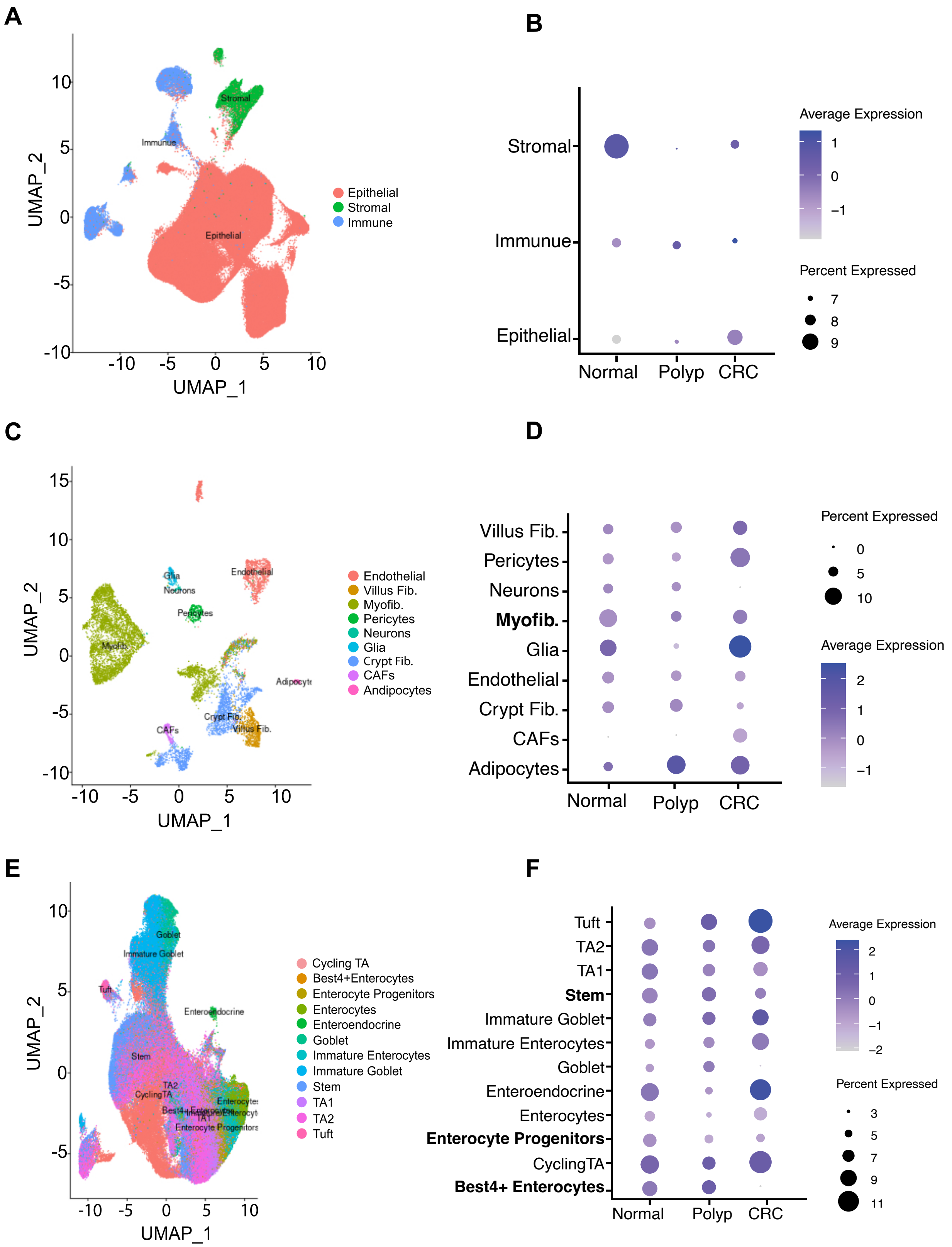


**Fig. S2. The expression of the gene encoding Ku70 changes in a cell type-specific manner during the development of colorectal cancer in humans.** (**A** and **B**) UMAP representation of

snRNA-seq data (A) and average expression of the gene encoding Ku70 in cells of stromal, immune, and epithelial compartments obtained from normal, polyp, and colorectal cancer samples (B). (**C** and **D**). UMAP representation of snRNA-seq data (C) and average expression of the gene encoding Ku70 in cells of stromal compartment obtained from normal, polyp, and colorectal cancer samples (D). (**E** and **F**). UMAP representation of snRNA-seq data (E) and average expression of the gene encoding Ku70 in cells of the epithelial compartment obtained from normal, polyp, and colorectal cancer samples (F). The size of the dots denotes the fraction of each cell type expressing the gene encoding Ku70, whereas, color bars denote the average expression of the gene encoding Ku70 in indicated cells. Data are available from the Gene Expression Omnibus (GSE201348).

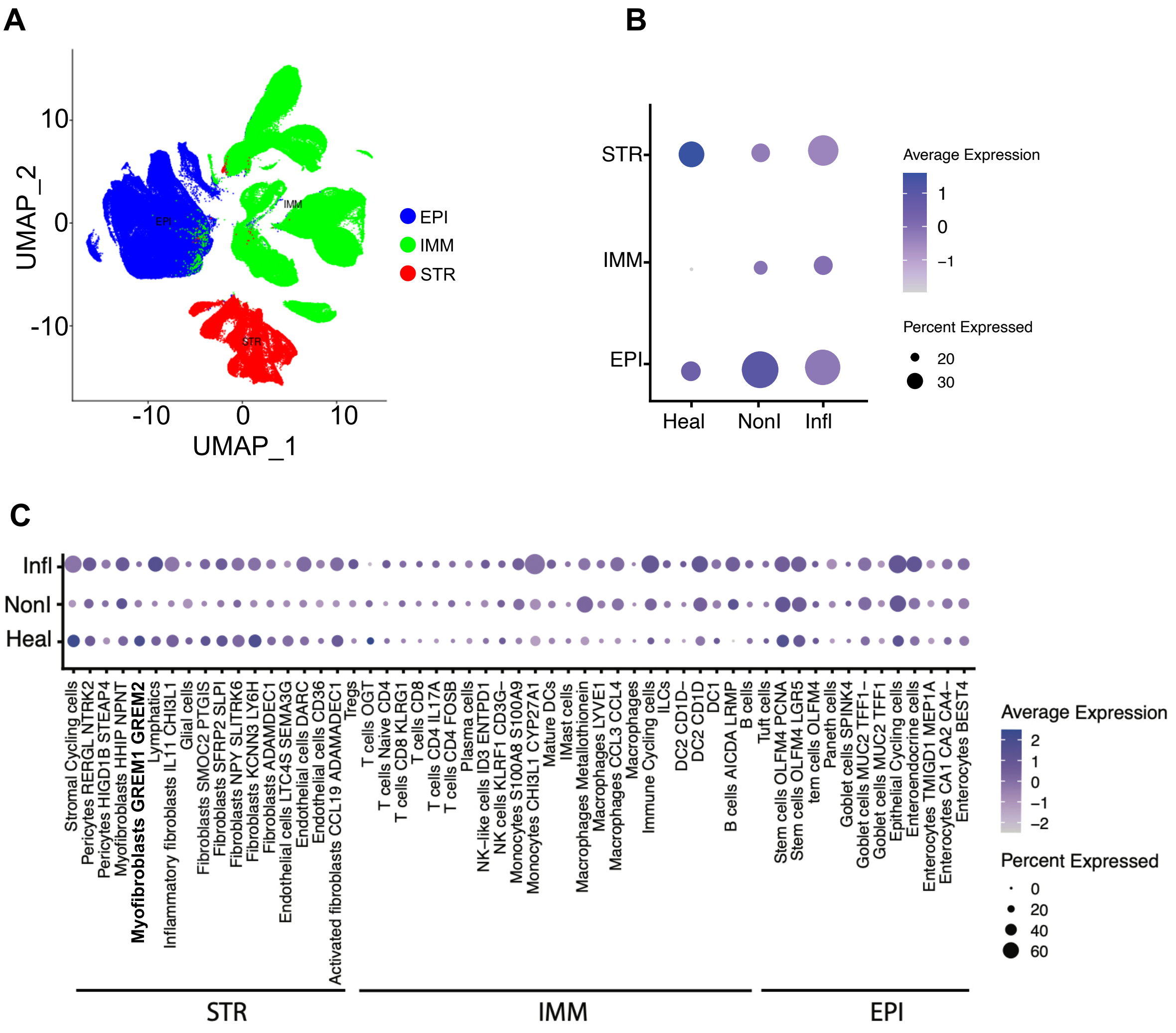


**Fig. S3. The expression of the gene encoding Ku70 changes in a cell type-specific manner during the development of Crohn's disease.** (**A** to **C**) UMAP representation of scRNA-seq data (A) and average expression of the gene encoding Ku70 in stromal, immune, and epithelial compartments (B) and different cell types of stromal, immune, and epithelial compartments (C) obtained from healthy, non-inflamed, and inflamed samples from patients with Crohn's disease. The size of the dots denotes the fraction of each cell type expressing the gene encoding Ku70, whereas, color bars denote the average expression of the gene encoding Ku70 in indicated cells. Data are available from the Broad Single Cell Portal (SCP1884). STR: Stromal cells; EPI: Epithelial cells; IMM: Immune cells; Heal: Healthy samples; NonI: Non-inflamed samples; Infl: Inflamed samples.

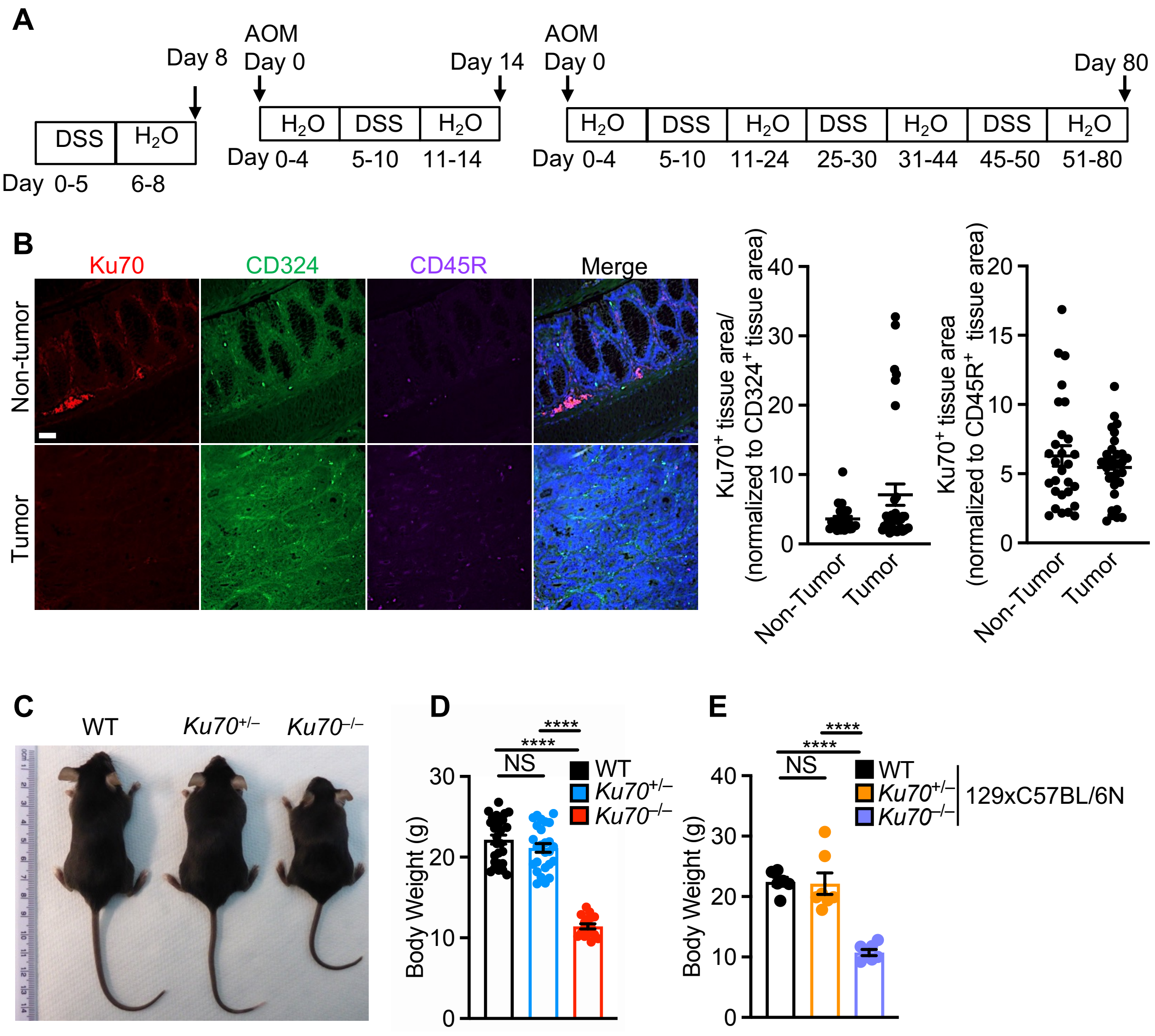


**Fig. S4.** ***Ku70*$^{-/-}$ mice are growth retarded as compared to littermate WT and *Ku70*$^{+/-}$ mice.** (**A**) Timeline of dextran sulfate sodium (DSS) treatment (left) and short-term azoxymethane (AOM) and DSS treatment (middle), and long-term AOM-DSS treatment (right) in mice. (**B**) Representative images of immunohistochemical staining of Ku70, CD324, CD45R, and DAPI (left) and quantification of Ku70-positive area in CD324- and CD45R-positive tissue (right) in the colon tissue of WT mice 80 days after AOM-DSS treatment. Scale bar, 50μm. (**C**) Representative images of littermate WT, *Ku70*$^{+/-}$ and *Ku70*$^{-/-}$ mice on the 129 genetic background at 8 weeks of age. (**D**) Body weight of mice as shown in (C). (**E**) Body weight of littermate WT, *Ku70*$^{+/-}$ and *Ku70*$^{-/-}$ mice on the 129 background and backcrossed to the C57BL/6N genetic background for three generations, at 8 weeks of age. Each symbol represents an individual area of the tissue in (B) or an individual mouse in (D and E). NS, not statistically significant; ****$P$<0.0001 by one-way ANOVA Tukey's multiple comparisons test (D and E). Data are presented as mean ± s.e.m. in (B, D and E).

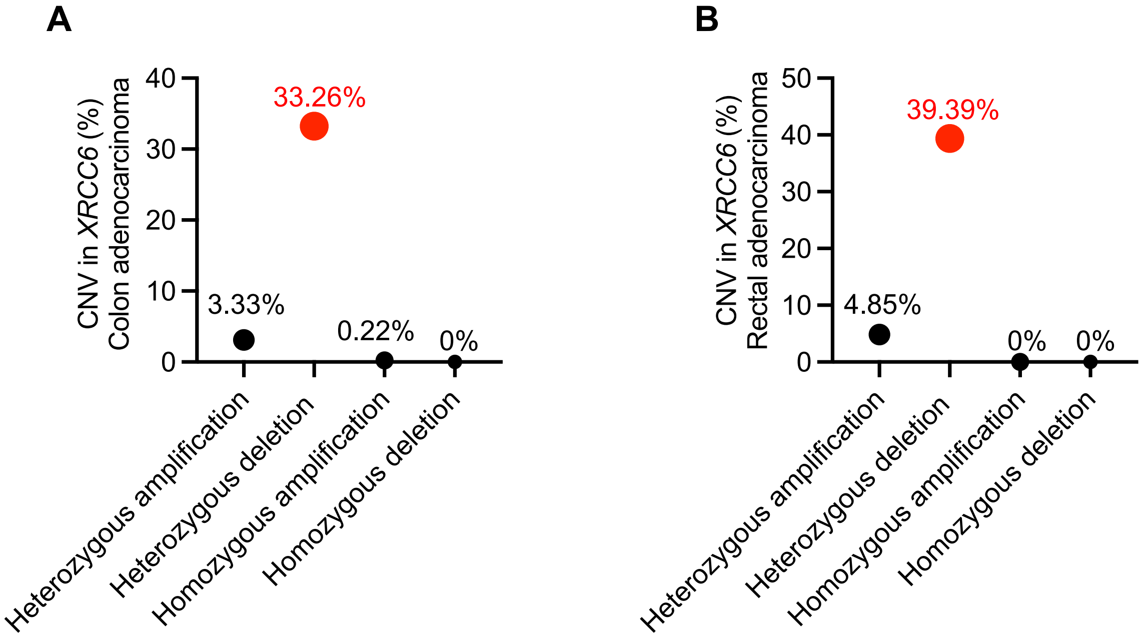


**Fig. S5. Heterozygous deletion of XRCC6 is commonly found in tumors of patients with colon and rectal adenocarcinoma.** (**A** and **B**) Percentage of copy number variation (CNV; amplification and/or deletion) in *XRCC6* in colon adenocarcinoma (A) and rectal adenocarcinoma (B), determined using Gene Set Cancer Analysis web server.

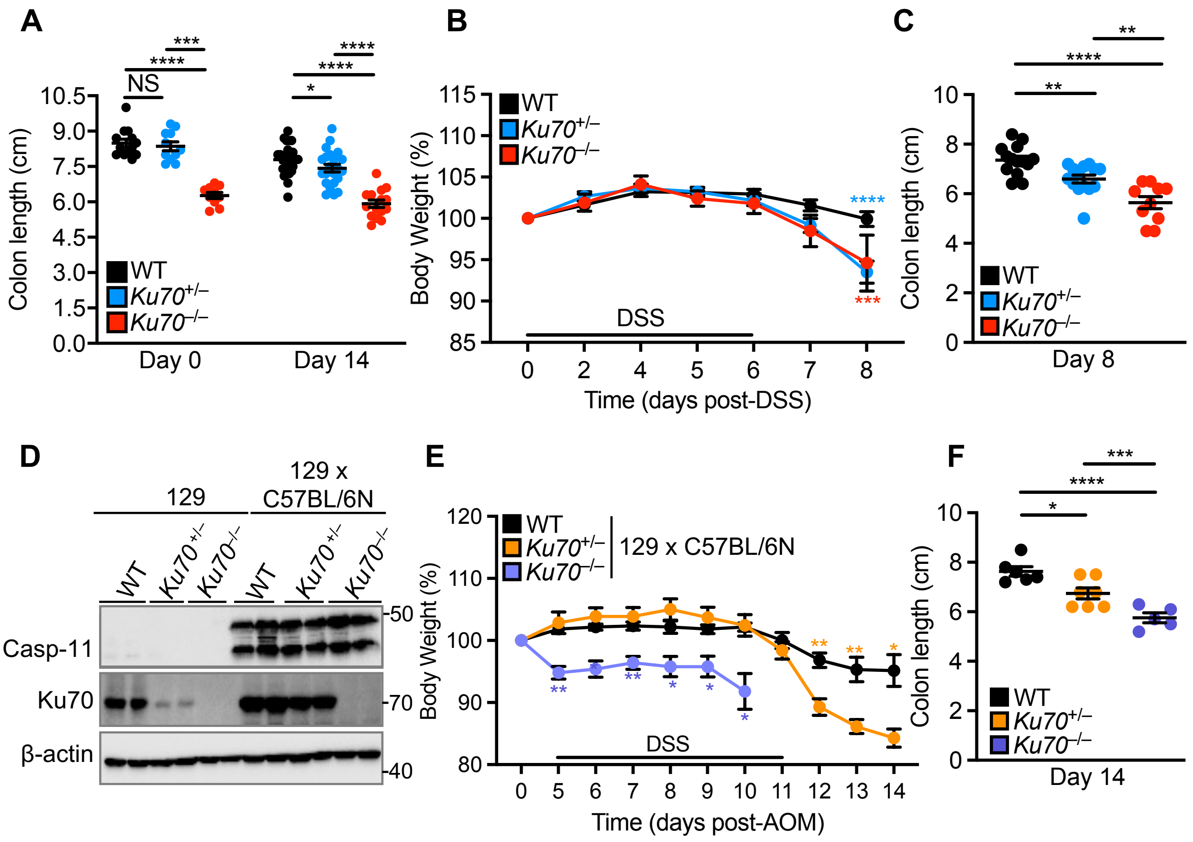


**Fig. S6. Ku70 attenuates the development of colitis.** (**A**) Colon length of untreated mice on day 0 and 14 days AOM-DSS treated mice on the 129 genetic background. (**B**) Percentage change in body weight of littermate WT (n=14), *Ku70*$^{+/-}$ (n=14), and *Ku70*$^{-/-}$ (n=10) mice on the 129 genetic background during DSS treatment. (**C**) Colon length of mice 8 days after DSS treatment. (**D**) Immunoblot of indicated proteins in the colon tissue of mice. (**E**) Percentage change in body weight of littermate WT (n=6), *Ku70*$^{+/-}$ (n=7), and *Ku70*$^{-/-}$ (n=5) mice on the 129 background and backcrossed to the C57BL/6N genetic background for three generations, during AOM-DSS treatment. (**F**) Colon length of mice 14 days after AOM injection. Each symbol (A, C, and F) or lane (D) represents an individual mouse. NS, not statistically significant; **P*<0.05; ***P*<0.01; ****P*<0.001; *****P*<0.0001; by two-way ANOVA with Holm-Šídák's multiple comparisons test in (A, B and E) or one-way ANOVA with Tukey's multiple comparisons test in (C and F). Data are pooled from three independent experiments in (A to C) or two independent experiments in (E and F) and are presented as mean ± s.e.m. in (A to C, E and F).

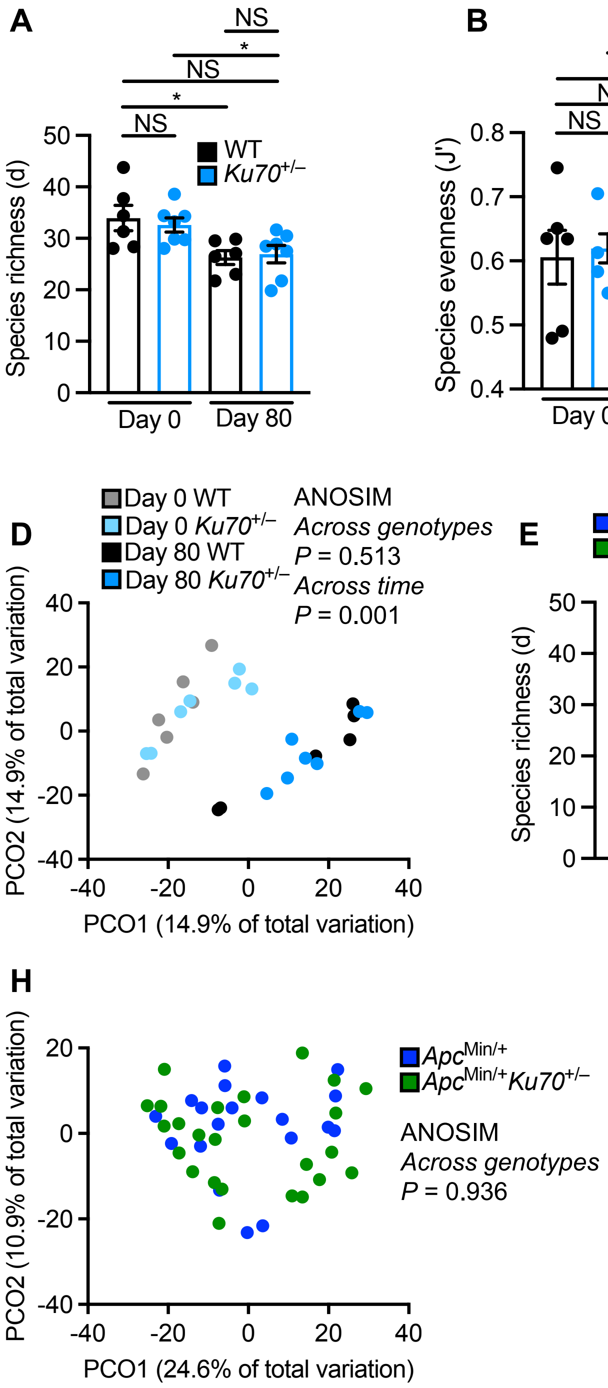


**Fig. S7. The gut microbiota composition is similar between littermate-controlled WT and *Ku70*$^{+/-}$, and *Apc*$^{Min/+}$ and *Apc*$^{Min/+}$ *Ku70*$^{+/-}$ mice.** (**A** to **D**) Species richness (A), species evenness (B), and Shannon's diversity (C) of bacterial composition and principal-coordinate (PCO) analysis of a Bray-Curtis resemblance matrix, generated from square-root-transformed relative abundances of bacterial operational taxonomic units (OTUs) (D) in the fecal pellets from the colon of untreated (day 0) and AOM-DSS treated (day 80) littermate WT and *Ku70*$^{+/-}$ mice. (**E** to **H**) Species richness (E), species evenness (F), and Shannon's diversity (G) of bacterial composition and PCO analysis of a Bray-Curtis resemblance matrix, generated from square-root-transformed relative abundances

of bacterial OTUs (H) in the fecal pellets from the colon of 20-weeks-old littermate *Apc*$^{Min/+}$ and *Apc*$^{Min/+}$*Ku70*$^{+/-}$ mice. Each symbol represents an individual mouse (A to H). NS, not statistically significant; **P*<0.05 by unpaired *t*-test (A to C and E to G) or by two-factor Analysis of Similarities (ANOSIM) in (D and H). *P* values are indicated in (D and H). Data are presented as mean ± s.e.m. in (A to C and E to G).

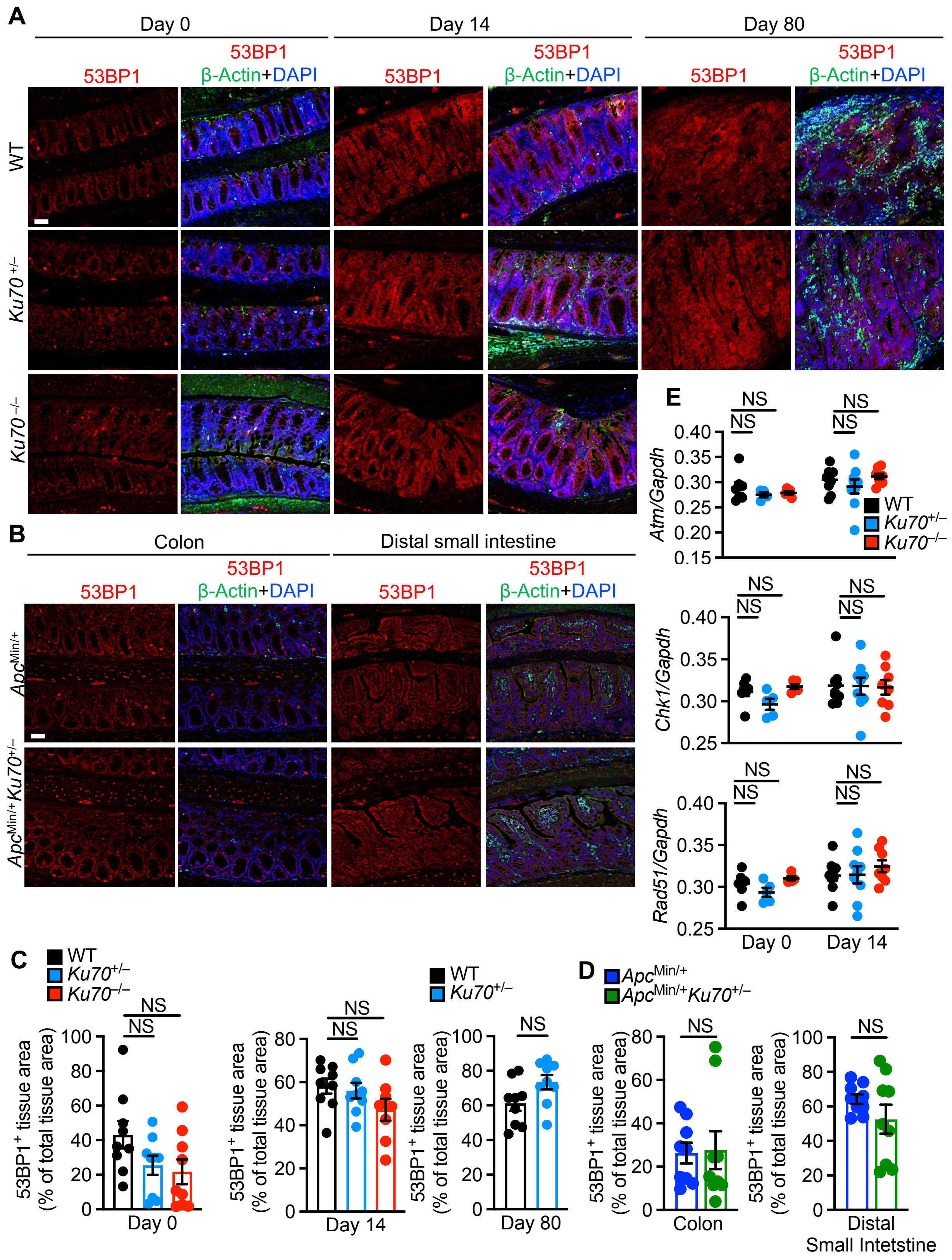


**Fig. S8. Ku70 does not affect DNA damage during the development of colitis.** (**A** and **B**) Immunohistochemical staining of 53BP1, β-actin, and DAPI in the colon tissue of untreated littermate WT, *Ku70*$^{+/-}$ and *Ku70*$^{-/-}$ mice on the 129 genetic background on day 0 (top left), 14

days (top middle) or 80 days (top right) after AOM-DSS treatment or in the colon tissue (bottom left) and distal small intestinal tissue (bottom right) of *Apc*$^{Min/+}$ and *Apc*$^{Min/+}$ *Ku70*$^{+/-}$ mice. Scale bars, 50μm. (**C** and **D**) Quantification of the 53BP1-positive area over total colon tissue area as in (A and B). (**E**) Expression of genes encoding ATM (top), CHK1 (middle), and Rad51 (bottom) in the colon tissue of littermate WT, *Ku70*$^{+/-}$ and *Ku70*$^{-/-}$ mice at day 0 and day 14. Each symbol represents one of the three (proximal, middle, or distal) parts of the colon in (C and D, left panel) or one of three (outer, middle, or inner) parts of the distal small intestine in (D, right panel) or an individual mouse in (E). NS, not statistically significant by one-way ANOVA with Tukey's multiple comparisons test (C, left and middle panels) or unpaired *t*-test (C, right panel and D). Data are presented as mean ± s.e.m. in (C to E).

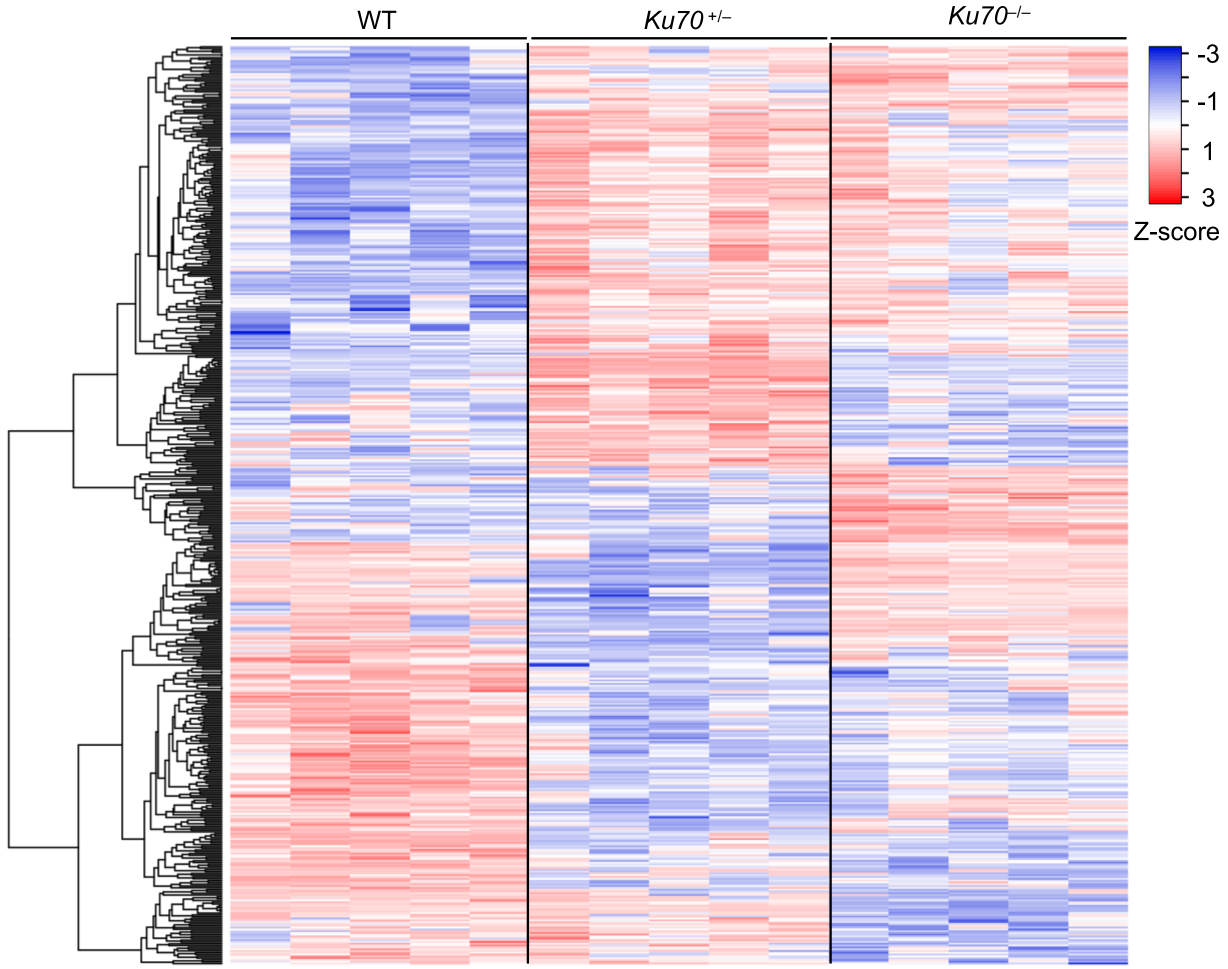


**Fig. S9. Ku70 affects the phosphorylation status of proteins in the colon tissue of mice with colitis.** Heatmap showing differentially phosphorylated proteins after global phospho-proteomic screen on the colon tissue lysate of littermate WT (n=5), *Ku70*$^{+/-}$ (n=5), and *Ku70*$^{-/-}$ (n=5) mice on the 129 genetic background 14 days after AOM injection. Heatmap was created using the Heatmapper web server, the Average linkage method was used for clustering (see Table S1 for the number of clusters), and the Pearson method was used for the distance measurement.

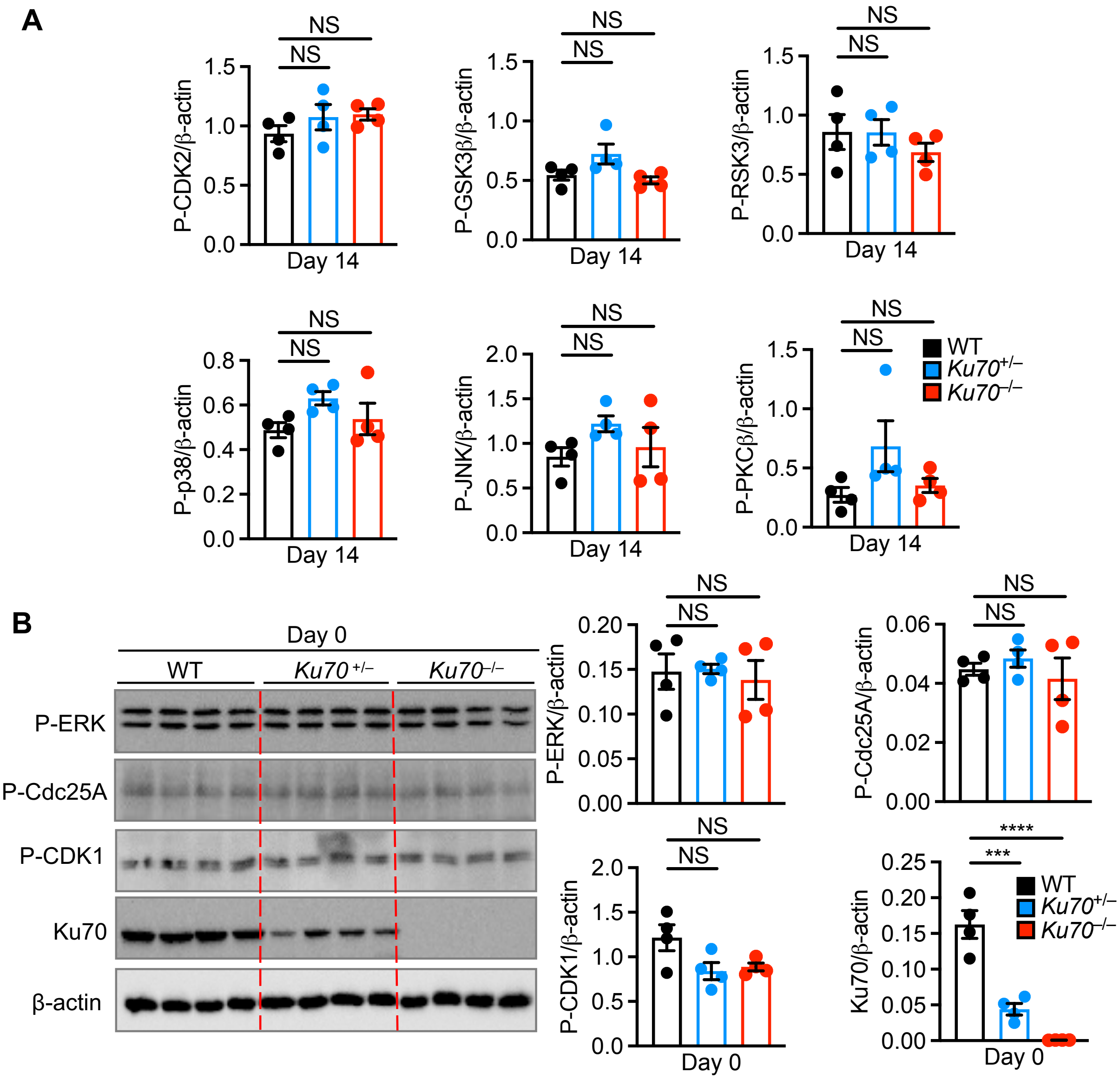


**Fig. S10. Ku70 preferentially activates the ERK signaling pathway during the development of colitis.** (**A**) Densitometric quantification of immunoblots of indicated proteins from the colon tissue lysate of mice at day 14 as shown in Fig. 4C. (**B**) Immunoblot of the indicated proteins (left) and densitometric quantification (right) on the colon tissue lysate mice on the 129 genetic background at day 0. Each symbol (A and B) and lane (B) represents an individual mouse. P-indicates phosphorylated protein in (B). Data are representative of three independent experiments in (A and B) and are presented as mean ± s.e.m. in (A and B).

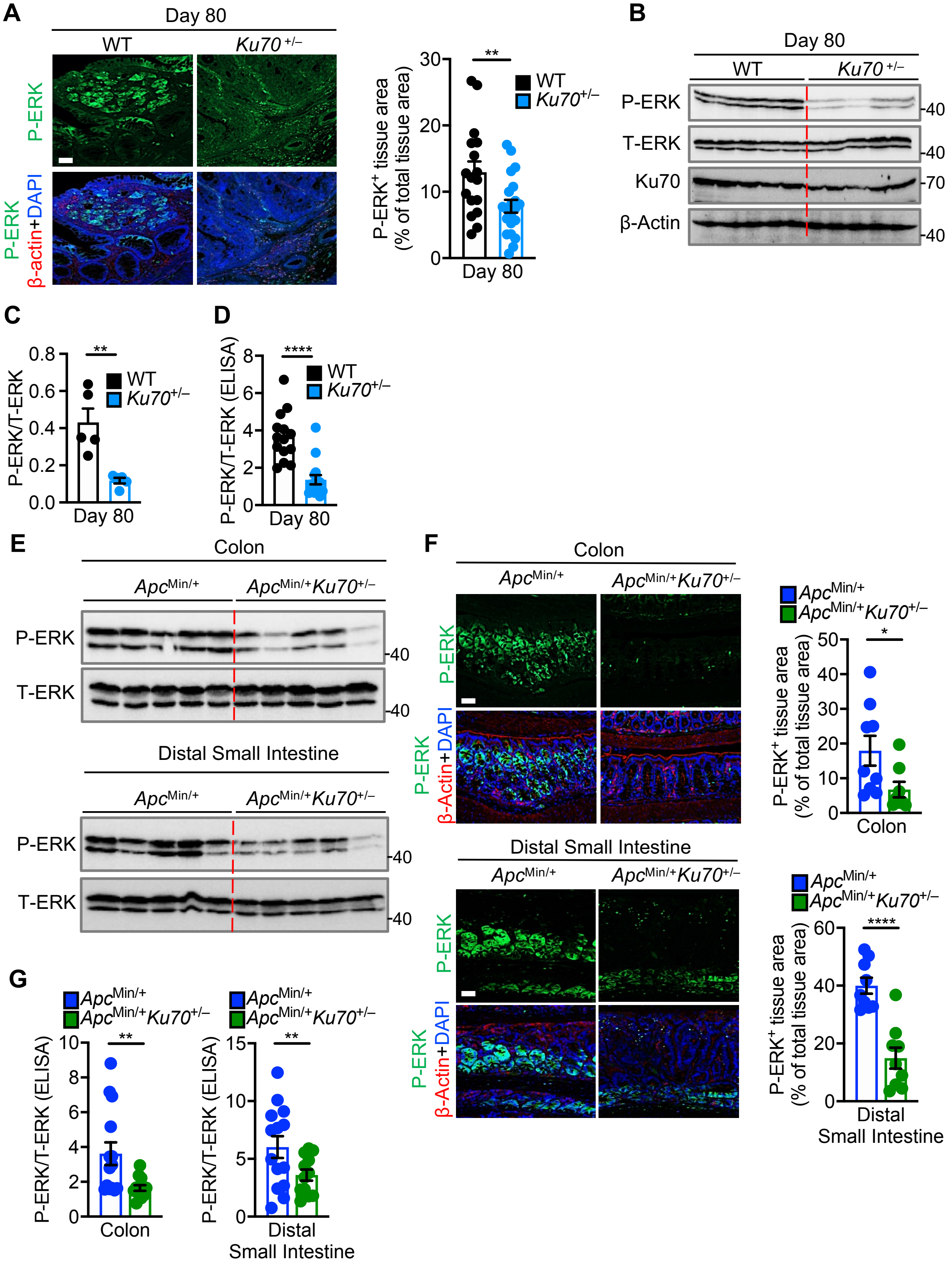


**Fig. S11. Ku70 activates the ERK-signaling pathway during the development of intestinal cancer.** (**A**) Immunohistochemical staining of P-ERK, β-actin, and DAPI (left) and quantification of P-ERK-positive area over total tissue area (right) in the colon tissue of mice 80 days after AOM injection. Scale bar, 50μm. (**B**) Immunoblot of the indicated proteins on the colon tissue lysate of

littermate WT and *Ku70*$^{+/-}$ mice. (**C**) Densitometric quantification of immunoblot of indicated proteins on the colon tissue lysate of mice as shown in (B). (**D**) The ratio of P-ERK over T-ERK by ELISA on the colon tissue lysate of mice 80 days after AOM injection. (**E**) Immunoblot of the indicated proteins in the colon tissue (top) and distal small intestinal tissue (bottom) of littermate *Apc*$^{\mathrm{Min/+}}$ and *Apc*$^{\mathrm{Min/+}}$ *Ku70*$^{+/-}$ mice at 20 weeks of age. (**F**) Immunohistochemical staining of P-ERK, β-actin and DAPI in the colon tissue (top left) and distal small intestinal tissue (bottom left), and quantification of P-ERK-positive area over total colon tissue area (top right), and P-ERK-positive area over total distal small intestinal tissue area (bottom right) of littermate *Apc*$^{\mathrm{Min/+}}$ and *Apc*$^{\mathrm{Min/+}}$ *Ku70*$^{+/-}$ mice. Scale bar, 50μm. (**G**) The ratio of P-ERK over T-ERK by ELISA on the colon tissue lysate (left) and distal small intestinal tissue lysate (right) of littermate *Apc*$^{\mathrm{Min/+}}$ and *Apc*$^{\mathrm{Min/+}}$ *Ku70*$^{+/-}$ mice. Each symbol represents one of the three (proximal, middle, or distal) parts of the mouse colon in (A and F, top panel) or one of three (outer, middle, or inner) parts of the distal small intestine in (F, bottom panel). Each lane (B and E) and symbol (C, D, and G) represents an individual mouse. P- indicates phosphorylated (A to G) and T- indicates total protein in (B to E and G). *$P<0.05$; **$P<0.01$; ****$P<0.0001$; by unpaired *t*-test (A, C, D, F and G). Data are representative of (A to C, E and F) or pooled from three independent experiments in (D) and are presented as mean ± s.e.m. in (A, C, D, F and G).

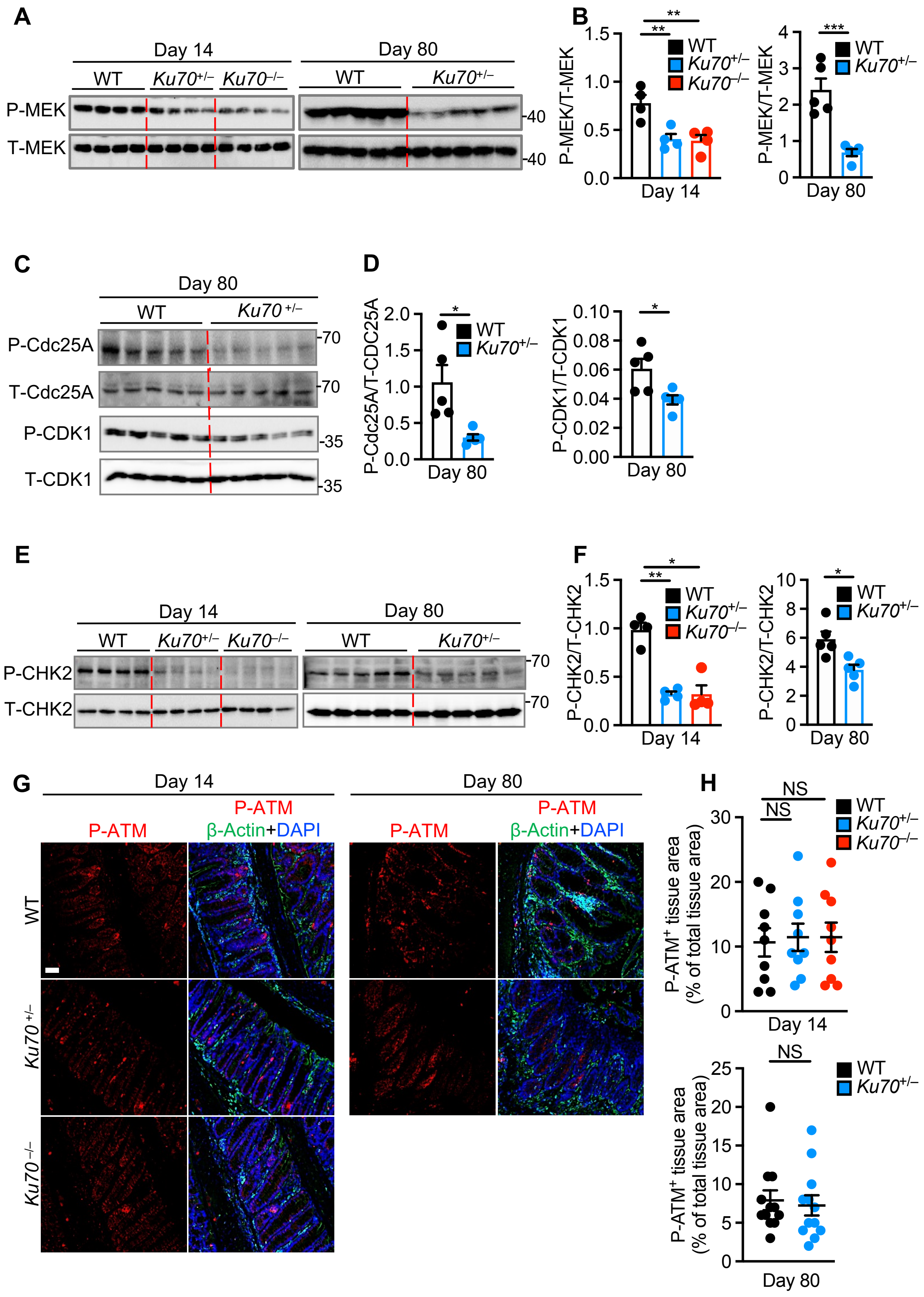
A
Day 14
Day 80
WT
Ku70+/–
Ku70–/–
P-MEK
T-MEK
-40
B
P-MEK/T-MEK
Day 14
Day 80
C
P-Cdc25A
T-Cdc25A
P-CDK1
T-CDK1
-70
-35
D
P-Cdc25A/T-CDC25A
P-CDK1/T-CDK1
E
P-CHK2
T-CHK2
F
P-CHK2/T-CHK2
G
P-ATM
β-Actin+DAPI
H
NS
P-ATM+ tissue area
(% of total tissue area)

**Fig. S12. Ku70 phosphorylates MEK during the development of colitis and colitis-associated colorectal cancer.** (**A**) Immunoblot of the indicated proteins on the colon tissue lysate of littermate WT, *Ku70*$^{+/–}$ and *Ku70*$^{–/–}$ mice at day 14 (left) and on the colon tissue lysate of littermate WT and *Ku70*$^{+/–}$ mice at day 80 (right). (**B**) Densitometric quantification of the blots as shown in (A). (**C** and **D**) Immunoblot of the indicated proteins (**C**) and densitometric quantification (**D**) on the colon tissue lysate of mice. (**E**) Immunoblot of the indicated proteins on the colon tissue lysate of littermate WT, *Ku70*$^{+/–}$ and *Ku70*$^{–/–}$ mice at day 14 (left) and on the colon tissue lysate of littermate WT and *Ku70*$^{+/–}$ mice at day 80 (right). (**F**) Densitometric quantification of the blots as shown in (E). (**G**) Immunohistochemical staining of P-ATM, β-actin, and DAPI in the colon tissue of untreated littermate WT, *Ku70*$^{+/–}$ and *Ku70*$^{–/–}$ mice on the 129 genetic background on day 14 (left) or day 80 (right) after AOM-DSS treatment. Scale bar, 50μm. (**H**) Quantification of the P-ATM-positive area over total colon tissue area as in (G). Each lane (A, C and E) or symbol (B, D and F) represents an individual mouse or one of the three (proximal, middle, or distal) parts of the colon in (H). P- indicates phosphorylated protein and T- indicates total protein in (A to F). NS, not statistically significant; **P*<0.05; ***P*<0.01 ****P*<0.001; by one-way ANOVA with Tukey's multiple comparisons test (B and F, left panels and H top panel) or unpaired *t*-test (B, right panel, D and F, right panel and H bottom panel). Data are representative of three independent experiments and are presented as mean ± s.e.m. in (B, D, F and H).

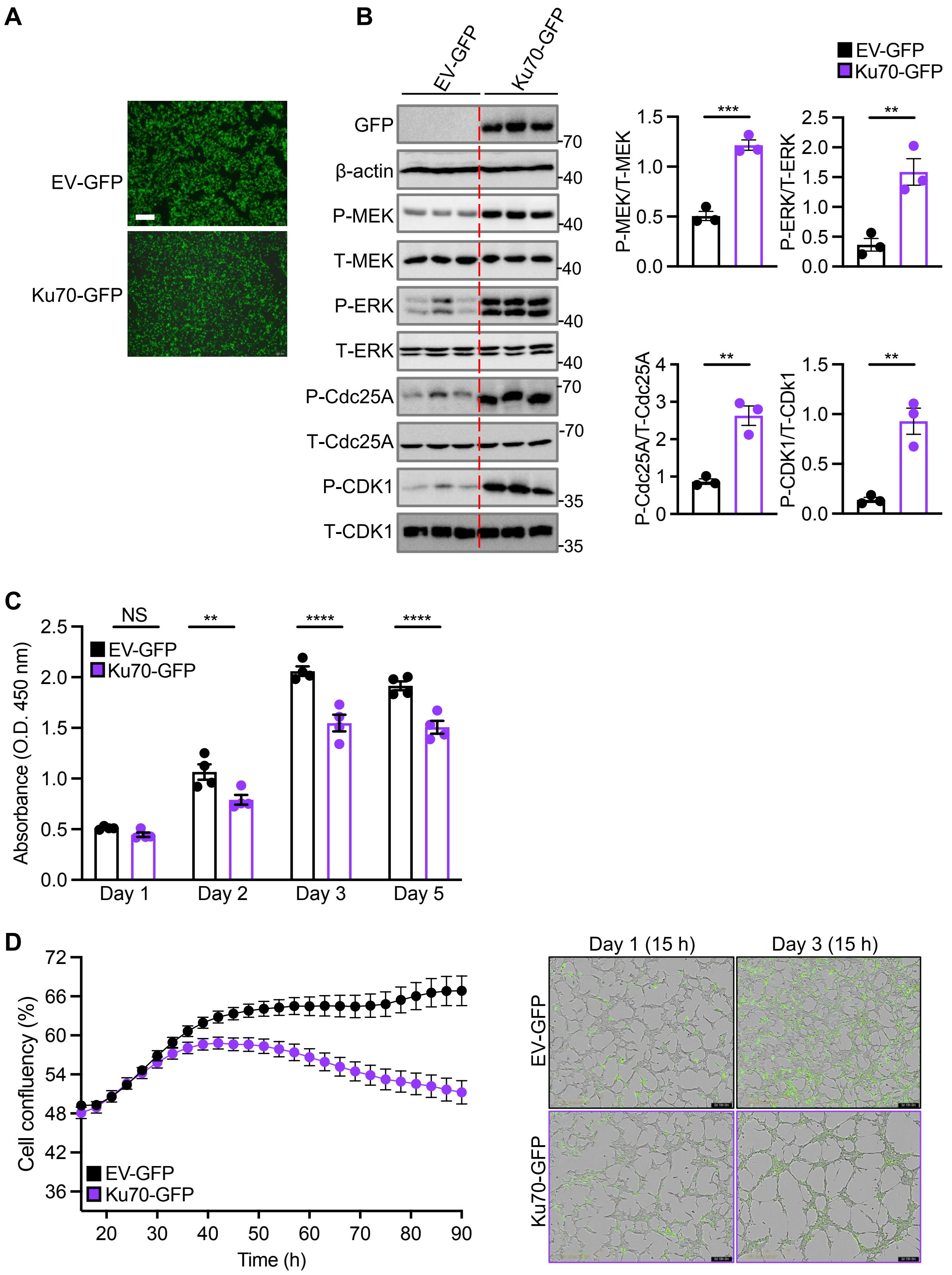


**Fig. S13. Ku70-mediated MEK-ERK signaling restricts cell proliferation.** (**A**) Fluorescence microscopy images of HEK293T cells captured 24 hours after transfection with the empty vector

(EV) or Ku70 plasmid. Scale bar, 300μm. (**B**) Immunoblot of the indicated proteins (left) and densitometric quantification (right) on the cell lysate of HEK293T cells transfected with the EV or Ku70 plasmid. (**C**) Proliferation of HEK293T cells transfected with the EV or Ku70 plasmid at the indicated time points, determined by WST-1 assay. (**D**) IncuCyte live imaging analysis of the cell confluency (left) and representative images of HEK293T cells transfected with an EV-GFP or Ku70-GFP plasmid at the indicated time. P- indicates phosphorylated protein and T- indicates total protein in (B). Each lane or symbol represents one independent experiment in (B). Data are representative of two experiments in (C and D). NS, not statistically significant; **$P$<0.01; ***$P$<0.001; ****$P$<0.0001; by unpaired $t$-test in (B and C). Data are presented as mean ± s.e.m. in (B and C) or mean ± S.D in (D).

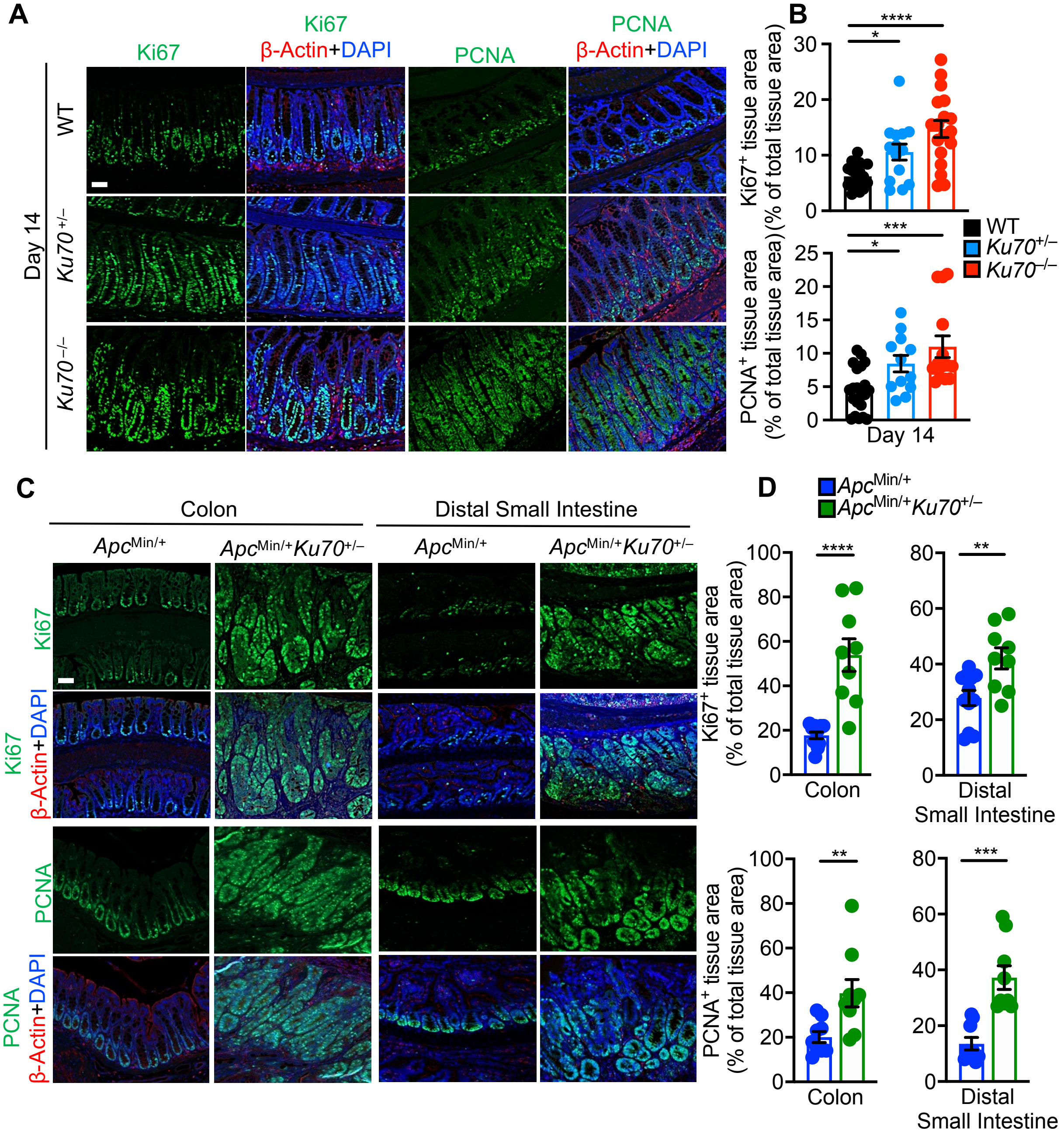


**Fig. S14. Ku70 restricts overt cell proliferation during the development of colitis and intestinal cancer.** (**A**) Representative images of immunohistochemical staining of Ki67, β-actin, and DAPI (left) and PCNA, β-actin, and DAPI (right) in the colon tissue of littermate WT, *Ku70*$^{+/–}$ and *Ku70*$^{–/–}$ mice. Scale bar, 50μm. (**B**) Quantification of Ki67 (top) and PCNA (bottom) positive area over total tissue area. (**C**) Representative images of immunohistochemical staining of Ki67, β-actin, and DAPI (top left) and PCNA, β-actin, and DAPI (bottom left) in the colon, and Ki67, β-actin, and DAPI (top right) and PCNA, β-actin, and DAPI (bottom right) in the distal small intestine of 20 weeks old littermate *Apc*$^{Min/+}$ and *Apc*$^{Min/+}$*Ku70*$^{+/–}$ mice. Scale bar, 50μm. (**D**) Quantification of Ki67 (top left) and PCNA (bottom left) in the colon, and Ki67 (top right) and PCNA (bottom right) in the distal small intestine of 20 weeks old littermate *Apc*$^{Min/+}$ and *Apc*$^{Min/+}$*Ku70*$^{+/–}$ mice. Each symbol represents one of the three (proximal, middle, or distal) parts

of the colon or one of three (outer, middle, or inner) parts of the distal small intestine in (A to D). $^{*}P<0.05$; $^{**}P<0.01$; $^{***}P<0.001$; $^{****}P<0.0001$ by one-way ANOVA with Tukey's multiple comparisons test (B) or by unpaired *t*-test (D). Data are pooled from three independent experiments in (B) and are presented as mean ± s.e.m. in (B and D).

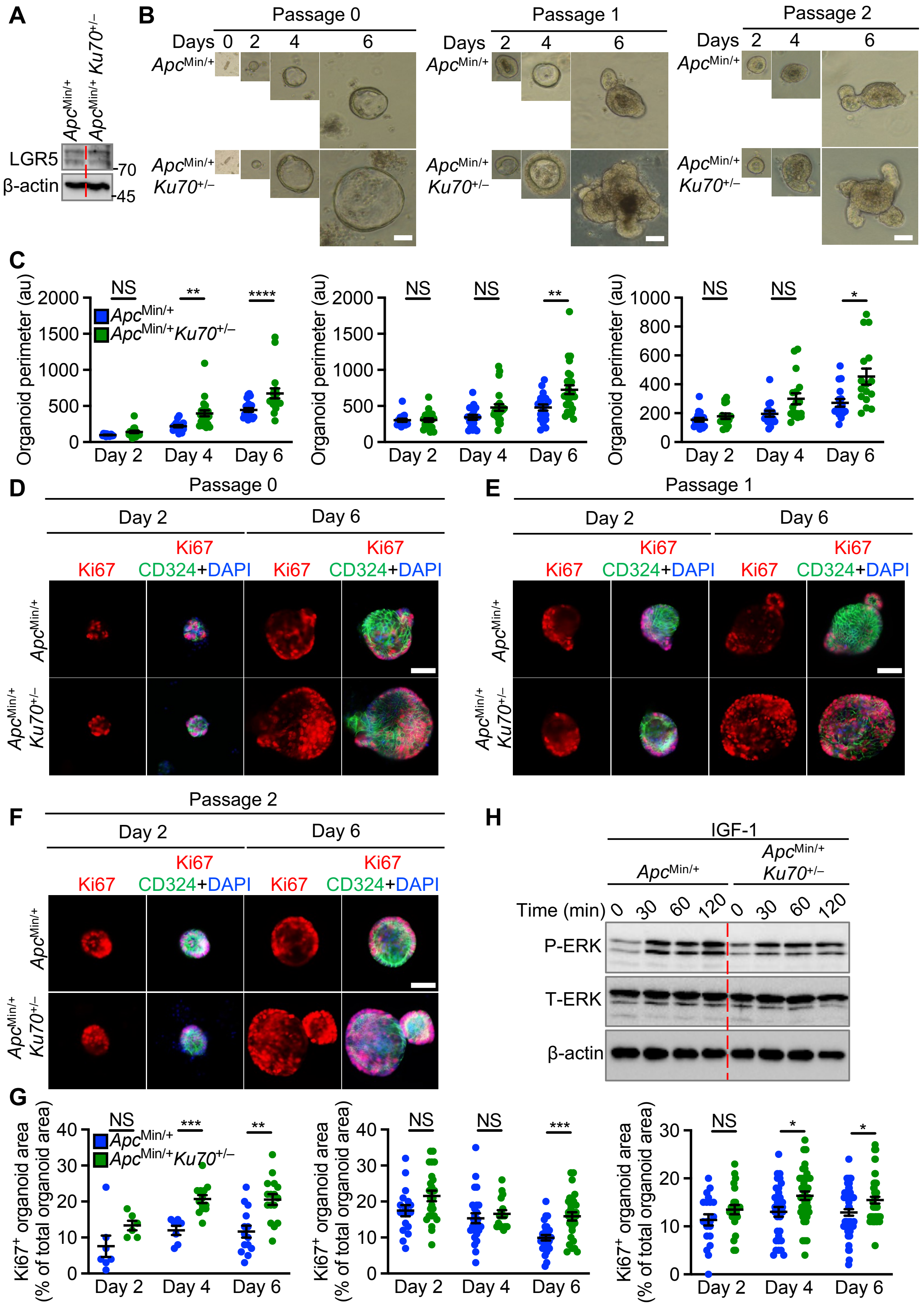
A
B
Passage 0
Passage 1
Passage 2
Days 0 2 4 6
ApcMin/+
ApcMin/+ Ku70+/–
LGR5
β-actin
70
45
C
Organoid perimeter (au)
NS
**
****
*
Day 2 Day 4 Day 6
D
E
F
Day 2
Day 6
Ki67
CD324+DAPI
H
IGF-1
Time (min) 0 30 60 120 0 30 60 120
P-ERK
T-ERK
G
Ki67+ organoid area (% of total organoid area)
***

**Fig. S15. Ku70 may contribute to controlling intestinal stem cell proliferation via ERK signaling.** (**A**) Immunoblot of the indicated proteins on the cell lysates of mouse intestinal crypts isolated from the colon of 10-week-old littermate *Apc*$^{Min/+}$ and *Apc*$^{Min/+}$ *Ku70*$^{+/-}$ mice. (**B**) Images of mouse intestinal organoids cultured from stem cells of the colon of 10-week-old littermate *Apc*$^{Min/+}$ and *Apc*$^{Min/+}$ *Ku70*$^{+/-}$ mice at passage 0 (left), passage 1 (middle), and passage 2 (right). Scale bars, 50μm (**C**) Size (perimeter; au, arbitrary unit) of mouse intestinal organoids as in (B) at passage 0 (left), passage 1 (middle), and passage 2 (right). (**D** to **G**) Immunofluorescence staining of Ki67, CD324 (E-Cadherin) and DAPI in mouse intestinal organoids as in (B), at passage 0 (D), passage 1 (E), and passage 2 (F); and quantification (G) of Ki67-positive organoid area over the total organoid area at passage 0 (left), passage 1 (middle), and passage 2 (right). Scale bars, 50μm. (**H**) Immunoblot of the indicated proteins on the lysates of mouse intestinal organoids left untreated (time 0) or treated with insulin growth factor (IGF)-1 (50ng/ml) for the indicated time. Each symbol represents an individual organoid in (C and G). **P*<0.05; ***P*<0.01; ****P*<0.001; *****P*<0.0001 by two-way ANOVA with Šídák's multiple comparisons test (C and G). Data are representative from two independent experiments in (A to H) and are presented as mean ± s.e.m. in (C and G).

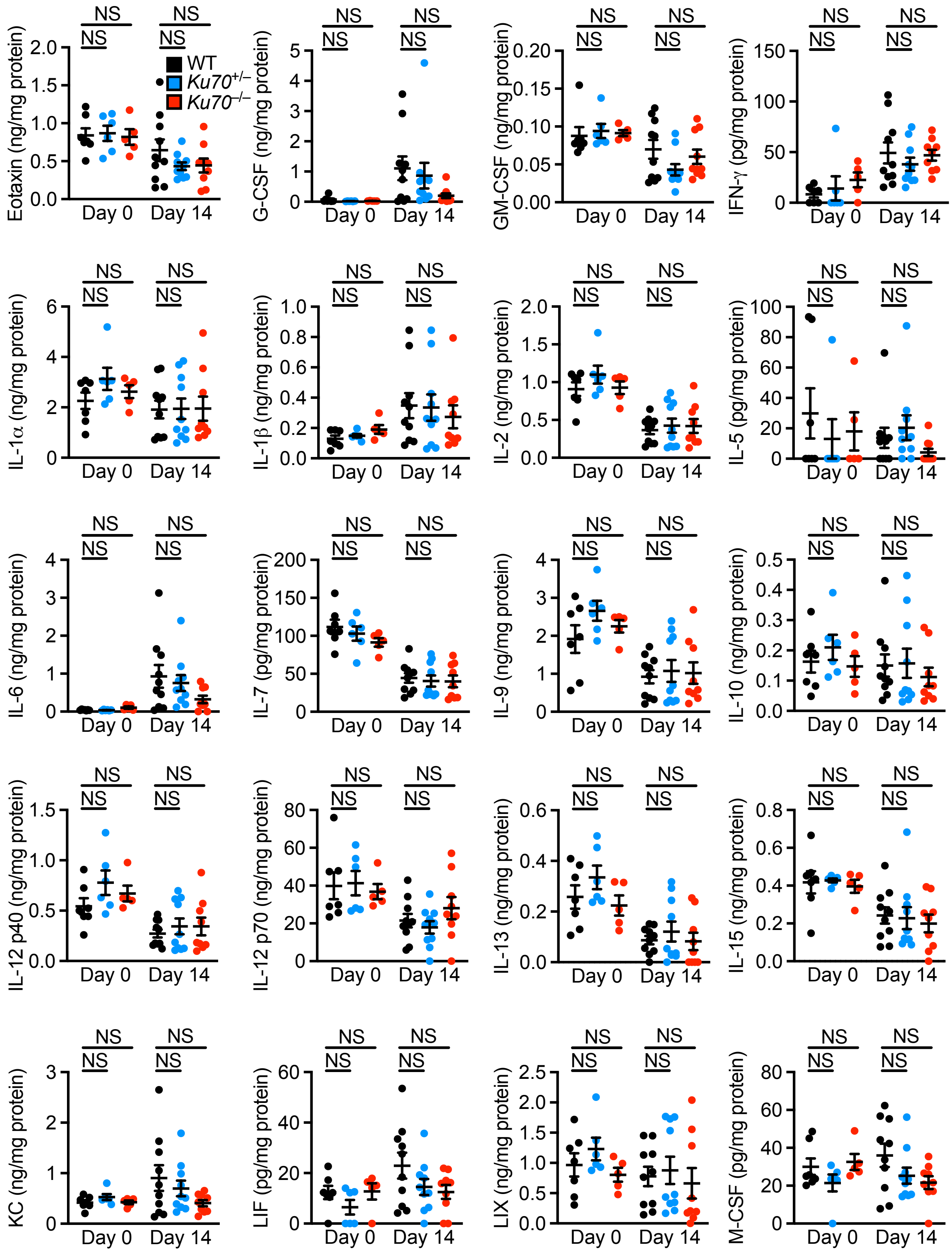


**Fig. S16. Ku70 does not induce or suppress the production of inflammatory mediators during the development of colitis.** Levels of eotaxin, granulocyte colony-stimulating factor (G-CSF), granulocyte-macrophage colony-stimulating factor (GM-CSF), interferon (IFN-γ), interleukin (IL)

1α, IL-β, IL-2, IL-5, IL-6, IL-7, IL-9, IL-10, IL-12p40, IL-12p70, IL-13, IL-15, keratinocytes-derived chemokine (KC), leukemia inhibitory factor (LIF), C-X-C motif chemokine 5 (LIX) and macrophage colony-stimulating factor (M-CSF) in the colon tissue of untreated (day 0) and AOM-DSS treated (day 14) littermate WT, *Ku70*$^{+/-}$ and *Ku70*$^{-/-}$ mice, determined using multiplex ELISA. Each symbol represents an individual mouse. NS, not statistically significant by one-way ANOVA with Tukey's multiple comparisons test. Data are pooled from three independent experiments and are presented as mean ± s.e.m.

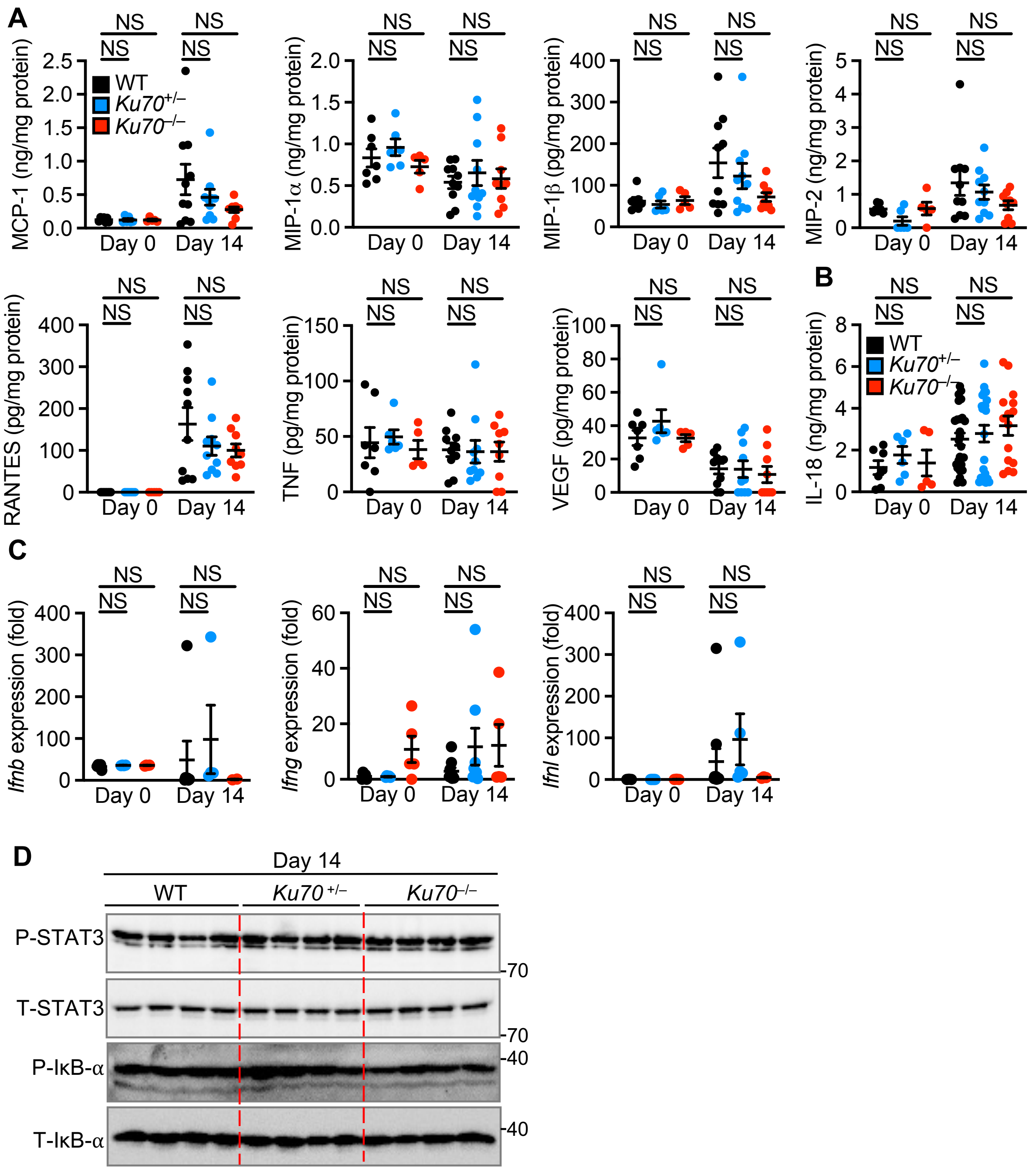


**Fig. S17. Ku70 does not induce the expression of interferons during the development of colitis.**

(**A**) Levels of monocyte chemoattractant protein (MCP)-1, macrophage inflammatory proteins (MIP)-1α, MIP1β, MIP2, regulated on activation, normal T cell expressed and secreted (RANTES), tumor necrosis factor (TNF) and vascular endothelial growth factor (VEGF) in the colon tissue of untreated (day 0) and AOM-DSS treated (day 14) littermate WT, *Ku70*$^{+/–}$ and *Ku70*$^{–/–}$ mice, determined using multiplex ELISA. (**B**) Levels of IL-18 in the colon tissue of untreated (day 0) and AOM-DSS treated (day 14) WT, *Ku70*$^{+/–}$ and *Ku70*$^{–/–}$ mice, determined using simplex ELISA. (**C**) Relative expression of genes encoding interferon (IFN)-β (left), IFN-γ (middle), and IFN-λ (right) in the colon tissue of untreated (day 0) and AOM-DSS treated (day 14) littermate

WT, *Ku70*$^{+/-}$ and *Ku70*$^{-/-}$ mice on the 129 genetic background. (**D**) Immunoblot of the indicated proteins on the colon tissue lysate of littermate WT, *Ku70*$^{+/-}$ and *Ku70*$^{-/-}$ mice at day 14. Each symbol (A to C) and lane (D) represents an individual mouse. NS, not statistically significant by one-way ANOVA with Tukey's multiple comparisons test (A to C). Data are pooled from (A to C) or representative of (D) three independent experiments and are presented as mean ± s.e.m. in (A to C).

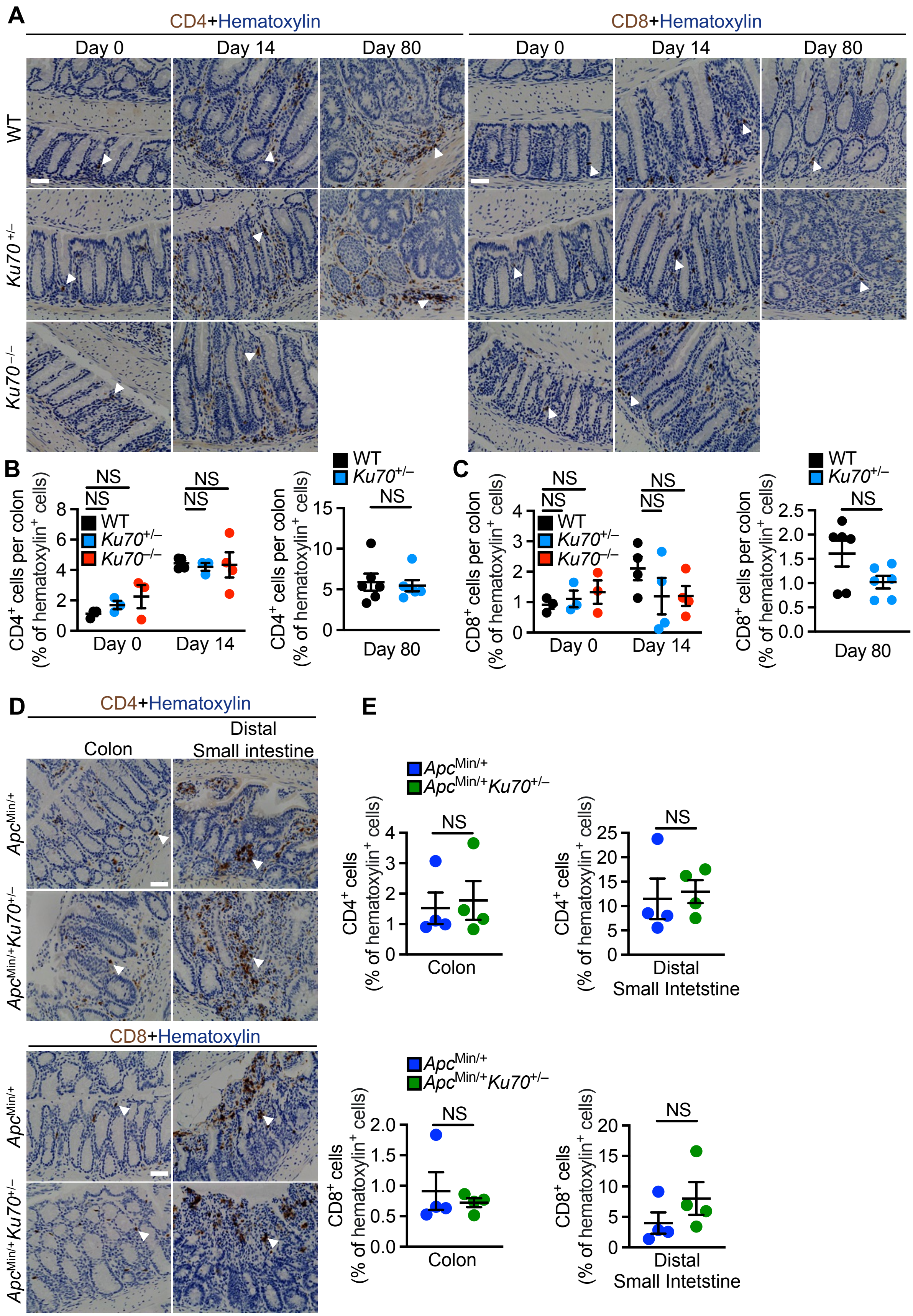
A
CD4+Hematoxylin
CD8+Hematoxylin
Day 0
Day 14
Day 80
WT
Ku70+/–
Ku70–/–
B
CD4+ cells per colon (% of hematoxylin+ cells)
NS
WT
Ku70+/–
Ku70–/–
Day 0
Day 14
Day 80
C
CD8+ cells per colon (% of hematoxylin+ cells)
D
CD4+Hematoxylin
Colon
Distal Small intestine
ApcMin/+
ApcMin/+Ku70+/–
CD8+Hematoxylin
E
CD4+ cells (% of hematoxylin+ cells)
CD8+ cells (% of hematoxylin+ cells)
Colon
Distal Small Intetstine

**Fig. S18. Ku70 may not affect the number of $CD4^+$ and $CD8^+$ cells during the development of colitis and intestinal cancer.** (**A**) Representative images of immunohistochemical staining of CD4 (left) and CD8 (right) in the colon tissue of littermate WT, *Ku70*$^{+/-}$ and *Ku70*$^{-/-}$ mice at day 0 and day 14, and littermate WT and *Ku70*$^{+/-}$ mice at day 80. Scale bar, 50μm. (**B**) Quantification of CD4 in the colon tissue of littermate WT, *Ku70*$^{+/-}$ and *Ku70*$^{-/-}$ mice at day 0 and day 14 (left) and littermate WT and *Ku70*$^{+/-}$ mice at day 80 (right). (**C**) Quantification of CD8 in the colon tissue of littermate WT, *Ku70*$^{+/-}$ and *Ku70*$^{-/-}$ mice at day 0 and day 14 (left) and littermate WT and *Ku70*$^{+/-}$ mice at day 80 (right). (**D**) Representative images of immunohistochemical staining of CD4 (top left) and CD8 (bottom left) in the colon, and CD4 (top right) and CD8 (bottom right) in the distal small intestine of 20 weeks old littermate *Apc*$^{\mathrm{Min}/+}$ and *Apc*$^{\mathrm{Min}/+}$*Ku70*$^{+/-}$ mice. Scale bar, 50μm. (**D**) Quantification of CD4 (top left) and CD8 (bottom left) in the colon, and CD4 (top right) and CD8 (bottom right) in the distal small intestine of 20 weeks old littermate *Apc*$^{\mathrm{Min}/+}$ and *Apc*$^{\mathrm{Min}/+}$*Ku70*$^{+/-}$ mice. Arrowheads indicate positive cells. Each symbol represents an individual mouse in (B, C and E). NS, no significant by one-way ANOVA with Tukey's multiple comparison test (B and C left panels) or by unpaired *t*-test (B and C right panels, and E). Data are presented as mean ± s.e.m. in (B, C and E).

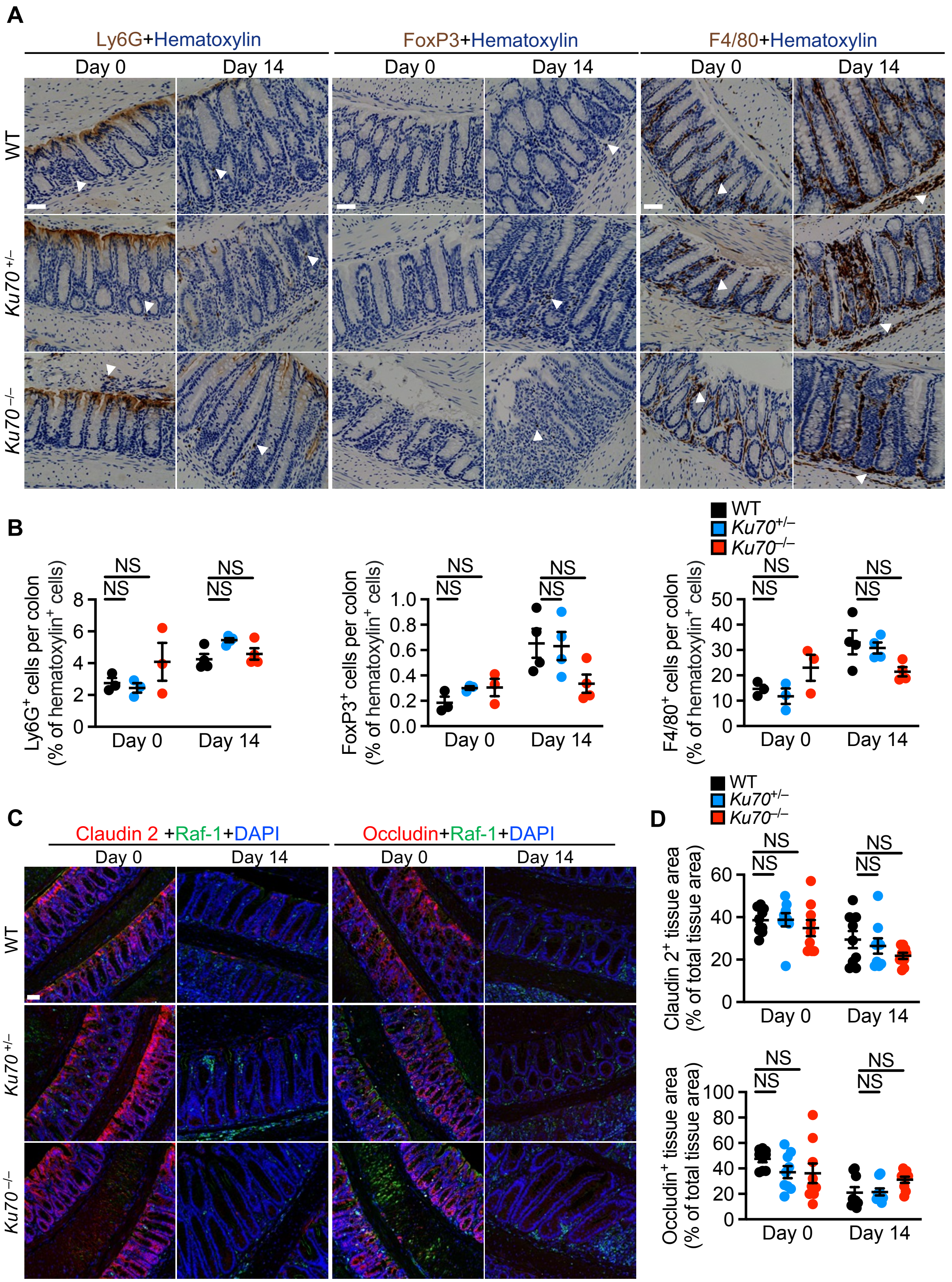
A
Ly6G+Hematoxylin
FoxP3+Hematoxylin
F4/80+Hematoxylin
Day 0
Day 14
WT
Ku70+/–
Ku70–/–
B
WT
Ku70+/–
Ku70–/–
NS
Ly6G+ cells per colon (% of hematoxylin+ cells)
FoxP3+ cells per colon (% of hematoxylin+ cells)
F4/80+ cells per colon (% of hematoxylin+ cells)
C
Claudin 2 +Raf-1+DAPI
Occludin+Raf-1+DAPI
D
Claudin 2+ tissue area (% of total tissue area)
Occludin+ tissue area (% of total tissue area)

**Fig. S19. Ku70 may not affect gut permeability during the development of colitis.** (**A**) Representative images of immunohistochemical staining of Ly6G (left), FoxP3 (middle), and F4/80 (right) in the colon tissue of littermate WT, *Ku70*$^{+/-}$ and *Ku70*$^{-/-}$ mice at day 0 and day 14. Scale bar, 50μm. (**B**) Quantification of Ly6G (left), FoxP3 (middle), and F4/80 (right) in the colon tissue of littermate WT, *Ku70*$^{+/-}$ and *Ku70*$^{-/-}$ mice at day 0 and day 14. (**C**) Representative images of immunohistochemical staining of Claudin 2, β-actin and DAPI (left) and Occludin, β-actin, and DAPI (right) in the colon tissue of littermate WT, *Ku70*$^{+/-}$ and *Ku70*$^{-/-}$ mice at day 0 and day 14. Scale bar, 50μm. (**D**) Quantification of Claudin 2-positive area over total tissue area (top) and Occludin-positive area over total tissue area (bottom) at day 0 and day 14. Arrowheads indicate positive cells in (A). Each symbol represents an individual mouse in (B and C) or one of the three (proximal, middle, or distal) parts of the mouse colon in (D). NS, not significant by one-way ANOVA with Tukey's multiple comparison test (B, C and D). Data are presented as mean ± s.e.m. in (B, C and D).

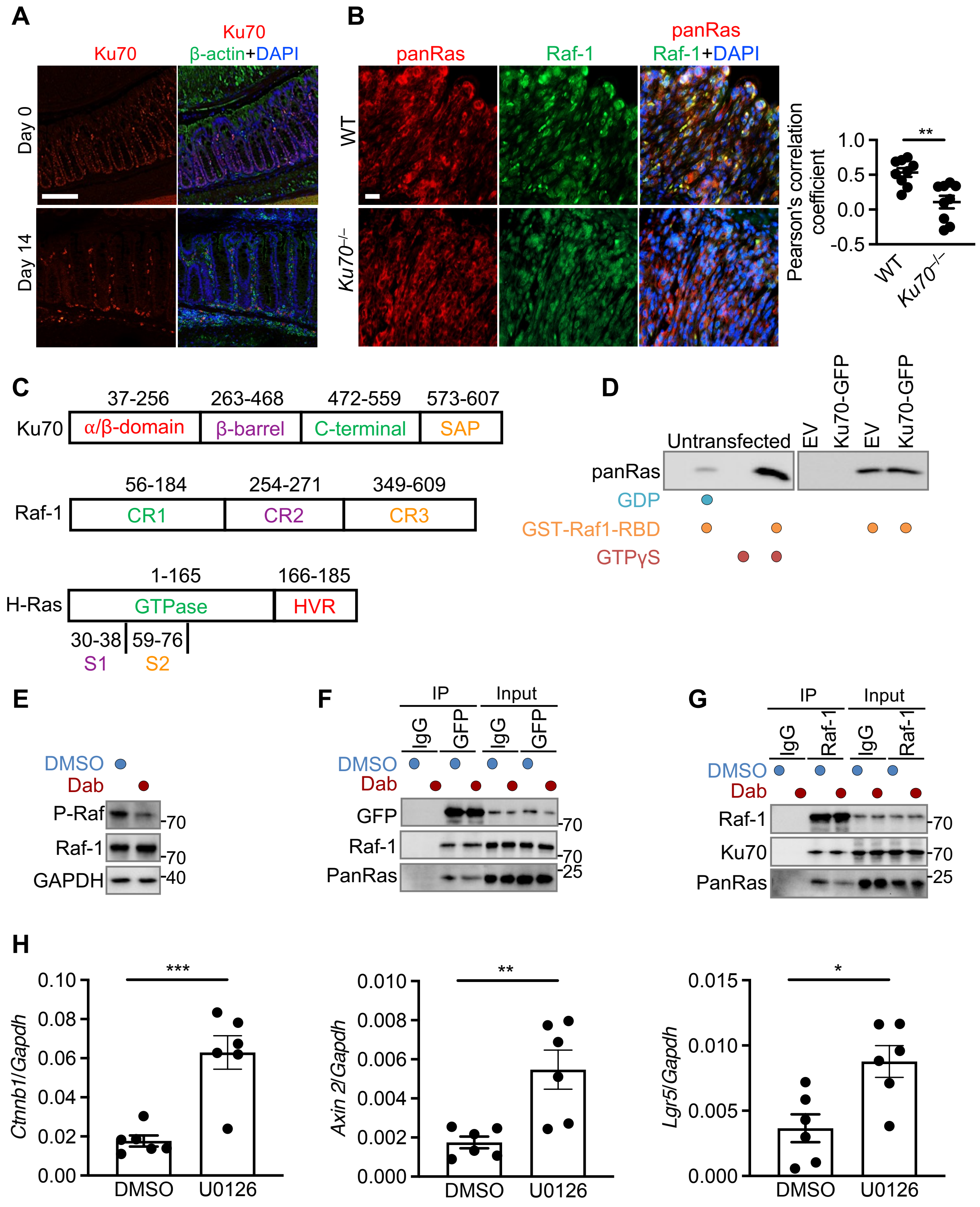


**Fig. S20. Ku70 does not directly contribute to the activation of Ras.** (**A**) Immunohistochemical staining of Ku70, β-actin, and DAPI in the colon tissue of WT mice untreated (Day 0) or treated with AOM-DSS (Day 14). Scale bar, 50μm. (**B**) Immunofluorescence

staining of panRas, Raf-1, and DAPI (left) and frequency of co-localization between panRas and Raf-1 (right) in the colon tissue of WT and *Ku70*$^{-/-}$ mice treated at day 14. Scale bar 20μm. (**C**) Schematic showing domain organization of Ku70 (top), Raf-1 (middle), and H-Ras (bottom). (**D**) HEK293T cell lysates were treated with GTPγS or GDP. The lysates were then incubated with glutathione resin and GST-Raf1-RBD. GTPγS-treated lysate was also incubated without GST-Raf1-RBD in the presence of glutathione resin as a negative control. Cell lysates of HEK293T cells expressing the EV or Ku70 plasmid were incubated with or without GST-Raf1-RBD in the presence of glutathione resin. Immunoblot analysis was performed using a panRas antibody. (**E**) Immunoblot of the indicated proteins on the cell lysate of HEK293T cells left untreated or treated with Dabrafenib (Dab; 2μM), for 2 hours. (**F**) 48 hours after transfection of HEK293T cells with the GFP-tagged Ku70 plasmids, cells were left untreated or treated with Dabrafenib (2μM), for 2 hours and cell lysates were immunoprecipitated (IP) for control (IgG) or GFP and immunoblotted for the indicated proteins. (**G**) 48 hours after transfection of HEK293T cells with the GFP-tagged Ku70 plasmids, cells were left untreated or treated with Dabrafenib (2μM), for 2 hours and cell lysates were immunoprecipitated (IP) for control (IgG) or Raf-1 and immunoblotted for the indicated proteins. (**H**) Relative expression of gene encoding β-catenin (top), Axin 2 (middle), and Lgr5 (right) in colonic organoids of 10-week-old *Apc*$^{Min/+}$ mice. Two days after culture, organoids were treated with vehicle control (DMSO) or U0126 (1μM) for 72 hours. *$P$<0.05; **$P$<0.01; ***$P$<0.001; ****$P$<0.0001 by unpaired *t*-test (B and H). Data are representative of three (A, B, E to G) or two independent experiments in (D and H).

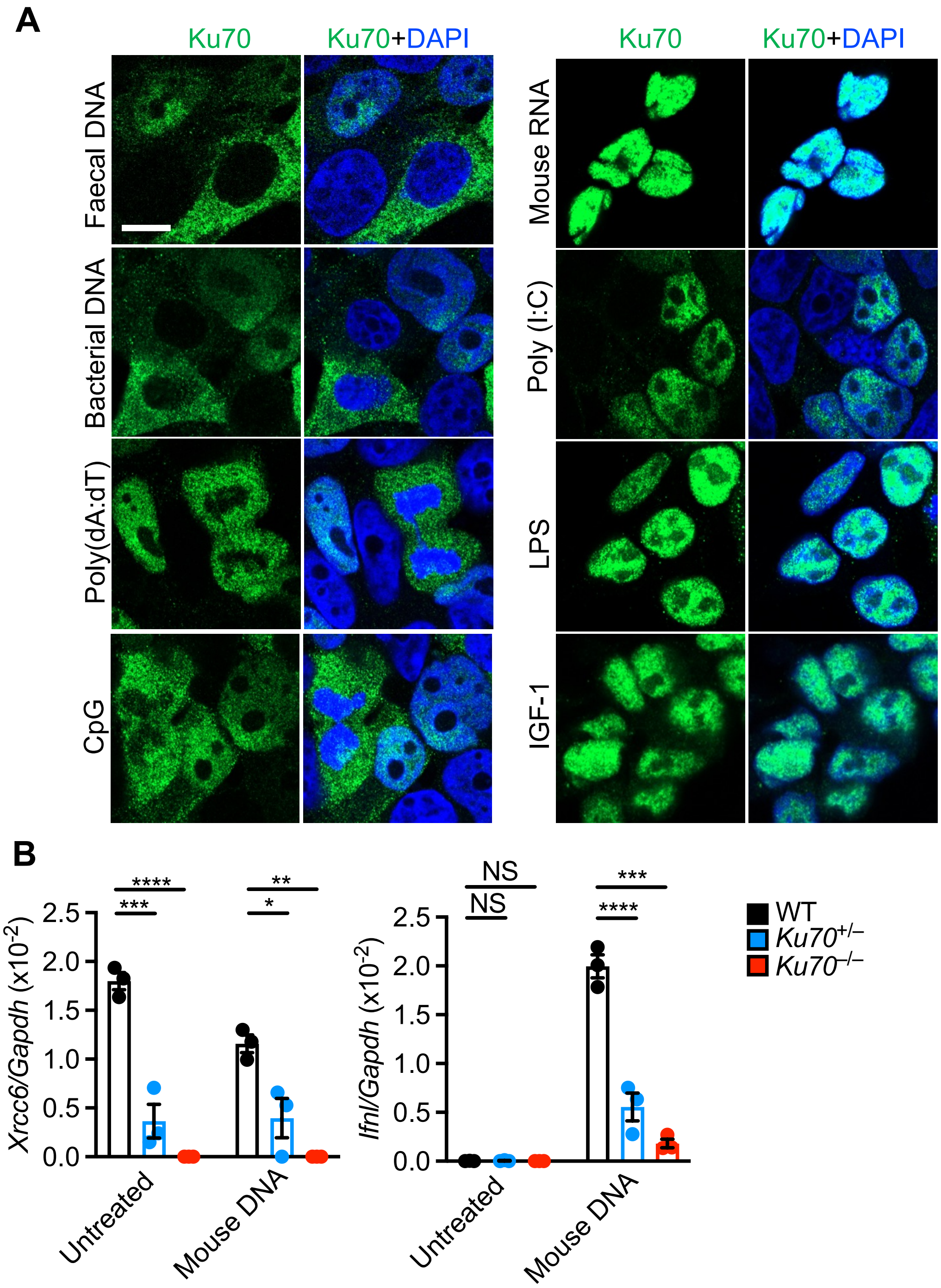


**Fig. S21. Ku70 translocates from the nucleus to the cytoplasm in response to extracellular DNA.** (**B**) Immunofluorescence staining of Ku70 and DAPI in HEK293T cells left non-transfected or transfected with human DNA (1μg), mouse colonic DNA (1μg), mouse fecal DNA (1μg), bacterial DNA (1μg), Poly(dA:dT) (1μg), CpG DNA (1μg), mouse colonic RNA (1μg), Poly(I:C) (1μg) or treated with LPS (1μg) or IGF-1 (50ng). Scale bar: 10 μm. (**C**) Relative expression of genes encoding Ku70 (left) and IFN-λ (right) in the primary ear fibroblast of WT, *Ku70*$^{+/-}$ and *Ku70*$^{-/-}$ mice left non-transfected or transfected with mouse DNA (1μg). *$P$<0.05; **$P$<0.01; ***$P$<0.001; ****$P$<0.0001 by one-way ANOVA with Tukey's multiple comparisons test (B).

Data are representative of three (A) and two independent experiments in (B) and presented as mean ± s.e.m (B).

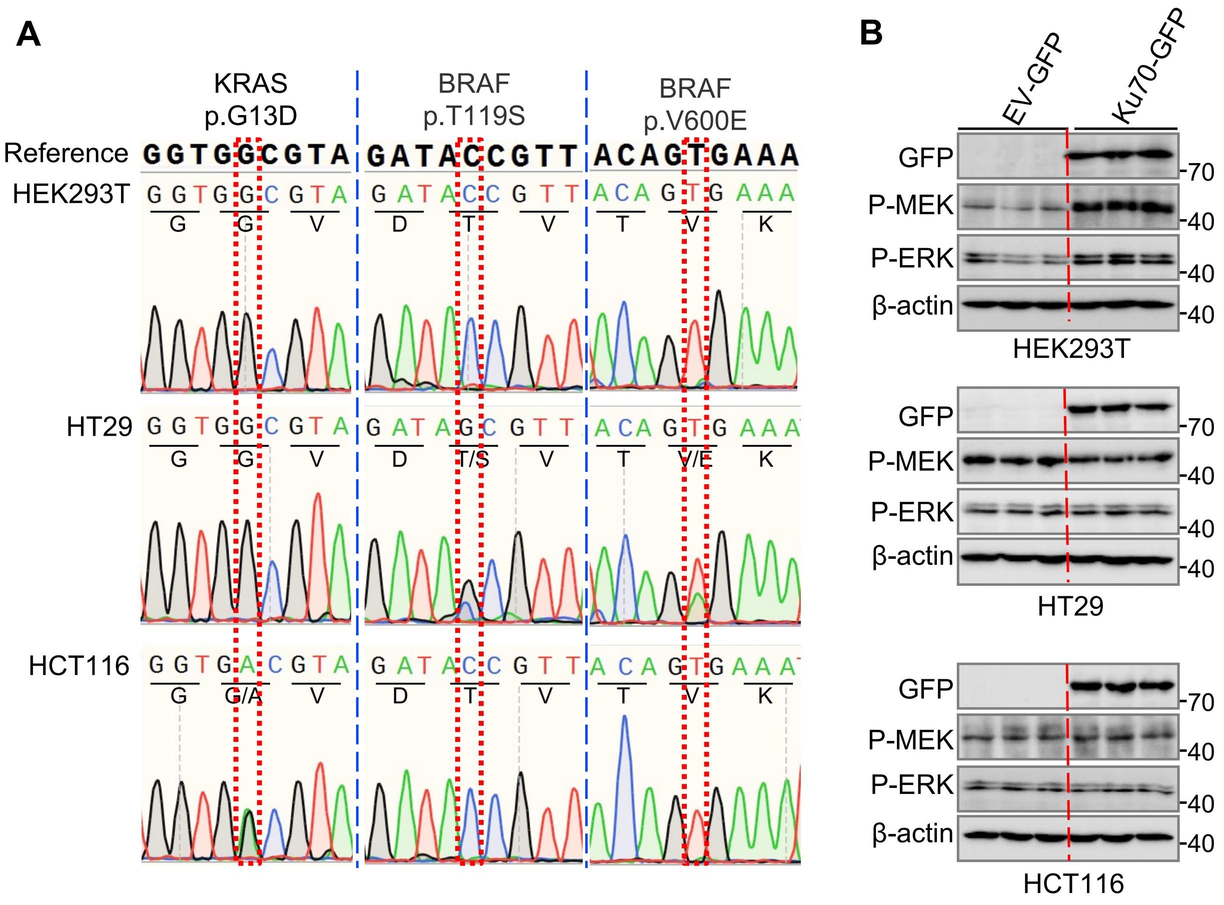


**Fig. S22. Ku70 does not control MEK-ERK signaling in colorectal cancer cell lines harboring KRAS and BRAF mutations.** (**A**) Electropherogram of DNA from Sanger sequencing of the gene encoding KRAS in HCT116 cells, showing a G>A mutation in codon 13 (c.13G; corresponding to p.G13D). The presence of both a G (black) and A (green) peak in codon 13 indicates a heterozygous mutation (Left). Electropherogram of DNA from Sanger sequencing of the gene encoding BRAF in HT29 cells, showing a C>G mutation in codon 119 (c.119T; corresponding to p.T119S). The presence of both G (black) and C (blue) peaks in codon 119 indicates a heterozygous mutation (Middle). Electropherogram of DNA from Sanger sequencing of the gene encoding BRAF in HT29 cells, showing a T>A mutation in codon 600 (c.600V; corresponding to p.V600E). The presence of both T (red) and A (green) peaks in codon 600 indicates a heterozygous mutation (Right). (**B**) Immunoblot of the indicated proteins on the cell lysates of serum-starved HEK293T, HCT116, and HT29 cells transfected with an empty vector (EV) or Ku70 plasmid. The letter underneath the black line below the three nucleotides indicates the corresponding amino acid of the codon (A). Data are representative of three experiments in (B).

**Table S1. List of differentially phosphorylated proteins in the colon tissue of mice with colitis**

| Number of clusters (cluster # from) | Groups | | | Number of proteins |
|---|---|---|---|---|
| | WT | *Ku70*$^{+/-}$ | *Ku70*$^{-/-}$ | |
| 1 | Higher* | Lower** | Lower | 116 |
| 2 | Lower | Higher | Higher | 171 |
| 3 | Higher | Higher | Lower | 45 |
| 4 | Higher | Lower | Higher | 75 |
| 5 | Lower | Higher | Higher | 42 |
| 6 | Lower | Higher | Lower | 60 |
| | | | | Total=509 |

*Higher, higher phosphorylation intensity of a protein in one group as compared to another and/or two other group/s; **Lower, lower phosphorylation intensity of a protein in one group as compared to another and/or two other group/s.

**Table S2. Co-occurrence and mutual exclusivity of mutations in genes encoding Ku70, Ras and Raf in patients with colorectal cancer using cBioPortal.**

| A | B | Neither | A Not B | B Not A | Log2 Odds Ratio* | p-Value | Tendency |
|---|---|---|---|---|---|---|---|
| *XRCC6* | *ARAF* | 1990 | 32 | 48 | >3 | <0.001 | Co-occurrence |
| *XRCC6* | *BRAF* | 1728 | 23 | 310 | 1.955 | <0.001 | Co-occurrence |
| *XRCC6* | *RAF1* | 1977 | 32 | 61 | 2.826 | <0.001 | Co-occurrence |
| *KRAS* | *XRCC6* | 1278 | 760 | 27 | -0.420 | 0.255 | Mutual exclusivity |
| *HRAS* | *XRCC6* | 2017 | 21 | 38 | 1.338 | 0.342 | Co-occurrence |
| *NRAS* | *XRCC6* | 1909 | 129 | 36 | 0.302 | 0.456 | Co-occurrence |

*A positive value for the Log2 Odds Ratio suggests that alterations in these genes co-occur in the same samples, while a negative value suggests that alterations in these genes are mutually exclusive and occur in different samples.

**Table S3. List of qRT-PCR primers**

| Gene | Primer | Sequence | Reference |
|---|---|---|---|
| *mGapdh* | *Gapdh*F | 5'-CGT CCC GTA GAC AAA ATG GT-3' | *(5)* |
| | *Gapdh*R | 5'-TTG ATG GCA ACA ATC TCC AC-3' | |
| *mXrcc6* | *Xrcc6*F | 5'-ATGTCAGAGTGGGAGTCCTAC-3' | Harvard PrimerBank |
| | *Xrcc6*R | 5'-TCGCTGCTTATGATCTTACTGGT-3' | |
| *mIfnb* | *Ifnb*F | 5'-GCC TTT GCC ATC CAA GAG ATG C-3' | *(95)* |
| | *IfnbR* | 5'-ACA CTG TCT GCT GGT GGA GTT C-3' | |
| *mIfng* | *Ifng*F | 5'-GCA TCT TGG CTT TGC AGC T-3' | *(95)* |
| | *IfngR* | 5'-CCT TTT TCG CCT TGC TGT TG-3' | |
| *mIfnl* | *Ifnl*F | 5'-AGC TGC AGG TCC AAG AGC G-3' | *(97)* |
| | *IfnlR* | 5′-GGT GGT CAG GGC TGA GTC ATT-3' | |
| *mAtm* | *AtmF* | 5′- ATC CCT TGT GTG TTC TCT G −3′ | *(98)* |
| | *AtmR* | 5′- CGC CTC TGC TGT CGT GTA T −3′ | |
| *mChk1* | *Chk1F* | 5′-CTG GGA TTT GGT GCA AAC TT-3′ | *(99)* |
| | *Chk1R* | 5′-GCC CGC TTC ATG TCT ACA AT-3′ | |
| *mRad51* | *Rad51F* | 5'AGGTGGATCCGCTATGCAAATGCAGC-3' | *(100)* |
| | *Rad51R* | 5'-TCCAGAATTCCTGCCGTGGTGAAACCCAT- 3' | |
| *mCtnnb1* | *mCtnnb1-F* | 5'-ATG GAG CCG GAC AGA AAA GC-3' | *(100)* |
| | *mCtnnb1-R* | 5'-CTT GCC ACT CAG GGA AGG A-3' | |
| *mAxin2* | *mAxin2-F* | 5'- ACC AGG ATG GTG CAT ACC TCT -3' | *(100)* |
| | *mAxin2-F* | 5'- ACC AGG ATG GTG CAT ACC TCT -3' | |
| *mLgr5* | *mLgr5-F* | 5'- CCT ACT CGA AGA CTT ACC CAG T -3' | *(100)* |
| | *mLgr5-R* | 5'- GCA TTG GGG TGA ATG ATA GCA -3' | |
